# A protective personal factor against disability and dependence in the elderly: an ordinal regression analysis with nine geographically-defined samples from Spain

**DOI:** 10.1186/s12877-016-0409-9

**Published:** 2017-01-31

**Authors:** Javier Virues-Ortega, Saturio Vega, Manuel Seijo-Martinez, Pedro Saz, Fernanda Rodriguez, Angel Rodriguez-Laso, Susana Perez de las Heras, Raimundo Mateos, Pablo Martinez-Martin, Ignacio Mahillo-Fernandez, Josep Garre-Olmo, Jordi Gascon, Francisco Jose Garcia-Garcia, Manuel Fernandez-Martinez, Felix Bermejo-Pareja, Alberto Bergareche, Julian Benito-Leon, Jesus de Pedro-Cuesta, on behalf of the Spanish Epidemiological Studies on Ageing Group

**Affiliations:** 1 National Centre for Epidemiology and CIBERNED, Carlos III Institute of Health, Madrid, Spain; 2 School of Psychology, The University of Auckland, Room 335B Level 3 Bldg 301 Science Centre 23 Symonds St Private Bag 92019, Auckland, 1010 New Zealand; 3 Arévalo Health Centre, Avila, Spain; 4 Neurology Unit, Salnés Hospital, Pontevedra, Spain; 5 Department of Medicine and Psychiatry, Zaragoza University, Zaragoza, Spain; 6 Neurology Unit, Segovia Hospital, Segovia, Spain; 7 Madrid Regional Health Authority, Madrid, Spain; 8 Llodio Health Center, Day Medical Center, Oroityu-Getxo-Vizcaya, Spain; 9 Psychiatry Department, University of Santiago de Compostela, Santiago de Compostela, Spain; 10 Girona Biomedical Research Institute Institut d’Assistència Sanitària, Salt, Spain; 11 Dementia Diagnosis and Treatment Unit, Neurology Department, Bellvitge University Teaching Hospital, Barcelona, Spain; 12 Geriatrics Unit. Virgen del Valle Geriatric Hospital, Toledo, Spain; 13 Neurology Unit, Virgen del Camino Hospital, Pamplona, Spain; 14 Neurology Department, 12 de Octubre University Teaching Hospital and CIBERNED, Madrid, Spain; 15 Department of Neurology, Hospital Universitario Donostia, San Sebastián Guipuzcoa, Spain; 16 Biodonostia Research Institute, Area of Neurosciences, and CIBERNED, Carlos III Health Institute, Madrid, Spain

**Keywords:** Sense of coherence, Disability, Dependence, Katz index, WHODAS 2.0

## Abstract

**Background:**

Sense of Coherence (SOC) is defined as a tendency to perceive life experiences as comprehensible, manageable and meaningful. The construct is split in three major domains: Comprehensibility, Manageability, and Meaningfulness. SOC has been associated with successful coping strategies in the face of illness and traumatic events and is a predictor of self-reported and objective health in a variety of contexts. In the present study we aim to evaluate the association of SOC with disability and dependence in Spanish elders.

**Methods:**

A total of 377 participants aged 75 years or over from nine locations across Spain participated in the study (Mean age: 80.9 years; 65.3% women). SOC levels were considered independent variables in two ordinal logistic models on disability and dependence, respectively. Disability was established with the World health Organization-Disability Assessment Schedule 2.0 (36-item version), while dependence was measured with the Extended Katz Index on personal and instrumental activities of daily living. The models included personal (sex, age, social contacts, availability of an intimate confidant), environmental (municipality size, access to social resources) and health-related covariates (morbidity).

**Results:**

High Meaningfulness was a strong protective factor against both disability (Odds Ratio [OR] = 0.50; 95% Confidence Interval [CI] = 0.29–0.87) and dependence (OR = 0.33; 95% CI = 0.19–0.58) while moderate and high Comprehensibility was protective for disability (OR = 0.40; 95% CI = 0.22–0.70 and OR = 0.39; 95%CI = 0.21–0.74), but not for dependence. Easy access to social and health resources was also highly protective against both disability and dependence.

**Conclusions:**

Our results are consistent with the view that high levels of SOC are protective against disability and dependence in the elderly. Elderly individuals with limited access to social and health resources and with low SOC may be a group at risk for dependence and disability in Spain.

**Electronic supplementary material:**

The online version of this article (doi:10.1186/s12877-016-0409-9) contains supplementary material, which is available to authorized users.

## Background

There seems to be a growing interest in the literature in understanding how personal factors impact chronic diseases and disability [1]. Greater attention has been devoted to behavioral risk factors (e.g., anger, type A behavior pattern), as opposed to protective behavioral factors. Among the latter, resilience, hardiness and sense of coherence (SOC) have been associated with self-reported health, sickness recovery, and protection against sickness [2]. Therefore, protective behavioral factors are heuristically valuable constructs to approach the connection between health and behavior [3].

A. Antonovsky developed an approach to the origin and maintenance of health based on personal cognitive factors and defined SOC as the key of his theory. Sense of coherence is defined as “a global orientation that expresses the extent to which one has a pervasive, enduring though dynamic feeling of confidence that (a) the stimuli deriving from one’s internal and external environments in the course of living are structured, predictable, and explicable (comprehensibility); (b) the resources are available to one to meet the demands posed by these stimuli (manageability); and (c) these demands are challenges, worthy of investment and engagement (meaningfulness)” [4].

Over the last decades hundreds of empirical studies have related SOC to health variables such as psychological well being, adaptive coping strategies, social support as well as self-reported and clinically-assessed physical health [5–7]. Very few studies, however, have focused on the relationship between a strong SOC and specific health dimensions in elderly populations such as self-reported health and functional status. Schneider et al. [8] reported that SOC exerts an independent positive influence towards self-reported health among a sample of geriatric patients. Another study showed that individuals with stronger SOC kept their functional status for longer relative to individuals with weaker SOC [9]. Read et al. [10] reported that SOC was significantly associated with physical, social and mental health in a probabilistic sample of 65–69 year-old participants. Moreover, SOC has been linked to adherence to rehabilitation programs among elder patients living at home [11], and with an approximately 30% reduction in mortality, including mortality due to cardiovascular disease and cancer [12].

The International Classification of Functioning, Disability and Health (ICF) provides a general framework to conceptualize and classify complex interactions of environmental and personal factors over functioning and disability [13]. While there is a growing repertoire for the assessment and interpretation of factors pertaining to physical health and social behavior, both in terms of standardized instruments and disease-specific core sets of assessment areas [14], behavioral factors are still ill-informed as far as evaluation tools and outcome-specific classifications [15]. Thus, exploring SOC-specific disability patterns may be highly informative for the purposes of care and health services planning. Similar evidence has been reported for socio-demographic variables, which have been found predictive of different long-term functional outcomes [16]. A valuable step toward this aim would be to examine the functional relationship between SOC and disability with methods consistent with the ICF framework.

The purpose of this study was to quantify the relationship of SOC domains along with selected social and environmental factors over disability and dependence. Disability and dependence levels were estimated by means of the World Health Organization Disability Assessment Schedule [17] and the Extended Katz Index of dependence [18], respectively. We hypothesize that relatively high scores in SOC domains will be associated with lower levels of disability and dependence in the people older than 75.

## Methods

### Study sample

This report is part of a study on aging concerned with physical and mental health, social participation, quality of life and socio-demographic aspects in people older than 75 in Spain. The sample was composed of nine probabilistic and geographically-defined sub-samples from different locations of Central and Northern Spain. Recruitment and data collection took place between June 2005 through December 2005. We recruited surviving participants from previous dementia prevalence studies conducted in Spain [19]. Participants should be aged 75 or older, have an score in the MMSE > 23, and should not have a clinical history of dementia [20], and have usable data in all predictive and outcome variables. Further details on the sampling method and the sample composition have been made available elsewhere [19]. From an original study sample of 546 participants, only participants with computable SOC, disability and dependence data (69.0%) were included in the present study. Responding to the questionnaire measuring SOC was considered voluntary. A total of 433 individuals were aged above 75 and had an MMSE > 23 [19]. Among these, 56 individuals were excluded due to a history of neurological disease. A total of 377 individuals (mean age: 80.9; 65.3% women) had complete datasets for the purposes of the current analysis and met all inclusion criteria. The health-related and socio-demographic characteristics of the study sample are shown in Table 1. Detailed reports on dementia and disability prevalence as well as to disability-associated health conditions in the 546 participants have been reported elsewhere [19, 21, 22]. The dataset upon which the current analysis is based is available upon request.

**Table 1:** Socio-demographic characteristics and morbidity (*n* = 377)

		Percent (Number)
Age		
75–79		
	Women	30.5 (115)
	Men	16.2 (61)
80–84		
	Women	16.5 (62)
	Men	11.4 (43)
≥ 85		
	Women	18.3 (69)
	Men	7.2 (27)
Self-reported social class		
	Low	9.3 (35)
	Middle-low	27.3 (103)
	Middle	52.8 (199)
	Middle-high	10.6 (40)
Level of instruction		
	Illiterate	9.3 (35)
	Primary incomplete	40.9 (154)
	Primary complete	32.1 (121)
	Some secondary or higher	17.8 (67)
Morbidity (ICD 10 codes)		
	Mental and behavioral (F00-F99)	26.3 (99)
	Neurological including stroke (G00-G99, I60-I69)	14.1 (53)
	Cardiovascular (I00-I52, I70-I99)	67.9 (256)
	Eye (H00-H59)	37.9 (143)
	Musculoskeletal and injuries (M00-M99; S00-S9)	48.8 (184)

### Procedure

Data was derived from structured interviews administered during two successive visits to the individual’s home. The first visit covered objective and self-reported aspects of health. It was carried out by a licensed physician trained in neurology, psychiatry or geriatrics (average visit duration: 1.5 h). The second visit was scheduled within the two-week period following the first (original data collection questionnaires have been provided as Additional file 1). This visit focused on self-reported aspects of functioning and disability in addition to psychological and social factors and was conducted by a purposely trained health professional whether physician, nurse or psychologist (average visit duration: 1 h). All variables reported here, with the exception of inclusion criteria and morbidity, were collected during the second interview. The assessments relevant to this study are described below.

#### Antonovsky’s sense of coherence (SOC)

The Orientation to Life Questionnaire (OLQ-13) consists of 13 items covering the three domains of the SOC construct: meaningfulness (score range: 4-28), comprehensibility (score range: 5-35), manageability (score range: 4-28) [4]. The items are scored over a seven-point Likert scale (e.g., 1 = never, 7 = always). Negatively-worded items are reverse coded (items 1, 2, 3, 7, 10). Higher scores indicate higher levels in either of the three SOC domains. The scale has shown appropriate internal consistency and high construct and cross-cultural validity [23]. The OLQ-13 has been adapted to the Spanish elderly population [24]. The three domains of the scale were scored separately for all analyses in this study owing to the empirical evidence supporting the multidimensional nature of the construct [9, 24].

#### World health organization disability assessment schedule (WHO-DAS-II)

This is a self-reported 36-item questionnaire covering six disability domains: Participation in Society, Life Activities, Getting Along with Others, Self-Care, Getting Around and Understanding and Communication. Items are answered over a 5-point Likert scale depending on the difficulty of the participant performing a given activity over the last 30 days (1: None; 5: Extreme). Score per domain and total questionnaire score range from 0 to 100 with higher scores indicating higher disability. Life activities domain was omitted in those individuals with no household duties assigned. In addition, item D4.5 on sexual relationships was excluded from the total score computation as 47.1% of participants declined to answer. Items on work performance were also omitted as all participants were retired. WHODAS 2.0 total score was transformed into disability categories according to ICF disability levels established over a 100-point scale [13]: No problem (0–4%), Mild problem (5-24%), Moderate problem (25–49%), Severe problem (50-95%), Extreme problem (95–100%). Table 2 presents selected characteristics of this instrument.

**Table 2:** Characteristics of WHODAS 2.0 and the extended Katz index

	WHO-DAS II	Extended Katz index
Selected validation	Ustün et al. [17]	Asberg&Sonn [18]
Target construct	Self-reported disability	Objective dependence in activities of daily living
Domains	Understanding and communication; Getting around; Self-care; Getting along with others; Life activities; Participation in society	Personal activities of daily living (bathing, dressing, toileting, transferring, continence, feeding); Instrumental activities of daily living (shopping, cleaning, transportation, washing, cooking)
Number of items	32	11
Response levels	No difficulty; mild difficulty; moderate difficulty; severe difficulty; extreme difficulty/cannot do it	Independent; partly dependent; dependent
Score range	0–100	Rationally defined dependence levels
Outcome levels in the present study	No problems (0–4); Mild disability (5–24); Moderate disability (25–49); Severe disability (50–95); Extreme disability (96–100)	Independent, Dependent in instrumental activities of daily living; dependent in personal activities of daily living

*Note.* WHODAS 2.0 items on work performance were not used in this study

#### Katz extended index of dependence in activities of daily living (extended Katz index)

The Extended Katz Index is a 11-item measure assessing dependence (need of help). It covers six personal basic activities of daily living (BADL) (bathing, dressing, toileting, transferring, continence, and feeding) and five instrumental activities of daily living (IADL) (shopping, cleaning, transportation, washing, and cooking) [25, 26]. Items are scored over a 3-point scale: independent, in case the individual can carry out the skill without support; partly dependent, if the individual is able to complete the activity with help; and dependent, when the individual is not able to perform the skill even with help from others. Scale scores follow a cumulative structure [27]. Descriptive features of the Extended Katz Index are presented in Table 2.

#### Social support

Participants’ structural social support was explored through the median frequency of personal contacts with children, extended family and friends in a 5-point Likert scale (1: never; 5: daily contacts). In addition, the availability of an intimate confidant was recorded as an index of emotional social support.

#### Accessibility to social and health resources

This variable was measured as the median accessibility over a 5-point scale (1: very easy access; 5: very difficult access) to six social resources: health center/medical attention, public transportation, public leisure resources (garden, park), shops, social clubs/cultural resources, and religious services.

#### Municipality size

The number of inhabitants of the population where individuals were de facto residents were recorded and coded according to the census and categories of the Spanish National Institute of Statistics: Rural-Intermediate (1–10,000 inhabitants), and Urban (>10,000 inhabitants).

#### Morbidity

Morbidity was measured as the sum of current medical conditions. Medical conditions were surveyed through a checklist of 51 diseases prevalent in the elderly. Additional conditions not identified in the checklist were also recorded (ICD-10 codes specified a posteriori). The presence of medical conditions was established on the basis of primary care medical records, other medical records provided by the examinee, physical examination, and self- and proxy-reported conditions. This section was administered by a licensed physician and was later supervised by a senior physician. Diagnoses of those at chronic care facilities, nursing homes and psychiatric hospitals were informed by medical records and medical staff at those sites. Diagnosis of dementia and dementia subtypes were conducted by two independent licensed neurologists.

### Statistical analysis

Ordinal logistical regression was used to study the effect of multiple independent variables over disability and dependence. International Classification of Functioning disability categories based on WHO-DAS-II scores, and dependence levels based on Extended Katz Index were considered as separate dependent variables.

Ordinal regression generates a single odd ratio for all ordinal levels within a dependent variable. Thus, the current analysis offsets the need to specify an interval within the target dependent variable for the purposes of case definition. The ordinal intervals for disability were defined following the ICF severity categories: No problem (NP); Mild disability (MILD); Moderate, severe and extreme disability (MO/SE/EX). Fewer individuals were allocated to the three highest ICF disability category, which motivated these three categories to be amalgamated into the MO/SE/EX category. We defined three ordinal intervals for dependence: independent (no dependence in either instrumental or personal ADL), IADL dependent (dependent in one or more IADL but independent in personal ADL), and ADL dependent (dependent in one or more ADL).

Associations between predictive factors and the outcome variables were expressed as odds ratios (OR) and confidence interval (95% CI). The criterion for statistical significance was set at 5%. In order to obtain relatively symmetric disability levels, moderate, severe and extreme, ICF disability categories were collapsed. Tests for proportional odds were performed to check the homogeneity of the effects across adjacent categories of the dependent variables.

In order to provide a highly specific characterization of the association between SOC, and disability and dependence, scores of Comprehensibility, Manageability, and Meaningfulness were introduced into the model as independent predictors in two separate models, one for of the outcomes. Low, mid, and high levels of Comprehensibility, Manageability, and Meaningfulness scores were established by tertiles using the interval defined by the range between the lowest score and tertile 1 as the reference category. While there is no widely accepted cut-off points for SOC domain levels [23, 28], the current approach is a conservative in that it is purely based on scores distribution. Owing to the size of our sample, it is unlikely that the current approach would exclude any socially valid level of a particular SOC domain. In order to provide a multi-faceted approach to disability in line with the conceptual basis provided by the ICF system, we introduced selected social (social network, availability of confidant), and environmental variables (access to social resources, municipality size), whose influence on disability is well documented [1, 13, 15]. Finally, for control purposes both models were adjusted for age, sex and morbidity (number of ICD-10 diagnoses).

## Results

Counts of participants for each of the ordinal intervals within each of the dependent variables in the analyses are presented in Tables 3. Table 4 summarizes the final ordinal logistic regression models for disability (WHO-DAS II) and dependence (Extended Katz Index). Age, sex and morbidity were non-significant for all analyses with the exception of sex for disability. Specifically, men were more likely to be at a higher disability level (OR = 2.01, 95% CI 1.28–3.17). Odd proportional tests were non-significant for the two models, suggesting that effects were proportional across the categories of the outcome variables.

**Table 3:** Distribution of participants across predictors and outcome levels

	ICF WHODAS 2.0 (*n* = 377)			ADL (*n* = 377)		
	NP	MILD	MO/SE/EX	IND	IADL	PADL
Sex						
Women	84	98	64	96	102	48
Men	75	40	16	53	55	23
Age						
75–79	85	69	22	91	64	21
80–74	45	42	18	41	40	24
≥ 85	29	27	40	17	53	26
Comprehensibility						
Low	34	53	36	47	47	29
Mid	58	45	19	50	53	19
High	67	40	25	52	57	23
Manageability						
Low	40	55	31	54	49	23
Mid	51	44	23	41	52	25
High	68	39	26	54	56	23
Meaningfulness						
Low	28	53	40	36	54	31
Mid	42	39	20	37	40	24
High	89	46	20	76	63	16
Confidant available						
Yes	144	114	54	130	130	52
No	15	24	24	19	27	19
Social contacts						
≤ Monthly	22	20	20	17	31	14
Biweekly	29	27	14	27	25	18
Weekly	53	47	24	47	59	18
Everyday	55	44	22	58	42	21
Social resources access						
Very difficult	1	3	16	0	6	14
Difficult	1	4	15	2	13	5
Neutral	3	5	8	6	7	6
Easy	76	80	26	60	90	32
Very easy	78	43	15	81	41	14
Town size						
1–10,000 hab.	50	45	43	59	51	28
> 10,000 hab.	109	93	37	90	106	43

*Notes. NP* No problem, *MILD* Mild disability, *MO/SE/EX* Moderate, severe and extreme disability, *IND* Independent in personal and instrumental activities of daily living, *IALD* Dependent in one or more instrumental activity of daily living only, *PADL* Dependent in one or more personal activity of daily living

**Table 4:** Summary of ordinal logistic regression analyses for disability and dependence

	WHODAS 2.0 (*n* = 377)			Extended Katz index (*n* = 377)		
	OR	95% CI	*p*	OR	95% CI	*p*
Target predictors						
Comprehensibility (Low)						
Mid	0.40	0.22–0.70	0.002	0.82	0.47–1.44	ns
High	0.39	0.21–0.74	0.004	0.79	0.43–1.47	ns
Manageability (Low)						
Mid	1.30	0.72–2.36	ns	1.95	1.09–3.51	0.025
High	0.76	0.40–1.44	ns	1.31	0.70–2.46	ns
Meaningfulness (Low)						
Mid	0.63	0.36–1.10	ns	0.88	0.50–1.53	ns
High	0.33	0.19–0.58	0.000	0.50	0.29–0.87	0.014
Social factors						
Availability of confidant (No)						
Yes	0.56	0.31–1.01	0.054	0.87	0.49–1.53	ns
Social contacts (Once a month or less)						
Biweekly	1.12	0.54–2.31	ns	1.09	0.54–2.19	ns
Weekly	0.99	0.53–1.88	ns	0.78	0.42–1.45	ns
Everyday	0.70	0.36–1.36	ns	0.70	0.37–1.33	ns
Environmental factors						
Social resources accessibility (Very difficult)						
Difficult	1.44	0.30–6.91	ns	0.23	0.06–0.86	0.029
Not difficult nor easy	0.18	0.04–0.84	0.029	0.14	0.04–0.56	0.006
Easy	0.06	0.02–0.20	0.000	0.07	0.02–0.22	0.000
Very easy	0.04	0.01–0.13	0.000	0.03	0.01–0.99	0.000
Municipality size (1–10,000 hab.)						
> 10,000 habitants	0.50	0.30–0.84	0.003	1.84	1.11–3.05	0.018

*Notes.* Models adjusted for sex, age, and morbidity. Reference category in parenthesis. Data were split by tertiles in order to obtain low, mid, and high levels in each of the SOC domains. WHO-DAS-II outcome levels: no problem, mild, moderate or above. Extended Katz Index output levels: independent, instrumental activities dependent, basic activities dependent. No significant departures from the proportional odds assumption were detected for any outcome (*p* > 0.2). ns = *p* > 0.05

**Fig. 1:**
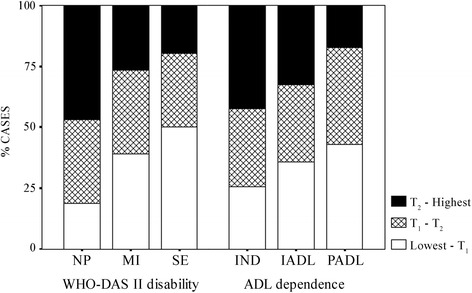
Percentage of cases with low, mid and high Meaningfulness across disability and dependence levels. Notes. Comprehensibility levels established by tertiles (T_1_ = Tertil 1, T_2_ = Tertil, T_3_ = Tertil 3). NP = No problem; MI = Mild problem; SE = Moderate, severe or extreme problem; IND = Independent; IADL = Dependent in instrumental activities; PADL = Dependent in basic activities

Mid and high Comprehensibility levels were highly protective against disability and dependence (OR = 0.40, 95% CI 0.22–0.70; OR = 0.39, 95% CI 0.21–0.74). High Meaningfulness was also highly protective for both disability and dependence (OR = 0.33, 95% CI 0.19–0.58; OR = 0.50, 95% CI 0.29–0.87; see also Fig. 1).

The frequency of social contacts remained a non-significant protective predictor for disability and dependence. The availability of an intimate confidant demonstrated a significant protective effect against disability (OR = 0.56, 95% CI 0.31–1.01). Access to social resources showed a dose-dependent protective effect against both disability and dependence (OR = 0.18–0.04; OR = 0.14–0.03; see Table 4). Living in municipalities with 10,000 inhabitants or more was a protective factor against disability as established by WHO-DAS-II (OR = 0.50, 95% CI 0.30–0.84), whereas it was a risk factor for dependence in activities of daily living in the same individuals (OR = 1.84, 95% CI 1.11–3.05).

## Discussion

This is the first ordinal regression analysis conducted in a population-based sample exploring the properties of SOC as a predictor of both disability and dependence controlling for major socio-demographic and physical health determinants. Results indicated that individuals with a strong SOC, and particularly a high Meaningfulness score, are protected against disability, as measured by WHO-DAS II, and against dependence for both basic and instrumental ADL, as measured by the Extended Katz Index. In addition, individuals with average to high Comprehensibility were protected from self-reported disability (WHO-DAS II). These results remained consistent across the three severity levels of both outcome variables in the ordinal logistic regression models. Environmental factors were also found to be associated with disability. Easy access to social and health services was an independent protective factor reducing the risk of disability and dependence. Finally, living in urban areas was a protective factor against self-reported disability and a risk factor for dependence in activities of daily living, respectively.

A few limitations to the current analysis should be noted. First, the exclusion of participants with non-computable SOC scores may have selected participants with less disability and higher SOC. While the SOC scores of lost participants could not be verified, the proportion of participants with moderate to extreme disability was higher among excluded participants due to non-computable SOC (87.38 vs. 57.83%). Moreover, a number of individuals diagnosed with dementia in the original sample did not have computable SOC scores (24 out of 41). This is to be expected as higher cognitive skills are needed to complete the OLQ-13 reliably and such skills may be impaired among individuals with early stage dementia [29].

Second, unknown causes of disability co-varying with SOC may distort the ordinal regression models presented here by way of overestimating the association between target independent and dependent variables. Specifically, depression and dementia are - powerful determinants of disability in the elderly [19] and may be potential factors driving overestimation. Preliminary analyses showed that SOC was not associated with a clinical diagnosis of depression in the current dataset. In addition, our results remained unchanged by the exclusion of individuals with dementia. A more crude, yet valid approach to minimize overestimation might have been to include total comorbidity as a covariate.

In order to account for the differential pattern of findings across WHODAS 2.0 and Extended Katz Index, two differential features of these instruments should be brought to bear. First, the Extended Katz Index focuses on basic behavioral repertoires as opposed to WHO-DAS-II, which emphasizes advanced motor, cognitive and social skills. Second, the Extended Katz Index is scored objectively by a health professional and is aimed at informing the individual’s need of help regardless of environmental factors (e.g., adaptations, caregiver services). By contrast, WHODAS 2.0 is a self-reported instrument focused on the self-reported difficulty in the performance of a set of activities factoring in personal and environmental factors alike. Moreover, there is evidence to suggest that self-reported versus performance-based measures of functional status and activities of daily living correlate poorly [30, 31]. Thus, WHODAS 2.0 and the Extended Katz Index characterize different facets of the individual’s functional status.

Our study suggests that SOC, and particularly Meaningfulness, has a protective effect against dependence and disability. This effect remained significant after controlling for sex, age, morbidity, and selected environmental and social factors. In addition, low Comprehensibility and large municipality size were risk factors for dependence. A separate analysis showed that the effect of municipality size on dependence was mainly driven by instrumental ADLs. This finding is consistent with the definition of Comprehensibility, which is associated with the complexity of the individual’s environment. Therefore, Comprehensibility may be associated with the difficulties imposed by the urban environment in performing selected instrumental activities (e.g., using public transportation).

On the other hand, health and social resources were less accessible in small towns (difficult and very difficult access: 25.4% vs. 2.1%). The fact that social resources are more readily available in the urban habitat may explain why the WHODAS 2.0 is affected differently by municipality size. Specifically, WHODAS 2.0 focuses more in social participation activities, which may have established urban residence as a protective factor for disability.

The causal pathways through which these effects operate require further empirical analysis before a more conceptually systematic interpretation of our data is possible. First, higher SOC may be highly correlated with health behaviors, which would in turn cause more favorable disability and health-related outcomes. In fact, individuals with a strong SOC demonstrate a higher adherence to medical treatments and rehabilitation programs and, devote more time to leisure and physical exercise [11, 32–35]. Additionally, a longitudinal study by Sulander et al. [36] showed that poor ADLs were to a considerable extent attributable to impoverished health-related habits (e.g., smoking, alcohol use, unhealthy diet and physical inactivity).

An alternative approach would be to conceptualize disability as an independent variable for SOC. Although SOC is assumed to be steady throughout adulthood [4], a few long-term longitudinal studies have shown low test-retest stability beyond the fifth year of follow-up [23]. Volanen et al. [37] reported that SOC levels were not stable over time. Specifically, individuals exposed to negative life events had lower SOC scores in inverse proportion to the recency of the negative life event. Thus, it is possible that a strong pre-existing SOC could be tempered in the face of disability of an external cause. Conversely, sense of coherence may also be malleable by favorable experiences albeit this hypothesis has seldom been evaluated. For example, Graziano et al. [38] used SOC as an intervention outcome for a brief group-based cognitive behavioral intervention for adults with multiple sclerosis. The authors reported a modest change in SOC. The trend, however, was not statistically significant. Additional intervention and longitudinal studies are needed in order to ascertain the nature of the complex intersections between SOC, environmental factors, and disability observed in the current analysis. In addition, there is a limited number of studies evaluating the predictive role of SOC among the people older than 75. The validity of any hypotheses pertaining to the predictive or even causal roles of SOC over disability should be tested in the context of the ability of SOC to predict or cause disability should be evaluated across all phases of aging.

## Conclusion

In summary, while the present study does not provide straightforward evidence of a causal connection between SOC, disability and dependence, our results suggest that individuals with low SOC, poor access to social resources, and living in rural areas may constitute a high-risk group for disability among the elderly. Our findings are consistent with the view that SOC, and particularly its Meaningfulness and Comprehensibility components, are informative behavioral factors for the assessment, rehabilitation and service planning for the elderly. These findings expand our knowledge about the complex relations between SOC and disability among older and elderly population [38].

## Additional file

Additional file 1: Original Data Collection Questionnaires. (PDF 1507 kb)

## Abbreviations

ADL: Activities of daily living
BADL: Basic activities of daily living
CI: Confidence interval
IADL: Instrumental activities of daily living
ICF: International classification of disability
OR: Odds ratio
SOC: Sense of coherence
WHODAS 2.0: World Health Organization disability assessment schedule, 2^nd^ ed
