# Supplementary material for: A protective personal factor against disability and dependence in the elderly: an ordinal regression analysis with nine geographically-defined samples from Spain
Source: BMC Geriatr. 2017 Jan 31;17:42. doi: 10.1186/s12877-016-0409-9 (PMC5286674; doi:10.1186/s12877-016-0409-9)
Supplement: Additional file 1: — Original Data Collection Questionnaires. (PDF 1507 kb) [file 12877_2016_409_MOESM1_ESM.pdf]

Centro Nacional de Epidemiología

Estudio longitudinal del envejecimiento de poblaciones españolas

Cuaderno A

Examen Médico

Número de entrevista ☐☐☐ 00001

Código de provincia ☐☐ 00002

Índice

A. CONSENTIMIENTO INFORMADO 5

B. ANTECEDENTES PERSONALES 7

C. DATOS DE SALUD 8

I. Síntomas y molestias físicas 8

II. Estado mental geriátrico 10

II-1 Exploración Cognoscitiva 10

II-2 Exploración Psiquiátrica (GMS-B, HAS) 15

II-3 Consideraciones Finales 22

II-4 Ítems observación del GMS 23

II-5 Fiabilidad 26

II-6 Resumen Final 27

II-7 Diagnóstico Psiquiátrico 28

II-8 Diagnósticos GMS-HAS 28

III. Dolor 29

D. EXAMEN FÍSICO 32

E. CRIBAJE NEUROLÓGICO 34

I. Cuestionario de síntomas 34

II. Tareas físicas 35

F. EXAMEN NEUROLÓGICO 36

I. Exploración neurológica 36

II. Exploración neurológica: Parkinsonismo 39

G. MEDICACIÓN 40

H. RESUMEN DE LA HISTORIA CLÍNICA Y UTILIZACIÓN DE SERVICIOS 44

I. ESCALA DE IMPACTO FUNCIONAL DE ENFERMEDADES POR ORGANOS, APARATOS O SISTEMAS 50

J. RECAPITULACIÓN DE ACTUALIDAD: (DIAGNOSTICOS, ESTADO DE SALUD Y CRIBAJES) 53

A entregar a la persona entrevistada:

Estimado/a Sr. / Sra.:

En estas pocas líneas el Grupo de Estudio Epidemiológico del Envejecimiento en España, organizador del estudio longitudinal del envejecimiento de poblaciones españolas quisiera expresarle su agradecimiento. Su participación en este estudio resultará de gran valor para conocer y mejorar las condiciones de salud de las personas mayores en España.

En este estudio Vd. recibirá la visita de un médico que le entrevistará sobre distintos aspectos de su salud. El doctor se interesará tanto por su salud actual como por su salud anterior y le propondrá alguna exploración sencilla a realizar en el momento.

En una segunda entrevista, un profesional sanitario –médico o no- le pedirá que refiera las dificultades que sus problemas de salud le suponen para llevar a cabo las actividades de la vida diaria y como afecta su salud a su bienestar.

El primer entrevistador le entregará, si Vd. lo desea, una hoja que contiene una breve información acerca del objetivo y contenido de este estudio.

Sin más, esperamos que le resulten agradables estos encuentros y le agradecemos muy atentamente su colaboración.

Muchas gracias

Ministerio de Sanidad y Consumo

Centro Nacional de Epidemiología
Grupo de estudios de envejecimiento

INFORMACION A PARTICIPANTES EN ESTUDIO Y/O A FAMILIARES, ANTE COLABORACIÓN EN ESTUDIO DEL ENVEJECIMIENTO DE POBLACIONES ESPAÑOLAS

Una parte muy importante del estudio de la salud de poblaciones españolas lo constituye el estudio de la salud de los ancianos. Esto requiere el uso de métodos orientados a detectar la presencia de enfermedades crónicas, de discapacidad y también de la calidad de vida relacionada con la salud. El estudio en el que le proponemos participar es un estudio de envejecimiento que tiene como objetivo detectar la presencia de deterioro cognitivo o demencias en determinadas poblaciones españolas y poner a prueba la calidad de un cuestionario para estudio longitudinal del envejecimiento en nuestro país (comprensibilidad, duración del mismo, aceptabilidad, etc.). Para ello se realizaran, en el propio domicilio y en presencia de familiares o acompañantes si la situación lo requiere, dos entrevistas, una de ellas por médico y otra por personal sanitario. En estas entrevistas se determinará el estado de salud o la posible presencia de enfermedades sin diagnosticar utilizando cuestionarios, particularmente diseñados para conocer la discapacidad y calidad de vida y un breve examen clínico. También se deberán analizar informes o documentos médicos que obren en poder de la persona participante

La participación en este estudio no produce mas molestias que las de la dedicación de un tiempo a la realización de las entrevistas. Para las personas participantes este estudio puede permitir la identificación de enfermedades crónicas no diagnosticadas y tratables, por ejemplo la enfermedad de Parkinson o algunas demencias. También es posible que de dicho estudio no se derive ningún resultado concluyente o previamente no conocido acerca de la salud de la persona participante.

Toda la información obtenida se almacenará y tratará de forma confidencial, de acuerdo con las normas señaladas por la Agencia de Protección de datos y finalmente destruida. En el caso de que desee ser informado de los resultados individuales obtenidos en el estudio, estos serán comunicados - siguiendo sus instrucciones - a usted mismo, a su médico de familia o a sus familiares o representante legal

Nota. La información a enfermos requiere la integridad de sus facultades mentales, lo que suele ser poco frecuente para personas afectadas por demencia en cuyo caso la información se proporcionará a los familiares.

Estimado colega, este es el examen médico del estudio longitudinal del envejecimiento de poblaciones españolas diseñado por el Grupo Español de Estudios Epidemiológicos del Envejecimiento. Este cuadernillo está diseñado para que Vd. lea los enunciados en voz alta al participante; los mensajes tabulados en cursiva están dirigidos a Vd. y por tanto no deben ser leídos en voz alta. Por favor lea atentamente las instrucciones correspondientes a cada sección.

A. CONSENTIMIENTO INFORMADO

Código en caso de ausencia de respuesta:

Por favor complete cada ítem con el número de opción o respuesta apropiados. En caso de ausencia de respuesta utilice el código 99 seguido de alguna de las especificaciones siguientes.

1. *No puede responder por trastorno cognitivo*
2. *No puede hacerlo por limitaciones físicas*
3. *Se niega*
4. *No es apropiado*
5. *Desconoce la respuesta*
6. *Otro*

0. Identificación de la persona entrevistada:

Nombre :	______________________________	00001
Dirección postal:	______________________________	00002
Código postal:	☐☐☐☐☐	00003
Teléfono para contactos posteriores:	______________________________	00004
Fecha de la entrevista:	______________________________	00005

A continuación se presentan cinco ítems dirigidos a la obtención del consentimiento informado de la persona entrevistada. Es indispensable obtener la firma de la persona entrevistada, o en su caso del familiar o persona responsable. Por favor valore las condiciones del entrevistado y decida sobre la conveniencia de solicitar el consentimiento al inicio o al término de la administración de este cuadernillo.

He recibido información acerca del estudio longitudinal del envejecimiento de poblaciones españolas. En cualquier momento puedo interrumpir el examen, estudio o entrevista.

Estoy de acuerdo en:

1. Participar den el estudio longitudinal de envejecimiento de poblaciones españolas, que consta de dos entrevistas en las que se me realizarán preguntas y exámenes médicos relacionados con mi estado de salud.

1 Sí
2 No |__| 00102

2. Que el investigador pueda examinar mi historia clínica incluidas pruebas radiológicas, pruebas de laboratorio o cualquier información oportuna a este estudio y que permita que se desarrolle con la calidad más alta posible.

1 Sí
2 No |__| 00202

Médico de atención primaria o equivalente____

3. Que se contacte con el familiar o conocido detallado abajo para concertar la entrevista:

1 Sí
2 No |__| 00301

00302 **3.a** Nombre: ______________________________

00303 **3.b** Relación: ______________________________

00304 **3.c** Dirección: ______________________________

00305 **3.d** Número de teléfono: ______________________________

4. Yo (*el entrevistado*) he dado mi consentimiento personalmente.

1 Por escrito
2 De forma verbal |__| 00401

Firma ______________________________

5. El consentimiento ha sido proporcionado por un familiar o amigo:

1 Por escrito
2 De forma verbal |__| 00501

Firma ______________________________

Pedir a la persona entrevistada o a su familiar que facilite toda la documentación médica disponible. Tómese unos momentos para examinarla pues será necesaria durante la administración de este cuadernillo

A continuación voy a plantearle unas preguntas para ver como se encuentra. No se preocupe si a veces tiene la impresión de que le estoy pidiendo que me cuente cosas parecidas. En primer lugar le haré algunas preguntas sobre su salud y por último le examinaré

B. ANTECEDENTES PERSONALES

Código en caso de ausencia de respuesta:

Por favor complete cada ítem con el número de opción o respuesta apropiados. En caso de ausencia de respuesta utilice el código 99 seguido de alguna de las especificaciones siguientes.

1. *No puede responder por trastorno cognitivo*
2. *No puede hacerlo por limitaciones físicas*
3. *Se niega*
4. *No es apropiado*
5. *Desconoce la respuesta*
6. *Otro*

En primer lugar me gustaría que me hablara de los problemas de salud que haya tenido a lo largo de su vida:

Enfermedades físicas

____

____

____

____

____

Enfermedades mentales

____

____

____

____

____

Intervenciones quirúrgicas

____

____

____

____

____

Medicaciones previas y otros tratamientos

____

____

____

____

____

Otros aspectos

____

____

____

____

____

C. DATOS DE SALUD

I. Síntomas y molestias físicas

Código en caso de ausencia de respuesta:

Por favor complete cada ítem con el número de opción o respuesta apropiados. En caso de ausencia de respuesta utilice el código 99 seguido de alguna de las especificaciones siguientes.

1. *No puede responder por trastorno cognitivo*
2. *No puede hacerlo por limitaciones físicas*
3. *Se niega*
4. *No es apropiado*
5. *Desconoce la respuesta*
6. *Otro*

8. ¿Podría indicarme si ha padecido alguno de los siguientes síntomas en los últimos tres meses?

Indicar las opciones que procedan; son posibles distintas alternativas. Compruebe la presencia de los síntomas haciendo uso de la siguiente lista.

Sección A

1	Mal apetito	|__|	00801
2	Adelgazamiento	|__|	00802
3	Sobrepeso	|__|	00803
4	Nauseas	|__|	00804
5	Sequedad de boca	|__|	00805
6	Dolor de vientre	|__|	00806
7	Diarrea	|__|	00807
8	Estreñimiento	|__|	00808

Sección B

1	Dificultades para orinar	|__|	00809
2	Incontinencia	|__|	00810

Sección C

1	Dolor de cabeza	|__|	00811
2	Vértigo	|__|	00812
3	Sudoración	|__|	00813
4	Escalofrío, sensación de frío	|__|	00814
5	Falta de aire, dificultad respiratoria	|__|	00815
6	Tos	|__|	00816

Sección D

1	Perdida de audición	|__|	00817
2	Perdida de visión o molestias oculares	|__|	00818

Sección E

1	Dolor en la espalda	|__|	00819
2	Dolor en el pecho	|__|	00820
3	Dolor en las piernas	|__|	00821
4	Molestias en las articulaciones	|__|	00822
5	Dificultades para caminar	|__|	00823

Sección F

1	Fatiga	|__|	00824
2	Dificultades para dormir	|__|	00825
3	Dificultades para concentrarse	|__|	00826
4	Dificultades para relajarse	|__|	00827
5	Agotamiento	|__|	00828
6	Inquietud motora	|__|	00829

Por favor anote el número de minutos invertido en la administración de esta sección |______| 00901

II. Estado mental geriátrico

Muchas gracias. A continuación voy a preguntarle con mas detalles sobre su salud y sobre cómo se encuentra

II-1 Exploración Cognoscitiva

I-1.1 Memoria

Código en caso de ausencia de respuesta:

Por favor complete cada ítem con el número de opción o respuesta apropiados. En caso de ausencia de respuesta utilice el código 99 seguido de alguna de las especificaciones siguientes.

1. *No puede responder por trastorno cognitivo*
2. *No puede hacerlo por limitaciones físicas*
3. *Se niega*
4. *No es apropiado*
5. *Desconoce la respuesta*
6. *Otro*

Para empezar, le voy a decir mi apellido y me gustaría que usted lo recordara.

Repetir lentamente el apellido hasta su correcta repetición. Se permiten tres intentos. Se admiten fallos menores de pronunciación

10. Mi apellido es ... ¿puede repetírmelo?

1 Repetición correcta
2 No puede repetirlo |__| 01001

Fijándose en la información reflejada en los documentos médicos de la persona entrevistada, realice la siguiente pregunta repitiendo los datos, por ejemplo:

Veo que tiene/ha tenido algún problema/s de ..., etc. Por todos ellos le voy a ir preguntando. Veo que también ha tenido problemas de ...

11. *En caso positivo,* ¿le ha afectado la memoria?
En caso negativo, veo que recuerda bien los datos, ¿conserva bien la memoria?

1 Sí
2 No |__| 01101

12. *Si responde que tiene problemas:* ¿supone ello un problema para Vd.?

1 Sí
2 No |__| 01201

13. ¿Ha tenido recientemente tendencia a olvidar cosas? (¿De qué tipo de cosas se olvida Vd.?) (¿De los nombres de sus familiares o amigos próximos?) (¿De dónde ha dejado las cosas?)

En caso negativo pasar a la pregunta 15

1 No olvidos
2 Olvida los nombres de familiares o amigos, o los confunde (no se incluyen errores pasajeros)
3 Olvida dónde ha dejado las cosas
4 Olvida ambas cosas |__| 01301

14. Aproximadamente, ¿cuándo comenzó a notarlo?:

1 Ocurrió por primera vez durante los últimos 1-2 años
2 Ocurrió por primera vez durante los últimos 3-4 años
3 Ocurrió por primera vez durante los últimos 5-10 años
4 Ocurrió por primera vez hace más de 10 años |__| 01401

15. ¿Recuerda mi apellido, se lo dije al principio?, ¿cuál es?

1 Lo recuerda perfectamente
2 No recuerda correctamente el nombre del entrevistador
(*se permiten errores mínimos de pronunciación*) |__| 01501

16. Por cierto, ¿me conocía a mí anteriormente, me ha visto Vd. antes? Si no está seguro ¿me ha visto en la última semana? En caso afirmativo: ¿cuándo? ¿dónde? ¿cómo fue? ¿qué estaba haciendo? ¿qué llevaba puesto? ¿qué hicimos juntos? ¿qué le dije?

1 No confabula
2 Da una respuesta positiva y una explicación sencilla dentro de los límites de sus posibilidades, pero el entrevistador sabe que es incorrecto, p. ej.: "Vd. fue el doctor que me examinó ayer".
3 Confabulación |__| 01601

1. *Observación:* Habla divagando, pero las ideas están razonablemente bien formadas

1 Sí
2 No |__| 01701

2. Ahora tengo que hacerle una pregunta sencilla, ¿cómo se llama el Presidente del Gobierno?

1 Bien
2 No recuerda el nombre del Presidente del Gobierno |__| 01801

Si lo dice mal decir: Bien, en realidad se llama...

3. ¿Quién fue el anterior Presidente del Gobierno?

1 Bien
2 No recuerda el nombre del anterior Presidente del Gobierno |__| 01901

4. *Observación: En opinión del entrevistador, el entrevistado tiene dificultades con su memoria*

1 Sí
2 No |__| 02001

I-1.2 Orientación

21. También hablando de la memoria: ¿Recuerda en qué año nació?

1 Lo recuerda
2 El/ella no lo sabe decir
3 Incorrecto o irrelevante
4 Sin respuesta
5 Anotar el año de nacimiento dado __________ 02101 |__| 02101

22. ¿Qué edad tiene?

1 Correcto
2 El/ella no lo sabe decir
3 Incorrecto o irrelevante
4 Sin respuesta
5 Anotar la edad dada __________ 02201 |__| 02202

Si no existen discrepancias entre la edad y la fecha de nacimiento pasar a la pregunta 24

23. No acaban de salirme bien las cuentas al sumar sus años, ¿puede Vd. ayudarme?

1 No comete error
2 Muestra una marcada inseguridad acerca de sus años/edad y su fecha de nacimiento
3 El entrevistado no corrige la discrepancia entre la fecha de nacimiento que ha declarado y su edad (ignorar un error de un solo año)
4 Error de 2 ó 3 años
5 Error de más de 3 años |__| 02301

24. A veces, cuando uno no está del todo bien, puede tener dificultades para recordar la fecha. ¿Puede decirme qué día del mes es hoy?, ¿qué día de la semana?, ¿qué mes?, ¿qué estación?, ¿qué época del año?, ¿qué año?

Anotar la fecha indicada (incluye día de la semana, día del mes, mes, estación y año).

____ 02401

Se valorarán estos ítems en el ECMM más adelante
Si recuerda perfectamente la fecha, pasar a la pregunta 25

1 Correcto
2 Error en un solo día/mes/año
3 Error en más de un día/mes (se permite, p. ej., marzo en la 1ª semana de abril)/año
4 Error en día de la semana
5 Error en el mes
6 Él/ella no sabe el mes
7 Al menos parte de la respuesta es incompleta, irrelevante o no hay respuesta
8 Error en el año
9 Él/ella no sabe el año |__| 02402

Si es entrevistado en un lugar distinto a su propia casa, pasar a la pregunta 26

25. Sabe cuál es su dirección postal (para las cartas, correo)?

Hacer más preguntas para ver la dirección completa y asegurarse que el entrevistado sabe o no los cinco ítems del ECMM. Si se le entrevista en casa, pasar al ECMM

____ 02501

26. ¿Cómo se llama este lugar donde estamos ahora?

Hacer más preguntas para ver la dirección completa y asegurarse que el entrevistado sabe o no los cinco ítems del ECMM

____ 02601

I-1.3 Examen Cognoscitivo Mini-Mental (MEC)

Por favor puntúe el ECMM una vez concluida la pasación y anote el resultado en el cuadernillo para su consideración durante la administración de esta batería

28. Orientación Temporal

No preguntar, puntuar a partir de las respuestas correspondientes de la sección de orientación del GMS (secc. I-1.2, ítems 21-27)

1 Día
2 Fecha
3 Mes
4 Estación
5 Año |__| 02802
Puntuación _______ 02801

29. Orientación Espacial

No preguntar, puntuar a partir de las respuestas correspondientes de la sección de orientación GMS (secc. I-1.2, ítems 21-27)

1 Calle/Hospital
2 Nº calle/Planta
3 Ciudad
4 Provincia
5 Nación |__| 02901

30. Ahora, por favor, le voy a pedir que repita estas 3 palabras:

1 Pelota
2 Caballo
3 Manzana |__| 03001

Repetirlas hasta que las aprenda y contar los intentos _______ 03002

Acuérdese de ellas, porque se las preguntaré dentro de un rato.

31. *Atención y cálculo:* Si tiene 30 monedas y me va dando de 3 en 3. ¿Cuántas le van quedando?

1 27
2 24
3 21
4 18
5 15 |__| 03102
Puntuación _______ 03101

32. *Memoria:* ¿Recuerda las 3 palabras que le he dicho antes?

1 Pelota
2 Caballo
3 Manzana |__| 03201

33. *Denominación:* Mostrar un bolígrafo: ¿Qué es esto?

1 Sí
2 No |__| 03301

34. *Denominación:* Repetirlo con el reloj: ¿Qué es esto?

1 Sí
2 No |__| 03401

35. *Repetición:* Repita esta frase: "En un trigal había cinco perros"

1 Sí
2 No |__| 03501

36. *Comprensión:* Coja este papel con la mano derecha, (1) dóblelo por la mitad (2) y póngalo en el suelo/mesa (3)

1 2 3 Sí
1 2 3 No |__| 03601

37. *Lectura:* Lea esto y haga lo que dice:

CIERRE LOS OJOS

1 Sí
2 No |__| 03701

38. *Escritura:* Escriba una frase (algo que tenga sentido):

1 Sí
2 No |__| 03801

39. *Dibujo:* Copie este dibujo:

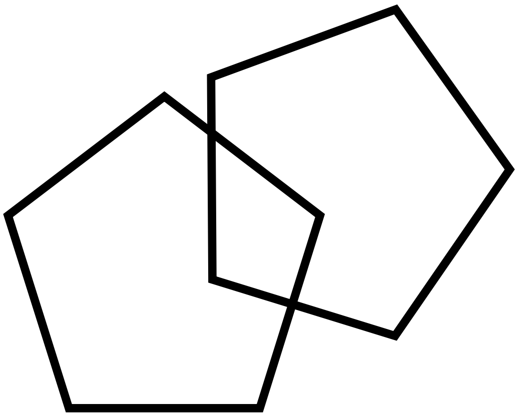

1 Sí |__| 03901
2 No

PUNTUACIÓN TOTAL

PUNTUACIÓN TOTAL CORREGIDA

Por favor, obtenga y anota la puntuación total y corregida del MMCE en el espacio correspondiente

40. Nivel de consciencia

1 Alerta
2 Obnubilación
3 Estupor
4 Coma |__| 04001

41. *Juicio:* Competencia del entrevistado para completar el ECMM

1 No hubo problemas en la administración
2 El entrevistado es analfabeto
3 El entrevistado tiene algún problema físico que le impide el cumplimiento del ECMM
4 El entrevistado está demasiado demenciado como para completar el ECMM
5 Circunstancias sociales impiden la administración
6 Se interrumpe la entrevista sin poder completar el test |__| 04101

42. *Número de ítems no contestados del ECMM* ___________ 04201

43. ¿Es Vd. diestro o zurdo? ¿Con qué mano corta el pan (etc.) o tiraría una pelota?

1 Diestro
2 Zurdo |__| 04301

Por favor, anote el tiempo transcurrido de la entrevista hasta este momento |______| 04401

II-2 Exploración Psiquiátrica (GMS-B, HAS)

II-2.1 Preocupación

Código en caso de ausencia de respuesta:

Por favor complete cada ítem con el número de opción o respuesta apropiados. En caso de ausencia de respuesta utilice el código 99 seguido de alguna de las especificaciones siguientes.

1. *No puede responder por trastorno cognitivo*
2. *No puede hacerlo por limitaciones físicas*
3. *Se niega*
4. *No es apropiado*
5. *Desconoce la respuesta*
6. *Otra*

45. Ahora, si no le importa, me gustaría preguntarle por el último mes, ¿cómo se ha sentido durante el último mes? Por ejemplo: ¿Le preocupa alguna cosa?

Si no menciona ninguna preocupación, pasar a la pregunta 50

____ 04501

46. ¿De qué se preocupa Vd.? Tema o contenido de las preocupaciones, si lo hay.

1 Su propia salud
2 La salud de otros
3 Sus negocios (incluyendo la pensión)
4 Problemas familiares (aparte de la salud) |__| 04601

47. Si tiene hijos: ¿Y, en concreto, hay algo en la relación con sus hijos que le preocupe o le altere? ¿Qué es?

1 Alterado o preocupado por las relaciones con sus hijos |__| 04701

48. ¿Cuánto se preocupa Vd.?

1 Se preocupa mucho (p. ej. de una o dos cosas)
2 Es preocupadizo, se preocupa de casi todo ahora |__| 04801

49. ¿Es desagradable preocuparse tanto?, (¿Puede Vd. dejar de preocuparse?, ¿Le vienen estos pensamientos constantemente a la cabeza?)

1 Preocupación desagradable que le viene constantemente a la cabeza o

que no puede detener |__| 04901

II-2.2 Ansiedad General

50. ¿Se asusta Vd., se pone nervioso? (¿Le ocurre esto últimamente?) (¿Qué le hace sentirse así?)

1 Hay ansiedad o miedo subjetivos, no proporcionales al suceso que los ocasiona

|__| 05001

51. ¿Ha tenido Vd. como crisis o ataques de miedo, (incluso de pánico,) en los que se haya visto obligado a hacer algo en concreto para detenerlos?

1 Episodio crítico de ansiedad que la persona intenta detener p. ej. pidiendo ayuda, acudiendo al médico/urgencias, saliendo corriendo de la habitación |__| 05101

Si no hay crisis de pánico, pasar a la pregunta 52

En caso afirmativo determinar el número de crisis de pánico:

51.a En la última semana __________ 05102

51.b En el último mes __________ 05103

51.c En el último año __________ 05104

II-2.3 Tensión

52. ¿Está cansado? (¿Exhausto?) Si responde: No ¿Y por la noche?

1 Está cansado o exhausto por el día o por la noche |__| 05201

53. ¿Tiene dificultades para relajarse, para relajar su cuerpo?

1 Dificultades para descansar (relajarse) |__| 05301

54. ¿Tiene dolores de cabeza? ¿En qué parte? ¿Cómo son?

1 No
2 Refiere dolores de cabeza
3 Refiere cefaléas tensionales, p. ej.: "bandas alrededor de la cabeza", "presión", " tensión en la nuca", "pesadez" |__| 05401

Como dolores tensionales no anotar las migrañas, p. ej., dolor de cabeza unilateral, pulsátil, con distorsión visual y náusea.

II-2.4 Irritabilidad

55. ¿Está Vd. últimamente más irritable (enfadado)?, ¿y le ocurre a menudo?, (¿incluso cada día?), (¿Durante cuánto tiempo?)

1 Admite su irritabilidad (enfado)
2 Irritable casi todos los días durante al menos dos semanas en el último mes |__| 05501

56. Y con Vd. mismo, ¿Se enfada con Vd. mismo?

1 Se enfada consigo mismo |__| 05601

II-2.5 Síntomas Neurovegetativos

57. ¿Ha notado los latidos de su corazón (palpitaciones) o se ha encontrado tembloroso en el último mes? (¿cuando esto no era debido al ejercicio?) ¿Qué sucedió?, ¿Ha tenido otros síntomas como, por ejemplo: sequedad de boca, dificultades para respirar, sensación de ahogo, malestar, dolor en el pecho, náuseas, problemas en el vientre, mareos, escalofríos, etc.?

1 Palpitaciones (p. ej., el paciente es consciente de los latidos de su corazón, no debido al ejercicio pero a menudo acompañado de ansiedad)
2 Temblores ligeros o de grado medio debidos a la ansiedad
3 Otros signos corporales de ansiedad; especificar ______________________________ 05701

|__| 05702

II-2.6 Trastornos Somáticos

58. ¿Qué tal apetito tiene? ¿Come bien? (¿Ha comido últimamente más o menos de lo normal?)

1 Apetito normal
2 Disminución de la apetencia por la comida
3 Aumento de la apetencia por la comida |__| 05801

Si come bien, pasar a la pregunta 60

59. ¿A qué se debe? ¿Lleva pasándole esto casi todos los días de este último mes? Mal apetito sin alteración médica conocida y sin náuseas o dispepsia

1 Alteración del apetito presente casi todos días durante al menos 2 semanas en el último mes

|__| 05901

59.a *En caso positivo especificar*

1 Disminución del apetito
2 Aumento del apetito |__| 05902

60. ¿Ha perdido peso en los últimos 3 meses? (¿Ha ganado peso?)¿Cuánto? ¿Y en el último mes?

1 No
2 Ha perdido 4.5 Kg. o más durante los últimos 3 meses
3 Ha perdido 4.5 Kg. o más durante el último mes
4 Ha ganado 4.5 Kg. o más durante el último mes |__| 06001

61. ¿Ha tenido problemas para dormir últimamente? (¿Ha tomado algo para dormir?) ¿Durante cuánto tiempo lleva ocurriéndole? ¿Qué suele pasar?

1 Problemas o cambio reciente en el patrón de sueño |__| 06101

En caso negativo pasar a la pregunta 66

61.a En caso afirmativo especificar el tipo de alteración

1 Padece insomnio la mayor parte de la noche y duerme principalmente durante el día
2 Marcado insomnio la mayor parte de las noches, durante al menos 2 semanas en el último mes
3 Marcado y excesivo sueño la mayor parte de las noches, durante al menos dos semanas en el último mes

|__| 06102

62. Sólo si no ha quedado claro, insistir: Perdone que insista un poco. En concreto ¿Ha tenido dificultades para conciliar el sueño, para quedarse dormido? ¿Permanece despierto mucho tiempo?

1 Dificultad para conciliar el sueño. Si toma hipnóticos, valorar lo que el entrevistado cree que ocurriría si no los tomara

|__| 06201

63. ¿Se despierta por la noche?

1 Interrupción del sueño por la noche |__| 06301

Se incluye cuando el entrevistado se despierta tras un sueño ligero y no puede dormirse de nuevo durante más tiempo. Si toma hipnóticos, valorar lo que el entrevistado cree que ocurriría si no los tomara

64. Finalmente, ¿Se despierta temprano por las mañanas, demasiado temprano, sin poder volverse a dormir? ¿A qué hora ocurre esto? ¿Cuál es su hora normal de levantarse? ¿Cuántas veces le ha ocurrido en las últimas semanas?

1 Despierta sobre 2 horas o más antes de la hora normal de despertarse y no puede volver a dormirse casi todas las noches durante al menos 2 semanas en el último mes

|__| 06401

65. ¿Por qué se despierta? (¿Cuál es la dificultad?) ¿Es un problema (físico), del tipo de tener que ir al baño, o por algún dolor? ¿Le molesta el ruido?

1 Principalmente se debe o procede de una causa física corporal, o ruidos, etc
2 La dificultad se debe a pensamientos o ánimos alterados, o a la tensión emocional (p. ej., permanece despierto con sentimientos o pensamientos depresivos-ansiosos)

|__| 06501

II-2.7 Soledad

66. ¿Se siente Vd. sólo, tiene soledad?

1 Admite sentirse solo, en soledad (subjetivo) |__| 06601

Si no se siente solo pasar a la pregunta 69

67. Esta soledad, ¿le preocupa, le afecta mucho? ¿La puede evitar?

1 Se siente solo, en soledad (subjetivo) y no puede hacer nada por evitarlo
2 Preocupado o deprimido por su actual soledad |__| 06701

68. Y para el futuro, ¿Le preocupa la posibilidad (perspectiva) de estar (vivir) solo (sin otras personas) en el futuro?

1 Le preocupa estar solo, sin otras personas (objetivo), en el futuro |__| 06801

II-2.8 Depresión

69. ¿Qué me dice de su estado de ánimo?, (¿Ha estado Vd. bajo de ánimos, incluso triste [deprimido, infeliz] últimamente?)

1 Estado de ánimo deprimido |__| 06901

70. ¿Ha llegado a llorar últimamente? (¿Con qué frecuencia?)

1 Ha llorado |__| 07001

Si ha llorado, pasar a la pregunta 73

71. ¿Ha tenido ganas de llorar sin llegar a hacerlo? (¿Con qué frecuencia?)

1 Se ha sentido con ganas de llorar |__| 07101

Si el entrevistado no admite estar deprimido, haber llorado o haber querido llorar, Insistir:

72. Se suele considerar que las personas de edad a menudo están bajos de ánimo, con tendencia a estar tristes pero, a pesar de ello, Vd. se encuentra bien, ¿Es así?

1 Sí
2 No |__| 07201

Si no existen indicios de depresión, pasar a la pregunta 83

73. ¿Está Vd. así todo el tiempo? ¿Cuánto le ha durado ese estado de ánimo, esa depresión? (¿Sólo unas pocas horas o más?)

1 La depresión, llanto o ganas de llorar dura más de unas pocas horas ocasionales
2 La depresión, llanto o ganas de llorar está presente la mayor parte del tiempo

|__| 07301

73.a ¿Durante cuanto tiempo la ha tenido?

1 Presente durante al menos dos semanas seguidas en el último mes |__| 07302

74. ¿Varía su estado de ánimo mucho en el mismo día, es cambiante, fluctúa mucho?

1 Humor fluctuante |__| 07401

75. ¿A qué hora del día se siente peor?

Si en ninguno de los períodos siguientes, puntuar: 0. Si es severa durante todo el día, puntuar 1 en los 3 ítems.

1 Sobre todo por la mañana
2 Sobre todo por la tarde
3 Sobre todo por la noche |__| 07501

76. ¿Hay algo que alivie su depresión?,¿las visitas, las noticias agradables, las distracciones? ¿Durante cuánto tiempo se alivia?

1 Alguna experiencia placentera alivia la depresión, pero las visitas o actividades de entretenimiento no la alivian o sólo la alivian 1 ó 2 horas
2 Anhedonia profunda: nada alivia la depresión |__| 07601

II-2.9 Culpabilidad

77. ¿Tiende Vd. a reprocharse algo, a sentirse poco útil o incluso a sentirse culpable? Sólo si sugiere que sí, continuar: ¿De qué? (¿Quiere decir que de hecho se siente inútil?) (¿Cuánto tiempo lleva sintiéndose así?) (¿Cree Vd. que es razonable sentirse así?)

1 Excesiva autoinculpación obvia por "pecadillos" pasados o del presente (No se incluye una ligera o justificada autoinculpación)
2 Menciona arrepentimientos del pasado que pudieran o no ser justificables
3 Se siente inútil o con una severa culpa casi todos los días durante al menos dos semanas
4 Inutilidad o culpa de intensidad delirante la mayoría de los días

|__| 07701

78. Veo que ha estado bajo de ánimos, (resumir los síntomas observados), ¿Cómo ve (o siente) su futuro? (¿Cómo cree que le saldrán las cosas en el futuro?) (¿Qué espera del futuro?)

1 No es pesimista, pero no tiene expectativas (vive día a día, "a mi edad no hay futuro") (profundizar en el tema)

|__| 07801

78.a *Si es pesimista:* ¿Por qué? ¿Se ha sentido realmente desesperado (sin esperanzas)?
Si se evade: ¿Hay algo en su futuro en lo que Vd. no quiera pensar?

1 Es pesimista, el futuro le parece negro, no ve futuro o su futuro le es inaguantable
2 Sensación general de desesperación
3 Pesimismo obviamente justificado por las circunstancias |__| 07802

79. ¿Ha llegado a sentir que la vida no merece ser vivida?

1 Lo ha sentido |__| 07901

Si no lo ha sentido, pasar a la pregunta 83

80. ¿Ha sentido Vd. alguna vez que preferiría estar muerto? (Porque la vida ha llegado a ser una carga para Vd.) ¿Ha querido alguna vez terminar con todo? (¿Ha pensado hacer algo al respecto Vd. mismo?) (¿Suicidarse?)

1 Ha tenido pensamientos suicidas o ha deseado estar muerto |__| 08001

Si nunca los ha sentido, pasar a la pregunta 83

81. ¿Cuándo ocurrió esto? ¿Últimamente? (¿En el último mes?) (¿Cuántas veces?)

1 Nunca
2 Alguna vez en el último mes
3 En el último año |__| 08101

81.a Los deseos de estar muerto...

1 Han durado al menos dos semanas en el último mes |__| 08102

Si no lo ha sentido en el último mes, pasar a la pregunta 83

82. ¿Llegó a intentar algo al respecto, hacerse daño hace poco tiempo? ¿Cuándo? ¿Qué hizo? (¿O qué planeó hacer?) ¿Por qué piensa que se sintió así?

1 Ha rechazado el suicidio, pero ha deseado estar muerto, porque la vida es una carga.
2 Ha hecho o planeado algo para suicidarse |__| 08201

83. *Observación:* Parece (al verlo o escucharlo) tenso, preocupado, deprimido o miedoso

Si no presenta estos síntomas, pasar a la pregunta 84

1 Parece tenso o preocupado
2 Se le escucha triste, apagado, pesimista o deprimido
3 Parece aprensivo o miedoso
4 Ojos húmedos: llenos de lágrimas o llorando |__| 08301

II-2.10 Lentitud

84. ¿Se ve Vd. (está) lento en sus movimientos (físicos)?

1 Lentitud subjetiva de movimientos |__| 08401

85. ¿Se ve Vd. con muy poca energía (para hacer las cosas que quiere hacer)? ¿Cuánto tiempo lleva ocurriéndole? ¿Es Vd. así la mayor parte del tiempo?

1 Restricción subjetiva de energía
2 Presente casi todos los días durante al menos dos semanas |__| 08501

86. ¿Puede hacer sus tareas o su actividad habitual? (Valorar sólo las restricciones no impuestas por el ambiente)

1 Hace menos de lo normal |__| 08601

Si no hay enlentecimiento, falta de energía o reducción de actividad, pasar a la pregunta de observación 92

87. ¿Cuándo comenzó este problema (el enlentecimiento, la pérdida de energía, la reducción de actividad)? ¿Le ha ido a peor en los últimos meses?

1 Comenzó o empeoró en los últimos 3 meses |__| 08701

88. ¿Se siente peor en algún momento del día?

1 No hay diferencias
2 Lentitud o falta de energía peor por la mañana
3 La lentitud o falta de energía aumenta en las últimas horas de la tarde |__| 08801

89. ¿Está presente casi todos los días?

1 La lentitud ha estado presente casi todos los días durante al menos dos semanas |__| 08901

90. ¿Qué pasa cuando alguien le visita o Vd. tiene que salir? ¿Se cansa demasiado?

1 No soporta incluso estas actividades normalmente placenteras |__| 09001

91. ¿Permanece Vd. ahora mucho tiempo sentado (o más tiempo en la cama de lo normal) por falta de energía?

1 Está sentado o en la cama por falta de energía |__| 09101

92. *Observación:*

1 Muy lento en todos sus movimientos |__| 09201

II-2.11 Interés

93. ¿Se interesa por las cosas? (¿Mantiene sus intereses?)

1 Tiene menos interés por las cosas de lo que es normal para él/ella |__| 09301

94. ¿Disfruta Vd. con algo?, ¿Qué le gusta hacer últimamente? (¿Ha habido algún cambio?)

1 Prácticamente no disfruta con nada |__| 09401

Si no ha descendido su interés, pasar a la pregunta 98

95. ¿Cuándo notó Vd. esta pérdida de interés (que no disfruta)? ¿Cuándo comenzó? ¿Ha aparecido recientemente? ¿Durante cuánto tiempo?

1 Perdida de interés/disfrute gradual durante algunos años
2 La pérdida de interés/disfrute ha ocurrido sólo en los últimos 3 meses |__| 09501

96. ¿Le pasa casi todos los días?

1 Sin interés/disfrute casi todos los días durante al menos 2 semanas el último mes |__| 09601

97. Y, ¿a qué se debe?, ¿lo sabe?, considera que se debe a estar deprimido o nervioso?

2 Demasiado deprimido o nervioso |__| 09701

II-2.12 Concentración

98. ¿Cómo está de concentración? *Sólo sondear.* ¿Puede Vd. concentrarse, por ejemplo, en un programa de T.V. (radio, película)? (¿Puede Vd. verlo-escucharlo entero?) (¿Le ocurre a menudo?)

1 Dificultad de concentración para el entretenimiento |__| 09801

99. ¿Lee Vd.? ¿Puede concentrarse en lo que lee? (¿Le ocurre a menudo?)

1 Dificultad de concentración en la lectura |__| 09901

Si no existen dificultades, pasar a la pregunta 111

100. ¿Cuánto tiempo lleva esto molestándole?

1 Dificultad de concentración en cualquiera de sus formas casi todos los días durante al menos dos semanas

|__| 10001

101. *Observación:*

1 Dificultad obvia de concentración en la entrevista |__| 10101

II-2.13 Dificultades de pensamiento

102. *Observación:*

1 Parece confuso (se le mezclan los pensamientos, no puede pensar claramente)
2 Parece indeciso |__| 10201

II-2.14 Persecución

103. ¿Cómo se lleva con la gente normalmente? ¿Le hacen sentirse mal fácilmente?

1 Se siente mal fácilmente |__| 10301

104. A veces nos dicen las personas (de edad) que alguna vez sienten que la gente está hablando mal de ellos, ¿le ha ocurrido a Vd.? Si sugiere que sí, preguntar: ¿Ha llegado a pensar que se ríen de Vd.?

1 Ha tenido esa sensación |__| 10401

Si la anterior pregunta puntúa 0, pasar a pregunta 106

105. ¿Cree que realmente es cierto o es quizás una forma de pensar suya? (¿Está Vd. seguro?)

1 Probablemente no es verdad
2 Considera que es cierto
3 Está convencido de que es cierto |__| 10501

106. No esperamos que se trate con todo el mundo pero ¿Hay alguien especial -no necesita decirme quién- con quien tenga particular dificultad para relacionarse?

1 Hay alguna/varias personas |__| 10601

107. *Observación:*

1 Parece indebidamente suspicaz |__| 10701

II-2.15 Distorsión de la Percepción

108. ¿Le está ocurriendo últimamente alguna cosa rara o extraña, algo que no puede explicar? ¿Me puede poner algún ejemplo?. Yo le ayudo, por ejemplo, ¿está sintiendo, viendo, oyendo, etc. algo raro?

1 Cree que le sucede algo extraño |__| 10801

108.a Especificar tipo de alucinaciones:____ 10802

109. Juicio:

109.a Tiene otros delirios. Especificar: ____ 10901

109.b Tiene otras alucinaciones. Especificar: ____ 10902

109.c Tiene otros síntomas psicóticos. Especificar: ____ 10903

II-3 Consideraciones Finales

Código en caso de ausencia de respuesta:

Por favor complete cada ítem con el número de opción o respuesta apropiados. En caso de ausencia de respuesta utilice el código 99 seguido de alguna de las especificaciones siguientes.

1. *No puede responder por trastorno cognitivo*
2. *No puede hacerlo por limitaciones físicas*
3. *Se niega*
4. *No es apropiado*
5. *Desconoce la respuesta*
6. *Otro*

110. ¿Le ha ocurrido alguna otra cosa durante este último mes que yo no le haya preguntado o incluido en esta entrevista?

Anotar aquí cualquier punto que parezca ser importante o no usual, referente a la entrevista, no incluido en el programa. Valorar siempre que sea posible toda información contemplada aquí en su ítem adecuado. Por favor, usar esta sección para describir completamente la psicopatología no valorada adecuadamente en el programa, o cuando se dude de su adecuada o apropiada valoración.

Atención, esta sección deberá leerse, UTILICE LETRAS MAYÚSCULAS.

____

____

____

____

____

____

____

____

____ 11001

111. *En caso de posible "diagnóstico" (dirigir está pregunta al familiar o cuidador principal cuando sea necesario):* ¿Le gustaría que le visitase un médico en su casa?

1 Sí
2 No |__| 11101

II-4 Ítems de observación del GMS

II-4.1 Puntajes Conductuales

Código en caso de ausencia de respuesta:

Por favor complete cada ítem con el número de opción o respuesta apropiados. En caso de ausencia de respuesta utilice el código 99 seguido de alguna de las especificaciones siguientes.

1. No puede responder por trastorno cognitivo
2. No puede hacerlo por limitaciones físicas
3. Se niega
4. No es apropiado
5. Desconoce la respuesta
6. Otro

Valorar los ítems siguientes sólo si la entrevista no ha sido completada *(1. Si; 2. No)*

113. Depresión: Parece triste o afligido o deprimido |__| 11301

114. Depresión: Ojos húmedos: llenos de lágrimas o llorando |__| 11401

115. Muy lento en todos sus movimientos |__| 11501

116. Dificultades de pensamiento: Parece confuso |__| 11601

117. Dificultades de pensamiento: Parece indeciso |__| 11701

118. Incomodidad social: Parece indebidamente suspicaz |__| 11801

119. Concentración: Dificultad clara para concentrarse en la entrevista |__| 11901

120. Expresión emocional disminuida:

120.b Voz monótona. No se muestra expresivo en la conversación |__| 12001

120.c No hay gestos acompañantes al habla |__| 12002

120.d No se muestra una emoción apropiada cuando se comentan ideas normales o delirantes las cuales normalmente producirían algún tipo de emoción |__| 12003

120.e Estado de ánimo uniformemente aplanado, independientemente del tono de la conversación (indiferencia, aceptación apática) |__| 12004

121. Expresión emocional disminuida:

121.a Pequeños accesos incontrolables de llanto |__| 12101

121.b Pequeños accesos incontrolables de risa |__| 12102

122. Risa

122.a Exaltado, eufórico, aunque quizás esté cambiando hacia irritabilidad o depresión |__| 12201

122.b Alegría contagiosa |__| 12202

123. Descarado: bromas estúpidas, comentarios poco serios |__| 12301

124. Poco cooperador etc.: Intenta comenzar una riña o discusión |__| 12401

125. Abandono personal:

125.a Las ropas están sucias, desarregladas o huelen mal |__| 12501

125.b Pelo despeinado, enredado o enmarañado |__| 12502

125.c Sin afeitar, con mocos en la nariz, cara sucia, etc |__| 12503

126. Conducta: Inquieto:

126.a Se mantiene sin estar quieto o torciéndose en el asiento |__| 12601

126.b Se levanta y se mueve alrededor sin descanso |__| 12602

127. Movimientos especiales:

127.a Movimientos coreiformes (movimientos involuntarios de la cabeza, cuerpo o miembros en reposo continuos, sin propósito, espasmódicos) |__| 12701

127.b Movimientos atetósicos (movimientos de la lengua, mandíbulas o miembros continuos, sin propósito, lentos, retorcidos) |__| 12702

127.c Movimientos parkinsonianos (temblor de las manos repetido y regular característico en reposo. Descrito como "contar monedas", como si el entrevistado estuviera contando monedas entre el pulgar y la punta de los dedos). |__| 12703

128. Raro, extravagante:

128.a Movimientos estilizados, algo más que balancearse en la silla o balancear los pies |__| 12801

128.b Movimientos repetitivos estereotipados, p. ej.: moverse en la silla, frotando cosas, etc. |__| 12802

128.c Toma posturas extrañas sin ninguna razón aparente |__| 12803

129. Pérdida de la compostura social normal:

129.a Deliberadamente rasga, arroja o rompe algo |__| 12901

129.b Se toca los genitales, mete el dedo en la nariz, eructa, etc. |__| 12902

129.c Se desnuda o exhibe sus genitales |__| 12903

129.d Hace sugerencias sexuales o insinuaciones al entrevistador |__| 12904

129.e Palabras o gestos obscenos |__| 12905

130. Habla: Restricción de la cantidad:

130.a El paciente está totalmente mudo, pronuncia no más de media docena de palabras reconocibles en respuestas a las preguntas en el curso de la entrevista. No incluir los comentarios en alto que se hace a sí mismo |__| 13001

130.b El paciente frecuentemente deja de contestar. Hay que repetir las preguntas |__| 13002

No puntuarlo si las respuestas del enfermo son inaudibles o incomprensibles

131. Habla: Incoherencia:

131.a Calidad vaga y amorfa del habla. La expresión de pensamientos o ideas no es completa; los temas cambian continuamente o se funden unos con otros. Las ideas comunicadas no son claras |__| 13101

131.b Irrelevante. El contenido de las respuestas a veces tiene poca relación, si tiene alguna, con lo que se le ha preguntado (no incluir el habla cambiante de un tema a otro o incoherente) |__| 13102

131.c Habla circunstancial: da muchos detalles innecesarios pero al final alcanza el objetivo que se propone en el habla |__| 13103

131.d Divagante: habla sin apuntar a un tema concreto, divagando, pero las ideas están razonablemente bien formadas |__| 13104

132. Ritmo del habla:

132.a Habla extremadamente rápida pero puede ser interrumpido |__| 13105

132.b Presión del habla: demasiadas palabras, rápido y con pausas infrecuentes y un flujo incontrolado. Habla y habla y se resiste a ser interrumpido |__| 13106

132.c "Fuga de Ideas": Una idea sugiere a otra en rápida sucesión, de tal modo que el paciente pronto ha perdido el hilo de la conversación |__| 13107

132.d Lentitud: Habla muy lenta. Pausas muy bien delimitadas entre palabras |__| 13108

132.e Largas pausas antes de contestar a un rasgo característico |__| 13109

133. Contenido del habla (bromas frecuentes, chistes) |__| 13301

134. Perseveración: Repite las respuestas de manera inapropiada |__| 13401

135. Juicio: Los problemas de memoria son más prominentes que los problemas de pensamiento, p. ej. más dificultad para recordar cosas que para deducirlas. |__| 13501

II-4.2 Dificultades de Comunicación

Valorar solo si contribuyen claramente a las dificultades de comunicación (Extranjero. Por ejemplo, español pobre y entrecortado, se precisa intérprete, marcado acento extranjero o Español que habla aragonés, catalán, gallego... y mal castellano)

136. Defectos físicos:
No valorar aquí los defectos de memoria

136.a Disfasia (debida a la lesión cerebral, no usa/comprende bien las palabras) |__| 13601

136.b Disartria. (No articula o pronuncia bien las palabras por lesión a nivel cerebral) |__| 13502

136.c Disartria (lesión en los órganos fonadores) |__| 13603

136.d Pérdida de dientes |__| 13604

136.e Sordera grave |__| 13605

136.f Ceguera (casi total) |__| 13607

136.g Tartamudeo |__| 13608

136.h Mutismo debido a un defecto físico |__| 13609

136.i Debilidad, cansancio, flojera graves |__| 13610

136.j Otros Especificar:______________________ 13611 |__| 13612

136.k Oligofrenia/baja inteligencia |__| 13613

137. Conductas específicas:

137.a Por ejemplo, hostil, se niega a contestar, objeta las preguntas, reacio a ser entrevistado |__| 13701

137.b Fatiga razonable |__| 13702

137.c Vago, evasivo, respuestas ambiguas. Se contradice, circunstancial (añade detalles innecesarios, pero alcanza el objetivo al final) |__| 13703

137.d Confuso pero NO incoherente |__| 13704

137.e Murmullos, susurros, voz débil |__| 13705

137.f Farfulleo no debido a defecto físico o drogas |__| 13706

137.g Otros. Especificar:______________________ 13706 |__| 13708

137.h Condiciones desfavorables durante entrevista (ruidos, etc.), que pueden distraer |__| 13709

137.i El paciente se duerme repetidamente y hay que despertarle |__| 13710

137.j El paciente parece adormilado en general, pero no se llega a quedar dormido realmente |__| 13711

138. Dificultades de comunicación patológicas:

138.a Defecto de memoria (Claro): Por ejemplo, desorientado, gran pérdida de memoria, clara amnesia temporal e incapaz de recordar sucesos relevantes |__| 13801

138.b Defecto de memoria (dudoso): P. ej., recuerdos vagos, se queja, no convincentemente, de no poder recordar algo, hace pequeños esfuerzos para recordar, memoria no disminuida para tópicos neutrales |__| 13802

138.c Incoherente con conciencia clara: p. ej., irrelevante o raro o respuestas impensadas, ideas no asociadas, charla incoherente, neologismos, perseverancia, fuga de ideas |__| 13803

139. Conducta vívidamente patológica:

139.a P. ej., mantiene su postura ante delirios o alucinaciones |__| 13901

139.b Incorpora al entrevistador en sus delirios |__| 13902

139.c Posturismo (mantiene una postura de cualquier parte del cuerpo extraña o poco común repetidamente o durante varios minutos seguidos), conducta bizarra |__| 13903

139.d Habla para él coherentemente, habla a voces |__| 13904

139.e Llora/grita de manera incontrolable |__| 13905

139.f Presión del habla (no se le puede interrumpir, no deja intervenir al entrevistador) |__| 13906

139.g Gran suspicacia |__| 13907

140. Conducta ambiguamente patológica:

140.a Por ejemplo, mutismo no debido a defecto físico |__| 14001

140.b Suspicaz, negativista (hace claramente lo contrario de lo que el entrevistador le pide o pregunta) |__| 14002

140.c Distanciamiento o apatía posiblemente debidos a indiferencia o a una profunda depresión |__| 14003

140.d Muy hablador, pero no hay habla a presión |__| 14004

140.e Falta de introspección (insight). Niega totalmente los síntomas o la enfermedad |__| 14005

140.f Excitado, agitado, o claramente gozoso/eufórico (no puede quedarse quieto en la silla, o interrumpe frecuentemente con bromas u observaciones) |__| 14006

140.g Preocupado por experiencias internas no declaradas u ocultas (preocupación inespecífica) |__| 14007

140.h Distraibilidad (la atención del entrevistado es atraída frecuentemente por eventos del entorno triviales e irrelevantes) |__| 14008

141. Intoxicación Medicamentosa:

1 No
2 Posiblemente
3 Muy probablemente |__| 14101

II-5 Fiabilidad

Código en caso de ausencia de respuesta:

Por favor complete cada ítem con el número de opción o respuesta apropiados. En caso de ausencia de respuesta utilice el código 99 seguido de alguna de las especificaciones siguientes.

1. *No puede responder por trastorno cognitivo*
2. *No puede hacerlo por limitaciones físicas*
3. *Se niega*
4. *No es apropiado*
5. *Desconoce la respuesta*
6. *Otro*

Juicio: Valorar si hay ERRORES o números 8 en las secciones de orientación o memoria, o si hay numerosos 8 en el resto de la entrevista.

142. Errores cometidos con conciencia clara (no durmiéndose, o bajo la influencia del alcohol o drogas o delirio debido a enfermedad física aguda:

1 Debidos a agitación, depresión, euforia, etc
2 Debidos a un defecto de memoria |__| 14201

143. Errores cometidos con obnubilación de la conciencia (por ej., el entrevistado se estaba durmiendo, bajo la influencia de alcohol, drogas o delirio debido a enfermedad física aguda

1 Sí
2 No |__| 14301

144. La reacción del entrevistado hacia los errores fue poco intensa, indiferente o eufórica

1 Sí
2 No |__| 14401

145. Valoración global de la confianza en los datos

1 Razonable (las respuestas dadas por el entrevistado pueden considerarse apropiadas para más o menos todas las preguntas
2 Pocas dudas
3 Dudas moderadas
4 Dudas graves (el entrevistador considera que el entrevistado fue incapaz o no quiso dar respuestas que pudieran considerarse apropiadas a la mayor parte de las preguntas)
5 Sin valor (respuestas más o menos impensadas, dadas al azar) |__| 14501

Si se duda:

146. Aparte de las dificultades de comunicación las dudas del entrevistador se deben relativamente a:

1 No hay dudas
2 Exageración o tendencia a decir "sí" indiscriminadamente
3 Minimización o tendencia a decir "no" indiscriminadamente |__| 14601

II-6 Resumen Final

Resumen: descripción global del entrevistado basado en la totalidad de la entrevista

Atención, esta sección deberá leerse, por favor UTILICE LETRAS MAYÚSCULAS

____

____

____

____

____

____

____

____

____

____

____

____

____

____

____

____

____

____

____

____

____ 14701

II-7 Diagnóstico Psiquiátrico

149. Este entrevistado, ¿es un caso de demencia?

1 No caso
2 "Borderline" no caso
3 Caso leve
4 Caso moderado
5 Caso grave |__| 14901

II-8 Diagnósticos GMS-HAS

150. Diagnósticos GMS-HAS

	Código CIE-10	Certeza	Grado	Pronóstico
Principal	____________	__________	____________	__________
Principal alternativo	____________	__________	____________	__________
Secundario	____________	__________	____________	__________
Terciario	____________	__________	____________	__________

CÓDIGOS:

Certeza (seguridad) diagnóstica:

1. No hay dudas razonables;
2. Probablemente correcto;
3. Difícil de asegurar, pero en conjunto probable;
4. Muy incierto, pero las alternativas pertenecen a la misma categoría.

Grado de la enfermedad:

1. Leve; 2. Moderado; 3. Grave.

Pronóstico estimado:

1. Debería recuperarse satisfactoriamente sin tratamiento o intervención específica;
2. Debería mejorar satisfactoriamente sin tratamiento o intervención específica;
3. Debería recuperarse completamente con tratamiento o intervención específica;
4. Debería mejorar con tratamiento o intervención específica;
5. Poco probable que se mejore o recupere con cualquier tipo de intervención;
6. Probablemente se deteriore a pesar de toda intervención.

Por favor, anote el tiempo transcurrido de la entrevista hasta este momento |______| 15101

III. Dolor

Código en caso de ausencia de respuesta:

Por favor complete cada ítem con el número de opción o respuesta apropiados. En caso de ausencia de respuesta utilice el código 99 seguido de alguna de las especificaciones siguientes.

1. No puede responder por trastorno cognitivo
2. No puede hacerlo por limitaciones físicas
3. Se niega
4. No es apropiado
5. Desconoce la respuesta
6. Otro

Por favor, tenga en mente que toda esta sección está referida específicamente a las últimas cuatro semanas

152. A continuación voy a preguntarle con algo más de detalles sobre el dolor, ¿ha tenido dolor durante las últimas cuatro semanas?
1 Sí
2 No ***En caso negativo ir a la pregunta 164*** |__| 15201

153.¿Dónde tiene o ha tenido dolores?

a.	Cabeza, rostro, boca	1 Sí 2 No	|__|	15301
b.	Nuca, cuello	1 Sí 2 No	|__|	15302
c.	Espalda (zona dorsal, lumbar)	1 Sí 2 No	|__|	15304
d.	Articulaciones	1 Sí 2 No	|__|	15305
e.	Hombros, brazos, manos	1 Sí 2 No	|__|	15306
f.	Piernas, rodilla, pie	1 Sí 2 No	|__|	15307
g.	Tórax	1 Sí 2 No	|__|	15308
h.	Abdomen,	1 Sí 2 No	|__|	15309
i.	Región genital	1 Sí 2 No	|__|	15310

153.j ¿En qué parte del cuerpo es peor el dolor? ____________________ |__| 15311

Completar código: señalar letra a-i

154. En general, ¿me podría decir cuánto dolor ha tenido durante las últimas cuatro semanas?

Lea las opciones e invite a la persona entrevistada a señalar la adecuada

1 Ninguno
2 Poco
3 Moderado
4 Mucho
5 Muchísimo |__| 15401

155. ¿Con qué frecuencia ha tenido dolor durante las últimas cuatro semanas?

1 Una vez
2 Dos veces
3 A menudo
4 Muy frecuentemente
5 Cada día o casi |__| 15501

156. ¿En qué ocasiones y con motivo de qué ha tenido dolor durante las últimas cuatro semanas? ¿cuánto duró?

En caso de mas de una vez codificar la ocasión con el tiempo mayor

1 Hasta unos minutos
2 Hasta una hora
3 Varias horas
4 Uno o dos días
5 Mas de dos días |__| 15601

157. Durante las últimas cuatro semanas, ¿cuánto afectó el dolor a las siguientes actividades?:

Código: 1. Nada; 2. Poco; 3. Moderadamente; 4. Más bien mucho; 5. Mucho

		Código		
a.	El humor	_______	|__|	15701
b.	La capacidad de moverse y desplazarse	_______	|__|	15702
c.	El sueño	_______	|__|	15703
d.	Dificultades para trabajar incluido trabajo doméstico	_______	|__|	15704
e.	Actividades de tiempo libre	_______	|__|	15705
f.	Capacidad de sentir alegría de vivir	_______	|__|	15706

158. ¿Cuántas veces durante las últimas cuatro semanas ha afectado el dolor a la vida diaria de la persona en estudio?

Señalar número de días de 1 a 28.

Días: _______________ |__| 15801

159. Imagínese que un 1 significa muy poco dolor y un 20 el máximo dolor posible. ¿Podría indicarme un número entre 1 y 20 que corresponde al grado de dolor que ha padecido Vd. en las últimas cuatro semanas?:

Código: 1. Ningún dolor; 20. El peor dolor imaginable

1 2 3 4 5 6 7 8 9 10 11 12 13 14 15 16 17 18 19 20

|__| 15901

160. Utilizando otra vez la misma escala de 1 a 20. ¿Podría indicarme que número correspondería con el nivel máximo de dolor que ha experimentado Vd. en las últimas cuatro semanas?:

Código: 1. Ningún dolor; 20. El peor dolor imaginable

1 2 3 4 5 6 7 8 9 10 11 12 13 14 15 16 17 18 19 20

|__| 16001

161. La persona entrevistada ha recibido algún tratamiento contra el dolor:

1 Ninguno
2 Fármacos, ¿cuáles?____ 16101

3 Otros (p.ej., fisioterapia, estimulación nerviosa), ¿cuáles? _____________________________ 16102

____ 16103

4 Los anteriores y otros, ¿cuáles? ____ 16104

|__| 16105

162. Durante las últimas cuatro semanas, ¿cuánto alivio recibió como resultado de los fármacos u otros tipos de tratamientos contra el dolor?

Señalar lo que mejor describe el alivio que la persona entrevistada obtuvo (0% = ningún alivio; 100% = alivio total)

% 00 20 30 40 50 60 70 80 90 100

|__| 16201

163. Si toma fármacos analgésicos, ¿cuántas horas tarda el dolor en reaparecer tras las tomas?

1 Los fármacos no le ayudan nada
2 Una hora
3 Dos horas
4 Tres horas
5 Cuatro horas
6 Cinco a doce horas
7 Más de doce horas
8 No toma nada |__| 16301

164. *Dolor de cabeza/jaqueca/cefalea:* Tras breve interrogatorio dirigido señale si la persona entrevistada padece actualmente o ha padecido anteriormente dolores de cabeza repetitivos de los tipos siguientes:

Por favor cuando complete esta pregunta ignore la información precedente referida a las últimas cuatro semanas

a. Migraña/jaqueca

b. Cefalea en racimos

c. Cefalea vascular (no clasificada en otra parte)

d. Cefalea debida a tensión

e. Cefalea postraumática crónica

f. Cefalea inducida por medicamentos

g. Otras cefaleas

	Trastorno*	Edad inicio	Cód. 1	Cód. 2	Cód. 3	Cód. 4	Duración	Trat.**	Año	Hospital	AT***
1.	....	....	....	....	....	....	....	....	....	....	.....-.....
2.	....	....	....	....	....	....	....	....	....	....	.....-.....
3.	....	....	....	....	....	....	....	....	....	....	.....-.....
4.	....	....	....	....	....	....	....	....	....	....	.....-.....
5.	....	....	....	....	....	....	....	....	....	....	.....-.....
6.	....	....	....	....	....	....	....	....	....	....	.....-.....
7.	....	....	....	....	....	....	....	....	....	....	.....-.....

Especifique los códigos oportunos:
Frecuencia: *01. Varias veces a la semana; 02. Una vez en semana; 03. Algunas veces al mes; 04. Una vez al mes; 05. Una vez cada dos meses; 06. Menos frecuencia de una vez al mes.*
Localización: *11. en uno de los lados de la cabeza; 12. No localizado.*
Síntomas que precedían el dolor de cabeza: *21. No, 22. Auditivos*; 23. Visuales; 24. Dificultes del lenguaje; 25. Olfativos.
Pulsátil: *31. Sí; 32. No*
Antecedentes familiares: *41. Sí; 42. No*
Duración: *Especifique la duración aproximada en minutos de cada crisis.*
(*) Indique únicamente la letra que identifica el trastorno en la lista anterior
(**) Tipo de asistencia recibida: 1. ambulatoria; 2. ingreso hospitalario. Indique el año y hospital correspondientes
(***) Especificar el intervalo en años si el trastorno se dio anteriormente

D. EXAMEN FÍSICO

Código en caso de ausencia de respuesta:

Por favor complete cada ítem con el número de opción o respuesta apropiados. En caso de ausencia de respuesta utilice el código 99 seguido de alguna de las especificaciones siguientes.

1. *No puede responder por trastorno cognitivo*
2. *No puede hacerlo por limitaciones físicas*
3. *Se niega*
4. *No es apropiado*
5. *Desconoce la respuesta*
6. *Otro*

Por favor proceda a la toma de la tensión arterial solo en el caso de que esta información no vaya a ser tomada durante la administración del Cuadernillo B

166. Tensión arterial 1 mmHg _____ 16601 / _____ 16602

Por favor asegúrese de que los pacientes no hayan fumado, tomado cafeína o comido en los 30 minutos anteriores a la medida y hayan permanecido en reposo los 5 minutos anteriores a la medida. Los pacientes deberían estar relajados y no hablar. El paciente debe estar cómodamente sentado en una silla con el brazo en el que se vaya a medir la TA apoyado y a la altura del corazón y con los pies apoyados en el suelo. Se recomienda el uso de un tensiómetro de mercurio.

167. Tensión arterial 2 mmHg _____ 16701 / _____ 16702

Tome la segunda lectura transcurridos 2 minutos. Si las lecturas varían 5 mmHg o más, realice medidas adicionales.

168. Auscultación pulmonar

1 Normales
2 Auscultación anormal, cual ____________ 16801 lado ______________16802 |__| 16803

1. Roncus; 2. Estertores; 3. Expiración prolongada; 4. Reducción del murmullo vesicular

1.Derecha; 2. Izquierda; 3. Bilateral

169. Espirometría

PEF litros/minuto ___________________________ 16901 |__| 16902

Realizar tres intentos y registrar el resultado superior

170. Auscultación cardiaca

170.a Ritmo

1 Regular
2 Extrasístoles
3 Irregularidades, especificar _______________ 17001 |__| 17002

170.b Soplo

1 Sí, especificar___________________________ 17003
2 No |__| 17004

170.c Soplo cervical (no cardiaco irradiado)

1 Sí, especificar___________________________ 17005
(1. Derecha; 2. Izquierda; 3.Bilateral) |__| 17006
2 No

171. Signos de descompensación cardiaca

171.a Disnea

1 Sí
2 No |__| 17101

171.b Cianosis periférica

1 Sí
2 No |__| 17102

171.a Edema

1 Sí
2 No |__| 17103

172. Intensidad de la insuficiencia cardiaca

1 Asintomática
2 Insuficiencia cardiaca leve/disnea de esfuerzo de grado moderado a intenso
3 Insuficiencia cardiaca leva a moderada
4 Insuficiencia cardiaca con disnea de reposo
5 Encamado |__| 17201

173. Palpación tiroidea

1 Normal
2 Aumentado difuso
3 Aumentado irregular |__| 17301

174. Hipotensión postural

1 Sí
2 No |__| 17401

175. Prótesis ortopédica

175.a ¿Puede la persona entrevistada ponerse y retirarse la prótesis?

1 Sí, por si solo
2 Sí, con ayuda |__| 17501

176. Úlceras de decúbito

1 Si
2 No |__| 17601

Por favor, anote el tiempo transcurrido de la entrevista hasta este momento |_____| 17701

E. CRIBAJE NEUROLÓGICO (Test OMS)

Código en caso de ausencia de respuesta:

Por favor complete cada ítem con el número de opción o respuesta apropiados. En caso de ausencia de respuesta utilice el código 99 seguido de alguna de las especificaciones siguientes.

1. *No puede responder por trastorno cognitivo*
2. *No puede hacerlo por limitaciones físicas*
3. *Se niega*
4. *No es apropiado*
5. *Desconoce la respuesta*
6. *Otro*

A continuación voy a hacerle algunas preguntas sobre síntomas que haya tenido y que pueden estar relacionados con su salud.

La persona entrevistada es cribado como positivo si al menos una de las respuestas seleccionadas (ítems 178-193) está resaltada con un "(*)", salvo en el caso de las preguntas 184 y 184.a, en cuyo caso ambas han de estar resaltadas con un "(*)" para poder cribar al participante como positivo.

I. Cuestionario de síntomas

178. ¿Ha tenido alguna vez episodios de pérdida de conocimiento, es decir, no entender, oír, o ver lo que sucedía a su alrededor, y después no recordar lo ocurrido?

1 Si (más de una vez) (*)
2 Si (una vez) (*)
3 Nunca |__| 17801

179. ¿Ha tenido alguna vez movimientos involuntarios de piernas o brazos?

1 Si (*)
2 No |__| 17901

180. ¿Ha habido cambios importantes en su manera de hablar?

1 Si (*)
2 No |__| 18001

181. ¿Se le ha paralizado en alguna ocasión la cara, o parte de ella, durante más de 24 horas?

1 Si (*)
2 No |__| 18101

182. ¿Se le ha torcido la boca en alguna ocasión durante más de 24 horas?

1 Si (*)
2 No |__| 18201

183. ¿Ha sufrido en alguna ocasión parálisis o debilidad en sus brazos o piernas por un tiempo superior a 24 horas?

1 Si (*)
2 No |__| 18301

184. ¿Ha tenido en alguna ocasión durante más de 24 horas, o por menos tiempo si ha sucedido más de una vez, hormigueo, dolor, quemazón, o pérdida del tacto en sus brazos o piernas sin que hubiera nada que le sucediese inmediatamente antes?

1 Si (*)
2 No |__| 18401

En caso afirmativo lea la pregunta 184.a

184.a ¿Sucedió durante los últimos seis meses?

1 Si (*)
2 No |__| 18402

185.¿Ha tenido alguna vez rigidez o lentitud al moverse?

1 Si (*)
2 No |__| 18501

186.¿Ha tenido alguna vez temblores en la cabeza, brazos, o piernas que durasen más de un día?

1 Si (*)
2 No |__| 18601

II. Tareas físicas

Invitar al participante a sentarse

187. Por favor, cierre sus ojos, ponga los brazos en alto en frente de Vd. con las palmas de las manos abiertas hacia arriba durante 30 segundos.

1 Capaz con ambos brazos
2 No es posible por ____ 18701
3 Incapaz (**) con el brazo derecho (*)
4 Incapaz (**) con el brazo izquierdo (*)
5 Incapaz (**) con ambos brazos (*) |__| 18702

____
(**) Los temblores son una posible explicación.

Invitar al participante a permanecer sentado

188. Por favor, cierre los ojos. Dígame cuál de estos dos objetos es áspero y cuál es suave.

Ofrecer al participante un trozo de seda y un trozo de tejido áspero

1 Capaz con ambas manos
2 No es posible por ____ 18801
3 Incapaz con la mano derecha (*)
4 Incapaz con la mano izquierda (*)
5 Incapaz con ambas manos (*) |__| 18802

Invitar al participante a permanecer sentado

189. Por favor, ponga sus brazos hacia delante. Cierre los ojos, toque su nariz con su dedo índice tres veces. Repítalo con su otra mano.

1 Capaz con ambas manos
2 No es posible por ____ 18901
3 Incapaz con la mano derecha (*)
4 Incapaz con la mano izquierda (*)
5 Incapaz con ambas manos (*) |__| 18902

190. Por favor, camine hacia la puerta primero sobre sus talones y luego de puntillas (*2 metros*).

1 Capaz
2 No es posible por ____ 19001
3 Dificultad al caminar (severa) (*)
4 Incapaz (*) |__| 19002

191. Por favor, permanezca de pie con los pies juntos.

1 Capaz
2 No es posible por ____ 19101
3 Dificultad para estar de pie (*)
4 Incapaz (*) |__| 19102

192. Por favor, cierre los ojos y no se mueva durante unos segundos hasta que se lo indique (*15 segundos*).

Hacer esta pregunta solo si la respuesta a la pregunta 191 es "Capaz"

1 Capaz
2 No es posible por ____ 19201
3 Dificultad para estar de pie (*)
4 Incapaz (*) |__| 19202

193. Ahora voy a comprobar el tono muscular de sus brazos.

Evalúe el tono muscular mediante la manipulación de los brazos dla persona entrevistada (flexión y extensión del codo)

1 Normal
2 Anormal (*)
3 No evaluable por____ 19301 |__| 19304

F. EXAMEN NEUROLÓGICO

I. Exploración neurológica

Código en caso de ausencia de respuesta:

Por favor complete cada ítem con el número de opción o respuesta apropiados. En caso de ausencia de respuesta utilice el código 99 seguido de alguna de las especificaciones siguientes.

1. No puede responder por trastorno cognitivo
2. No puede hacerlo por limitaciones físicas
3. Se niega
4. No es apropiado
5. Desconoce la respuesta
6. Otro

Esta sección constituye una evaluación sistemática. En algunos de los ítems se reseñan resultados de las tareas del cribaje neurológico. En tales casos tome el resultado del ítem señalado y no repita la tarea. Se requerirá contestar positivamente las opciones resaltadas en gris para proceder con la sección posterior referida a parkinsonismos

195. Marcha

Esta pregunta admite respuestas múltiples

1 Normal |__| 19501
2 Hemiparética |__| 19502
3 A pequeños pasos relacionada con la edad |__| 19503
4 Marcha parkinsoniana o con actitud parkinsoniana |__| 19504
5 Con detenciones en los giros |__| 19505
6 No puede caminar |__| 19506
7 Otro, qué (p.ej., con ayuda de bastones)_______________ 00301 |__| 19507

196. Tándem

1 Fundamentalmente normal
2 Patológico |__| 19601

197. Romberg con ojos abiertos

Tomar resultado del ítem 191

1 Normal/discreta inestabilidad
2 Con tendencia a caída hacia__________ 19701 |__| 19702
(1. Derecha; 2. Izquierda; 3. Hacia atrás; 4. Hacia delante; 5. Todas direcciones)

198. Romberg con los ojos cerrados

Tomar resultado del ítem 192

1 Normal/discreta inestabilidad
2 Con tendencia a caída hacia__________ 19801 |__| 19802

(1. Derecha; 2. Izquierda; 3. Hacia atrás; 4. Hacia delante; 5. Todas direcciones)

199. Prueba de dedo nariz

Tomar resultado del ítem 189

1 Normal bilateral
2 Patológica incluyendo la detención_______________ 19901 *(1. Derecha; 2. Izquierda; 3. Bilateral)*
3 Ataxia/temblor, descoordinación_________________ 19902 *(1. Derecha; 2. Izquierda; 3. Bilateral)*

|__| 19903

200. Motricidad

1 Normal
2 Bradicinesia, hipomimía
3 Anormal por parálisis, describir__________________ 00301 *(1. Derecha; 2. Izquierda; 3. Bilateral)*

|__| 20001

201. Movimientos anormales

Esta pregunta admite respuestas múltiples

1 Ninguno, normal
2 Temblor en reposo
3 Temblor de intención
4 Mioclonías
5 Tics/discinesias/movimientos atáxicos
6 Temblor esencial |__| 20101

202. Tono muscular

1 Normal
2 Rigidez o fenómeno de la rueda dentada
3 Espasticidad/clonus en una o varias extremidades
4 Tono muscular disminuido |__| 20201

203. Paresias

1 Ninguna
2 Hemi/monoparesia a la derecha
3 Hemi/monoparesia izquierda
4 Paraparesia/tetrapareasia |__| 20301

204. Grasset *(brazos extendidos y palmas de la mano hacia arriba)*

Tomar resultado del ítem 187

1 Negativo (normal)
2 Descenso y/o pronación derecha
3 Descenso y/o pronación izquierdaº |__| 20401

205. Reflejos

Se admiten respuestas múltiples dentro de cada sección

Anote código cuando proceda: 1. Sí; 2. No

a. Bíceps

1 Normales	|__|	20501
1 Hiperreflexia simétrica y bilateral	|__|	20302
1 Hiperreflexia derecha	|__|	20503
1 Hiperreflexia izquierda	|__|	20504
1 Hiporreflexia simétrica y bilateral	|__|	20505
1 Hipo/parareflexia derecha	|__|	20506
1 Hipo/parareflexia izquierda	|__|	20507

b. Patelar

1 Normales	|__|	20508
1 Hiperreflexia simétrica y bilateral	|__|	20509
1 Hiperreflexia derecha	|__|	20510
1 Hiperreflexia izquierda	|__|	20511
1 Hiporreflexia simétrica y bilateral	|__|	20512
1 Hipo/parareflexia derecha	|__|	20513
1 Hipo/parareflexia izquierda	|__|	20514

c. Babinski

1 Respuesta plantar bilateral normal	|__|	20515
1 Positiva derecha	|__|	20516
1 Positiva izquierda	|__|	20517
1 Positiva bilateral	|__|	20518

206. Reflejos primitivos

Se admiten respuestas múltiples

Anotar código cuando proceda: 1. Derecha; 2. Izquierda

1 Ninguno	_______ 20601	|__|	20602
1 Sí, reflejo de hocico	_______ 20603	|__|	20604
1 Sí, palmomentoniano	_______ 20605	|__|	20606
1 Sí, grasping	_______ 20607	|__|	20608

207. Signos pseudobulbares

a. Disartria	1 Sí	2 No	|__|	20701
b. Disfagia	1 Sí	2 No	|__|	20702
c. Labilidad emocional	1 Sí	2 No	|__|	20703

208. Nervios craneales

Se admiten respuestas múltiples

1 Movimientos oculares y faciales normales	|__|	20801
1 Paresia parpebral, describir.. 20802	|__|	20803
1 Paresia facial (central/periférica), describir.................................... 20804	|__|	20805

209. Reducción del campo visual

1 Ninguna
2 Homónima izquierda
3 Homónima derecha
4 Otra, señalar, ... 20901 |__| 20902

II. Exploración neurológica: Parkinsonismo

Recoger en caso de haber sido positivos los items previos relativas a marcha parkinsoniana, tándem, detenciones en los giros, bradicinesia, corporal o mímica, temblor de reposo, y rigidez con o sin rueda dentada (respuestas subrayadas)

210. Temblor

En caso asimétrico señalar código al lado: 1. Derecha; 2. Izquierda

1 Ninguno
2 Ligero-intermitente _______ 21001
3 Ligero-moderado _______ 21002
4 Marcado _______ 21003 |__| 21004

211. Rigidez

En caso asimétrico señalar código al lado: 1. Derecha; 2. Izquierda

1 Ninguna
2 Solo con la activación _______ 21101
3 Marcada _______ 21102
4 Ligara a moderada _______ 21103 |__| 21104

212. Marcha

1 Normal
2 Lenta sin detenciones
3 Marcha con dificultades, puede fijarse al suelo
4 No puede caminar por si mismo, necesita apoyo o asistencia |__| 21201

213. Bradicinesia

1 Ninguna
2 Mínimo enlentecimiento, dentro de los límites de la normalidad
3 Mínimo pero evidente
4 Moderado, con ciertas dudas o marcado con dificultados |__| 21301

214. Levantarse de una silla

1 Normal (puede haber mas de un intento)
2 Avanza los brazos
3 Cae hacia atrás, pero puede levantarse por sí mismo |__| 21401

215. Postura

1 Normal o antepropulsado (normal para la edad)
2 Anteversión, incluso lateralmente, incluyéndose además anteversión intensa con cifosis inmolado inclinación lateral
3 Marcada tendencia a la flexión marcada con postura claramente anormal |__| 21501

216. Estabilidad en la movilización

Realice un test de provocación empujando desde la espalda con paso hacia adelante

1 Normal, puede dar un par de pasos y ponerse de pie espontáneamente)
2 Se cae si no le sujetan
3 Se cae espontáneamente, no puede mantenerse de pie |__| 21601

217. Giros

1 Giros de pequeña superficie, puede tener dudas pero mantiene la estabilidad
2 Gira lentamente con inseguridad
3 Caería sin ayuda, no puede girar |__| 21701

218. Mímica facial

1 En el límite normal, (incluye ligera facies/jugador de poker)
2 Ligeramente disminuida, pérdida de la expresión facial normal
3 Moderada a marcada disfunción (pérdida total de la mímica facial) |__| 21801

219. Caídas en los últimos seis meses

1 No se ha caído
2 Se ha caído una vez
3 Se ha caído dos o mas veces |__| 21901

Por favor, anote el tiempo transcurrido de la entrevista hasta este momento |______| 22001

G. MEDICACIÓN

Código en caso de ausencia de respuesta:

Por favor complete cada ítem con el número de opción o respuesta apropiados. En caso de ausencia de respuesta utilice el código 99 seguido de alguna de las especificaciones siguientes.

1. No puede responder por trastorno cognitivo
2. No puede hacerlo por limitaciones físicas
3. Se niega
4. No es apropiado
5. Desconoce la respuesta
6. Otro

A continuación voy a preguntarle detalles sobre su medicación actual.

221.¿Toma de medicinas? ¿quién decide el uso de las medicinas?

1 No toma medicinas
2 Toma medicinas por su cuenta
3 Toma medicinas recetadas por el médico
4 (2 + 3) |__| 22101

En caso negativo pase a la sección H

222.¿Le ayuda alguien a tomar las medicinas?, ¿quizá a ordenar las medicinas?, ¿quizá a administrar las medicinas?

1 El propio enfermo cuida su medicación
2 Se reparten en un dosificador por parte de ____ 22201

¿quien? p.ej., ayuda a domicilio, esposo, esposa).

3 Se distribuye por parte de la farmacia en un formato particular
4 El anciano tiene ayuda de otra persona para tomar sus medicinas, ¿de quién?________________ 22202

|__| 22203

223. Medicación actual:

Incluir alimentos dietéticos y fármacos naturales

Nombre comercial del fármaco	Forma de administración	Tipo de preparado*	Contenido de una unidad (mg)	Dosis diaria (en unidades)	Si según necesidad (frecuencia durante un mes)	Tratamiento breve (sí/no)	Fecha aproximada de comienzo	Inicio de tratamiento según documentos o datos del entrevistador	Indicación según datos de la persona	Problemas (según ítem 224 en págin siguiente) Poner letra y número. Ver Ejemplo
**(EJEMPLO) TENORMIN 50mg**	**ORAL**	**1**	**50 mg**	**1**	**DIARIO**	**NO**	**15 / 05 / 05**	**15 / 05 / 05**	**14 / 05 / 05**	**b.2**
							/ /			
							/ /			
							/ /			
							/ /			
							/ /			
							/ /			
							/ /			

(*) Especifique tipo de preparado: 1. Tabletas/efervescente/depot; 2. En polvo; 3. Inyección; 4. Crema; 5. Apósito; 6. Cápsula/cápsula en depósito; 7. Sobre granulado; 8. Spray; 9. Solución o jarabe; 10. Mezcla; 11. Inhalador; 12. Supositorio; 13. Pomada

224.¿Ha tenido dificultades con sus medicinas durante el último mes?

Señale qué fármacos le han solucionado el problema, codificar después la alternativa más adecuada de acuerdo con la lista de abajo. Mirar la última columna de la página anterior

Código: 1. No; 2. Problemas mínimos; 3. Problemas moderados; 4. Problemas importantes

a.	Dificultades para abrir el envase	|__|	22401
b.	Dificultades para comprender las informaciones de la receta	|__|	22402
c.	La dosificación era incómoda, acordarse de todas las tabletas	|__|	22403
d.	Es difícil tomar tantas tabletas a la vez	|__|	22405
e.	Es difícil obtener los fármacos en la farmacia en el momento adecuado	|__|	22406
f.	Era difícil pagar las medicinas	|__|	22407
g.	Estaba muy preocupado por tomar tantas medicinas	|__|	22408
h.	Era difícil tomar la medicina (p.ej., tragar, dejar caer las gotas, etc.)	|__|	22409

225.*¿De qué modo obtiene por parte de la persona entrevistada la información acerca de las medicinas?*

1 Ninguna medicina
2 Se han mostrado la receta
3 Se han mostrado las cajas
4 (1+2)
5 Medicamentos cardex o información de otras personas (familiar, personal asistencial sanitario, etc).
6 La persona lo dijo de memoria
7 (1 + 5)
8 (2 + 5)
9 (3 + 5)
10 (4 + 5) |__| 22501

226.*¿Se han utilizado anteriormente, independientemente de la medicación que reciba actualmente, los siguientes grupos de fármacos?*

Señale el nombre de los fármacos en la lista especial

1	Estrógenos	|__|	22601
2	Antirreumáticos	|__|	22602
3	Ácido acetilsalicílico	|__|	22603
4	Corticoides	|__|	22604
5	Psicofármacos	|__|	22605
6	Neurolépticos	|__|	22506
7	Antidepresivos	|__|	22607
8	Benzodiacepinas	|__|	22608
9	Otros psicofármacos	|__|	22609
10	Medicación antiparkinsoniana	|__|	22610

En caso de marcar alguna de las opciones previas, transcribir el protocolo de administración en la pregunta 227

227. Protocolo de medicación:

Si el preparado se ha administrado en distintos periodos detallar cada una nota para cada una de ellos

	Preparado, contenido de la unidad (mg), dosis (uds)	Fecha comienzo*	Fecha finalización	Administración periódica **
1.	______________	___/___/___	___/___/___	______________
2.	______________	___/___/___	___/___/___	______________
3.	______________	___/___/___	___/___/___	______________
4.	______________	___/___/___	___/___/___	______________
5.	______________	___/___/___	___/___/___	______________
6.	______________	___/___/___	___/___/___	______________
7.	______________	___/___/___	___/___/___	______________
8.	______________	___/___/___	___/___/___	______________
9.	______________	___/___/___	___/___/___	______________
10.	______________	___/___/___	___/___/___	______________

*Con tanta precisión como sea posible.
**Con qué frecuencia y durante cuanto tiempo.

228.*¿De qué modo obtiene por parte de la persona entrevistada la información acerca de las medicinas?*

1 Ninguna medicina
2 Le han mostrado la receta
3 Le han mostrado las cajas
4 (1+2)
5 Medicamentos cardex o información de otras personas (familiar, personal asistencial sanitario, etc).
6 La persona lo dijo de memoria
7 (1 + 5)
8 (2 + 5)
9 (3 + 5)
10 (4 + 5) |__| 22801

H. RESUMEN DE HISTORIA CLÍNICA Y UTILIZACIÓN DE SERVICIOS

Código en caso de ausencia de respuesta:

Por favor complete cada ítem con el número de opción o respuesta apropiados. En caso de ausencia de respuesta utilice el código 99 seguido de alguna de las especificaciones siguientes.

1. No puede responder por trastorno cognitivo
2. No puede hacerlo por limitaciones físicas
3. Se niega
4. No es apropiado
5. Desconoce la respuesta
6. Otro

Las preguntas expresadas en los enunciados de esta sección van dirigidas exclusivamente al entrevistador. Por favor realice cuantas preguntas considere oportunas y revise toda la documentación clínica disponible para contestar adecuadamente este apartado de acuerdo a su propio juicio.

230. *Aparato circulatorio:* ¿Ha presentado la persona entrevistada alguna enfermedad o tratamiento de los señalados a continuación?

a. Angina

b. Infarto de Miocardio

c. Cirugía cardiaca

d. Insuficiencia cardiaca

e. Arritmia

f. Otras enfermedades cardiacas

g. Hipertensión arterial

Condición o intervención*	Edad inicio	Tipo**	Tratamiento***	Año	Hospital	AT****
1.	....	....	....	....	....	.....-.....
2.	....	....	....	....	....	.....-.....
3.	....	....	....	....	....	.....-.....
4.	....	....	....	....	....	.....-.....
5.	....	....	....	....	....	.....-.....
6.	....	....	....	....	....	.....-.....
7.	....	....	....	....	....	.....-.....

(*) Indique únicamente la letra que identifica la enfermedad o tratamiento
(**) Especificar código CIE-10 (a completar en gabinete)
(***) 1. Ambulatoria; 2. Ingreso hospitalario; 3. Otra, especificar (p.ej., tipo de intervención quirúrgica o farmacológica)
(****) En el caso de cardiopatía o hipertensión arterial, especificar el intervalo en años si la condición se dio anteriormente

231. *Enfermedad cerebrovascular:* ¿Ha presentado la persona entrevistada alguna enfermedad de las señaladas a continuación?

a. Accidente isquémico transitorio (AIT)

b. Ictus

Condición	Edad inicio	Tipo*	Tratamiento**	Año	Hospital	AT***
1.	....	....	....	....	....	.....-.....
2.	....	....	....	....	....	.....-.....

(*) Especificar código CIE-10 (a completar en gabinete)
(**) 1. Ambulatoria; 2. Ingreso hospitalario; 3. Otra, especificar (p.ej., tipo de intervención quirúrgica o farmacológica)
(***) Especificar el intervalo en años si la condición se dio anteriormente

232. *Otras enfermedades neurológicas*: ¿Ha presentado la persona entrevistada alguna enfermedad de las señaladas a continuación?

a. Traumatismo craneal con pérdida de conciencia

b. Epilepsia

c. Enfermedad de Parkinson

d. Otras enfermedades neurológicas

Condición	Edad inicio	Tipo*	Tratamiento**	Año	Hospital	AT***
1.	....	....	....	....	....	.....-.....
2.	....	....	....	....	....	.....-.....
3.	....	....	....	....	....	.....-.....
4.	....	....	....	....	....	.....-.....

(*) Especificar código CIE-10 (a completar en gabinete)
(**) 1. Ambulatoria; 2. Ingreso hospitalario; 3. Otra, especificar (p.ej., tipo de intervención quirúrgica o farmacológica)
(***) Especificar el intervalo en años si la condición se dio anteriormente

233. *Enfermedades del aparato respiratorio*: ¿Ha presentado la persona entrevistada alguna enfermedad o condición de las señaladas a continuación?

a. Asma

b. Enfermedad pulmonar obstructiva/enfisema pulmonar

c. Ronquidos

d. Apnea de sueño

e. Tabaquismo

f. Otras enfermedades del aparato respiratorio

Condición	Edad inicio	Tipo*	Tratamiento**	Año	Hospital	AT***
1.	....	....	....	....	....	.....-.....
2.	....	....	....	....	....	.....-.....
3.	....	....	....	....	....	.....-.....
4.	....	....	....	....	....	.....-.....
5.	....	....	....	....	....	.....-.....
6.	....	....	....	....	....	.....-.....

(*) En el caso de tabaquismo indique número de cigarrillos diarios y: 1. Fuma todos los días; 2. No fuma en la actualidad aunque lo ha hecho anteriormente
(**) 1. Ambulatoria; 2. Ingreso hospitalario; 3. Otra, especificar (p.ej., tipo de intervención quirúrgica o farmacológica)
(***) Especificar el intervalo en años si la condición se dio anteriormente

234. *Enfermedades oftalmológicas*: ¿Ha presentado la persona entrevistada alguna enfermedad de las señaladas a continuación?

a. Cataratas

b. Glaucoma

c. Degeneración macular, retinopatía degenerativa

d. Otras enfermedades oftalmológicas

Enfermedad	Edad inicio	Tipo*	Tratamiento**	Año	Hospital	AT***
1.	....	....	....	....	....	.....-.....
2.	....	....	....	....	....	.....-.....
3.	....	....	....	....	....	.....-.....
4.	....	....	....	....	....	.....-.....

(*) Especificar código CIE-10 (a completar en gabinete)
(**) 1. Ambulatoria; 2. Ingreso hospitalario; 3. Otra, especificar (p.ej., tipo de intervención quirúrgica o farmacológica)
(***) Especificar el intervalo en años si la condición se dio anteriormente

235. *Enfermedades autoinmunes*: ¿Ha presentado alguna enfermedad de las señaladas a continuación?

a. Artritis reumatoide

b. Lupus eritematoso diseminado

c. Vasculitis

d. Polimialgia reumática

e. Otras enfermedades reumáticas inflamatorias

Enfermedad	Edad inicio	Tipo*	Tratamiento**	Año	Hospital	AT***
1.	....	....	....	....	....	.....-.....
2.	....	....	....	....	....	.....-.....
3.	....	....	....	....	....	.....-.....
4.	....	....	....	....	....	.....-.....
5.	....	....	....	....	....	.....-.....

(*) Especificar código CIE-10 (a completar en gabinete)
(**) 1. Ambulatoria; 2. Ingreso hospitalario; 3. Otra, especificar (p.ej., tipo de intervención quirúrgica o farmacológica)
(***) Especificar el intervalo en años si la condición se dio anteriormente

236. *Enfermedades endocrinológicas*: ¿Ha presentado la persona entrevistada alguna enfermedad de las señaladas a continuación?

a. Diabetes tipo 1 (de comienzo temprano, insulinodependiente)

b. Diabetes tipo 2 (diabetes del edad media o anciano, no es necesariamente insulinodependiente)

c. Hipertiroidismo

d. Hipotiroidismo

e. Otras enfermedades endocrinológicas

Enfermedad	Edad inicio	Tipo*	Tratamiento**	Año	Hospital	AT***
1.	....	....	....	....	....	.....-.....
2.	....	....	....	....	....	.....-.....
3.	....	....	....	....	....	.....-.....
4.	....	....	....	....	....	.....-.....
5.	....	....	....	....	....	.....-.....
6.	....	....	....	....	....	.....-.....

(*) Especificar código CIE-10 (a completar en gabinete). En el caso de Diabetes tipo II, especificar: 1. dietético; 2. 1+ tableta; 3. 1+ insulina; 4. los tres
(**) 1. Ambulatoria; 2. Ingreso hospitalario; 3. Otra, especificar (p.ej., tipo de intervención quirúrgica o farmacológica)
(***) Especificar el intervalo en años si la condición se dio anteriormente

237. *Fracturas y enfermedades del sistema locomotor*: ¿Ha presentado la persona entrevistada alguna enfermedad de las señaladas a continuación?

a. Artrosis

b. Fractura de cadera

c. Otra fractura

d. Aplastamiento vertebral

e. Osteoporosis

Condición	Edad inicio	Tipo*	Tratamiento**	Año	Hospital	AT***
1.	....	....	....	....	....	.....-.....
2.	....	....	....	....	....	.....-.....
3.	....	....	....	....	....	.....-.....
4.	....	....	....	....	....	.....-.....

(*) Especificar código CIE-10 (a completar en gabinete). En el caso de Fractura de cadera o de otra fractura, especificar: 1. derecha; 2. izquierda. En caso de aplastamiento vertebral indicar: 1. torácica; 2. lumbar; 3. 1+2
(**) 1. Ambulatoria; 2. Ingreso hospitalario; 3. Otra, especificar (p.ej., tipo de intervención quirúrgica o farmacológica)
(***) Especificar el intervalo en años si la condición se dio anteriormente

238. *Enfermedades infecciosas*: ¿Ha presentado la persona entrevistada alguna enfermedad de las señaladas a continuación?

a. Meningitis o encefalitis

b. Polio

c. Tuberculosis

d. Borreliosis

e. Otra infección

Enfermedad	Edad inicio	Tipo*	Tratamiento**	Año	Hospital	AT***
1.	....	....	....	....	....	.....-.....
2.	....	....	....	....	....	.....-.....
3.	....	....	....	....	....	.....-.....
4.	....	....	....	....	....	.....-.....
5.	....	....	....	....	....	.....-.....
6.	....	....	....	....	....	.....-.....

(*) Especificar código CIE-10 (a completar en gabinete). En caso de tuberculosis, especificar: 1. Pulmonar; 2. Renal; 3. Sistema nervioso
(**) 1. Ambulatoria; 2. Ingreso hospitalario; 3. Otra, especificar (p.ej., tipo de intervención quirúrgica o farmacológica)
(***) Especificar el intervalo en años si la condición se dio anteriormente

239. *Cáncer*: ¿Ha presentado algún trastorno oncológico de los tipos y localizaciones señalados a continuación?

a. Labio, cavidad bucal y faringe

b. Órganos digestivos

c. Órganos respiratorios e intratorácicos

d. Huesos y cartílagos articulares

e. Piel

f. Tejidos mesoteliales y tejidos blandos

g. Mama

h. Órganos genitales femeninos

i. Órganos genitales masculinos

j. Vías urinarias

k. Ojo, encéfalo u otras partes del sistema nervioso central

l. Tumores malignos de sitios mal definidos, secundarios y de sitios no especificados

m. Tumores malignos (declarados o presuntos como primarios) del tejido linfático, de los órganos hematopoyéticos y de tejidos afines

n. Tumores malignos (primarios) de sitios múltiples independientes

ñ. Tumores in situ

o. Tumores benignos

p. Tumores de comportamiento incierto o desconocido

Enfermedad	Edad inicio	Tipo*	Tratamiento**		Año	Hospital	AT***
1.	....	....	....	....	....	....	.....-.....
2.	....	....	....	....	....	....	.....-.....
3.	....	....	....	....	....	....	.....-.....
4.	....	....	....	....	....	....	.....-.....
5.	....	....	....	....	....	....	.....-.....
6.	....	....	....	....	....	....	.....-.....
7.	....	....	....	....	....	....	.....-.....
8.	....	....	....	....	....	....	.....-.....
9.	....	....	....	....	....	....	.....-.....
10.	....	....	....	....	....	....	.....-.....

(*) Especificar código CIE-10 (a completar en gabinete)
(**) Especificar tipo de atención recibida: 1. Ambulatoria; 2. Ingreso hospitalario y tipo de tratamiento: 1. Operación; 2. Quimioterapia; 3. Radioterapia
(***) Especificar el intervalo en años si la condición se dio anteriormente

240. *Trastornos mentales*: ¿Ha presentado la persona entrevistada alguna de las condiciones o trastornos de los señalados a continuación?

a. Depresión (previamente)

b. Psicosis (enfermedad bipolar, esquizofrenia)

c. Demencia o episodios confusionales

d. Estrés Psicológico

e. Trastornos del desarrollo (retraso mental, etc.)

f. Otras manifestaciones (trastornos de ansiedad, del sueño, de personalidad, etc.)

	Trastorno	Edad inicio	Tipo*	Tratamiento**	Año	Hospital	AT***
1.	....	....	....	....	....	....	.....-.....
2.	....	....	....	....	....	....	.....-.....
3.	....	....	....	....	....	....	.....-.....
4.	....	....	....	....	....	....	.....-.....
5.	....	....	....	....	....	....	.....-.....
6.	....	....	....	....	....	....	.....-.....

(*) Especificar código CIE-10 (a completar en gabinete)
(**) Especifique tipo de atención recibida: 1. Ambulatoria; 2. Ingreso hospitalario y modalidad terapéutica: 1. farmacológica; 2. cirugía o electroshock; 3. psicoterapéutica psicodinámica; 4. psicoterapéutica cognitivo-conductual; 5. Otras psicoterapias; 6. combinación fármacos-psicoterapia
(***) Especificar el intervalo en años si la condición se dio anteriormente

241. *Otras enfermedades (urológicas, gastroenterológicas, ginecológicas o de la piel)*: ¿Ha presentado la persona entrevistada alguna otra enfermedad no señalada previamente?

	Enfermedad	Edad inicio	Tipo*	Tratamiento**	Año	Hospital	AT***
1.	....	....	....	....	....	....	.....-.....
2.	....	....	....	....	....	....	.....-.....
3.	....	....	....	....	....	....	.....-.....
4.	....	....	....	....	....	....	.....-.....
5.	....	....	....	....	....	....	.....-.....

(*) Especifique código CIE-10 siempre que sea posible.
(**) 1. Ambulatoria; 2. Ingreso hospitalario; 3. Otra, especificar (p.ej., tipo de intervención quirúrgica o farmacológica)
(***) Especificar el intervalo en años si la condición se dio anteriormente

I. ESCALA DE IMPACTO FUNCIONAL DE ENFERMEDADES POR ÓRGANOS, APARATOS O SISTEMAS

Por favor use los códigos siguientes para calificar cada órgano o sistema:

1. Ninguna	*Ninguna pérdida de función en este sistema u órgano*
2. Leve	*Leve pérdida de función que no altera la actividad normal, tratamiento no siembre necesario*
3. Moderado	*Pérdida de función que altera la actividad normal del individuo y requiere tratamiento*
4. Grave	*La pérdida de función es notablemente discapacitante y demanda tratamiento inmediato*
5. Muy grave	*Pérdida de función que supone riesgo vital y demanda tratamiento inmediato o no es posible tratarlo*

Código en caso de ausencia de respuesta:

Por favor complete cada ítem con el número de opción o respuesta apropiados. En caso de ausencia de respuesta utilice el código 99 seguido de alguna de las especificaciones siguientes.

1. No puede responder por trastorno cognitivo
2. No puede hacerlo por limitaciones físicas
3. Se niega
4. No es apropiado
5. Desconoce la respuesta
6. Otro

243. Corazón |__| 24301

Solamente

244. Hipertensión |__| 24401

El puntaje se basa en la gravedad. Los diferentes órganos se puntúan en los ítems a continuación

245. Aparato circulatorio |__| 24501

Incluidos sangre, vasos, células sanguíneas, médula, ganglios linfáticos, bazo

246. Aparato respiratorio |__| 24601

Pulmón, bronquios, tráquea, laringe

247. Ojos y garganta, nariz y oído |__| 24701

248. Órganos gastrointestinales y tracto gastrointestinal inferior |__| 24801

249. Hígado |__| 24901

Solamente

250. Riñón |__| 25001

Solamente

251. Aparato genital y urinario |__| 25101

Uretra, próstata, vejiga y genitales

252. Sistema músculoesqulético y piel |__| 25201

253. Sistema endocrino y metabólico |__| 25301

Incluye diabetes, diseminada, intoxicación

254. Sistema nervioso I |__| 25401

Cerebro, médula, nervios periféricos, no incluye demencia

255. Sistema nervioso II: Demencia y deterioro cognitivo |__| 25501

256. Trastornos psiquiátricos y de conducta |__| 25601

Incluye depresión, ansiedad, agitación, psicosis, no incluye demencia

257. Notas |__| 25701

1 Hay notas
2 No hay notas

....

....

.... 25702

258. La entrevista ha sido contestada por:

1 Por la persona entrevistada
2 Por un familiar o amigo, especificar ____________________________________ 25801

1: Esposo/a, Pareja; 2: Padre/Madre, Suegro/Suegra; 3: Hijo/a; 4: Yerno, Nuera; 5: Nieto/a; 6: Hermano/a, Cuñado/a; 7: Otro Familiar; 8: Empleado/a de hogar, persona de servicio;9: Otras Personas; 10: el entrevistador

3 Por la persona entrevistada con la ayuda de un familiar o amigo, especificar ___________________ 25802

(En caso de que la persona entrevistada haya requerido ayuda en al menos una sección)

|__| 25803

En caso de que la entrevista haya sido contestada con la ayuda de un familiar o amigo, especificar (marque un uno en la casilla correspondiente):

258.a Secciones contestadas fundamentalmente por el entrevistado (*1. Si; 2. No*)

1 Consentimiento informado |__| 25804
1 Depresión y estado mental (GMS-B) |__| 25805
1 Cribaje neurológico (Test OMS) |__| 25806
1 Examen clínico general (SNAC-K) |__| 25807

258.b Secciones contestadas fundamentalmente por el familiar o amigo (*1. Si; 2. No*)

1 Consentimiento informado |__| 25808
1 Depresión y estado mental (GMS-B) |__| 25809
1 Cribaje neurológico (Test OMS) |__| 25810
1 Examen clínico general (SNAC-K) |__| 25811

Por favor, anote el tiempo transcurrido de la entrevista hasta este momento |______| 25901

Por favor revise rápidamente el cuadernillo y cerciórese de que todas las secciones han sido cumplimentadas correctamente y todos los ítems han sido administrados o, en caso de ausencia de respuesta, han sido codificados adecuadamente

Por favor si no obtuvo el consentimiento informado por escrito al comienzo de la entrevista obténgalo en este momento

La entrevista termina aquí

I. RECAPITULACIÓN: DIAGNÓSTICOS, ESTADO DE SALUD ACTUAL Y CRIBAJES

Esta sección va dirigida al entrevistador y debe ser completada en el despacho

Uno de los objetivos de este estudio es detectar la presencia de ciertas enfermedades no diagnosticadas con repercusión sobre la discapacidad. Esta entrevista-examen (cuaderno A) posibilita: 1) el cribaje neurológico y cognitivo; 2) el diagnóstico de ciertos síndromes neurológicos o psiquiátricos; 3) la identificación de diagnósticos establecidos por los servicios sanitarios, y 4) la evaluación del impacto actual de la patología observada sobre la capacidad funcional. La segunda entrevista (cuaderno B), orientada hacia el funcionamiento y la calidad de vida, se desarrolla de forma independientemente del conocimiento diagnóstico.

Esta sección ha de ser un trabajo conjunto y de despacho de los entrevistadores médico y psicosocial y del responsable del equipo investigador local, destinada a evaluar dos aspectos; uno relacionado con objetivos del estudio longitudinal y otro sobre la estimación de la prevalencia de demencias:

1. Los investigadores evaluarán la existencia de perdida de función-discapacidad constatada en los cuadernos A y B que no puedan explicarse por la patología hasta el momento identificada (cuadro 1) y la existencia de positividad al cribaje tanto neurológico como cognitivo (cuadro 2). La constatación de este tipo de pérdida de función en el momento del estudio longitudinal implicará la comunicación con el médico de cabecera acerca de la conveniencia de continuar el estudio diagnóstico (p.ej., si se identifica una inestabilidad en la marcha o una astenia importante de causa no conocida).

2. El segundo objetivo se refiere a la pertinencia de iniciar la fase de post-cribaje del estudio de prevalencia de demencias que incluye su clasificación etiológica. Ello supone el estudio diagnóstico y protocolizado de la demencia, la filiación del parkinsonismo en caso de que acompañe al cuadro y la identificación de posibles antecedentes de enfermedad cerebrovascular. El resultado de este cribaje y el diagnóstico se registrarán en el cuadro 2. Cada grupo interesado y capacitado por las condiciones del medio geográfico/social para contribuir al estudio de prevalencia de demencias recibirá para su uso en segunda fase un protocolo diagnóstico estándar de demencias. Es importante señalar que a diferencia del cribaje cognitivo el cribaje neurológico no es objeto de segunda fase; su aplicación se llevará a cabo en el marco del estudio longitudinal.

Cuadro 1. Concordancia de diagnósticos establecidos o validados por entrevista/examen con el nivel de discapacidad consensuado por entrevistadores médico (impacto estimado de la perdida de función por O/A/S en discapacidad) y psico-social (WHO-DAS II y Katz)

		Clasificaciones CIE-10	Concordancia de diagnósticos con impacto en discapacidad		
**Diagnósticos GMS–HAS** (Secc. II-8, ítem 150, pág. 28)	1.	....	1 Sí 2 No	|__|	00201
	2.	....	1 Sí 2 No	|__|	00201
	3.	....	1 Sí 2 No	|__|	00201
	4.	....	1 Sí 2 No	|__|	00201
**Diagnósticos enfermedades físicas en la actualidad**	1.	....	1 Sí 2 No	|__|	00201
	2.	....	1 Sí 2 No	|__|	00201
	3.	....	1 Sí 2 No	|__|	00201
	4.	....	1 Sí 2 No	|__|	00201
**Ambos tipos de diagnósticos conjuntamente y sus niveles de gravedad...**			¿Explican el nivel de discapacidad constatado?		
			1 Sí 2 No	|__|	00201

Cuadro 2. Positividad a cribajes y planificación de recogida de datos para la segunda fase (estudio de prevalencia de demencias por tipos etiológicos según protocolo, se incluyen la identificación de historia clínica cerebrovascular y del tipo de parkinsonismos entre los positivos al cribaje neurológico)

**Positividad al cribaje neurológico** (Secc. E, ítems 178-193, pág. 33-35)	En al menos una pregunta o tarea	1 Sí 2 No	|__|	00201
	Sospecha de presencia de patología no diagnosticada*	1 Sí 2 No	|__|	00201
	Documentación clínica disponible	1 Sí 2 No	|__|	00201
	Hospital/centro	....		
**Positividad al cribaje cognitivo** (Secc. I-1.3, ítems 28-39, pág. 12-14)	MEC < 24 puntos	1 Sí 2 No	|__|	00201
	Se sospecha patología no diagnosticada*	1 Sí 2 No	|__|	00201
	Pertinencia de estudio protocolizado en segunda fase	1 Sí 2 No	|__|	00201
	Documentación clínica disponible	1 Sí 2 No	|__|	00201
	Hospital/centro	....		

(*) Incluir solo patologías relacionada con la positividad a los cribajes que sean capaces de explicar el diferencial de discapacidad no atribuible a enfermedad reconocida

Clínico responsable del en segunda fase (i.e., estudio diagnóstico de demencia y subtipos)

Nombre y apellidos ____

Hospital Centro Sanitario ____

Teléfono de contacto ____

Centro Nacional de Epidemiología

Estudio longitudinal del envejecimiento de poblaciones españolas

Cuaderno B

Función y calidad de vida

Número de entrevista ☐☐☐ 00001

Código de provincia ☐☐ 00002

Índice

A. IDENTIFICACIÓN DEL ENTREVISTADO *4*

B. MEDIDAS ANTROPOMÉTRICAS *5*

C. DISCAPACIDAD 6

I. Cuestionario para la Discapacidad WHO-DAS II (12 ítems) 7

II. Cuestionario para la Discapacidad WHO-DAS II (36 ítems) *7*

II-1. Datos demográficos y de índole general *8*

II-2. Preámbulo *8*

II-3. Comprensión y comunicación *9*

II-4. Capacidad para moverse en su alrededor/entorno *9*

II-5. Cuidado personal *10*

II-6. Relacionarse con otras personas *10*

II-7. Actividades de la vida diaria *11*

II-8. Actividad laboral *11*

II-9. Participación en sociedad *12*

III. Índice de Katz *13*

D. CALIDAD DE VIDA (EuroQoL) *15*

E. EVALUACIÓN DEL ESTADO NUTRICIONAL (MNA) *16*

I. Cribaje *16*

II. Evaluación *18*

F. FACTORES SOCIALES *21*

I. Hogar y relación de parentesco *21*

II. Entorno físico: Vivienda *21*

III. Entorno social: Red, apoyo y participación *22*

III-1. Red familiar y social *22*

III-2. Apoyo Social *23*

III-3. Participación Social *23*

IV. Estudios, relación con la actividad y ocupación *25*

V. Situación económica *26*

G. AFRONTAMIENTO (SOC) *27*

H. CARGA DEL CUIDADOR (Zarit) *29*

I. AFRONTAMIENTO DEL CUIDADOR (ISPE) *31*

J. MATERIALES ADICIONALES *33*

Estimada/o colega, este es el cuestionario sobre función y calidad de vida del estudio longitudinal del envejecimiento de poblaciones españolas diseñado por el Grupo Español de Estudios Epidemiológicos del Envejecimiento. Este cuadernillo está diseñado para que Vd. lea los enunciados en voz alta al participante; los mensajes tabulados en cursiva están dirigidos a Vd. y por tanto no deben ser leídos en voz alta. Por favor lea atentamente las instrucciones correspondientes a cada sección.

A. IDENTIFICACIÓN DEL ENTREVISTADO

Código en caso de ausencia de respuesta:

Por favor complete cada ítem con el número de opción o respuesta apropiados. En caso de ausencia de respuesta utilice el código 99 seguido de alguna de las especificaciones siguientes.

1. *No puede responder por trastorno cognitivo*
2. *No puede hacerlo por limitaciones físicas*
3. *Se niega*
4. *No es apropiado*
5. *Desconoce la respuesta*
6. *Otro*

1. Identificación del entrevistado

Nombre :	______________________________	00101
Dirección postal:	______________________________	00102
Código postal:	☐☐☐☐☐	00103
Teléfono para contactos posteriores:	______________________________	00104
Fecha de la entrevista:	______________________________	00105

2. Muestra

Completar por el entrevistador antes de iniciar la entrevista, marque solo una respuesta

1 Población general
2 Problemas relacionados con las drogas
3 Problemas de salud mental
4 Problemas físicos |__| 00201
5 Otros (especificar: ____________________) 00201

3. Sexo

Según apariencia

1 Femenino
2 Masculino |__| 00301

4. ¿Qué edad tiene? años ____ /____ 00401

5. ¿Cuál es la fecha exacta de su nacimiento? ___ / ___ /___ 00501

Formato DD/MM/AAAA

6. ¿Cuál es su estado civil actual?

Seleccione la opción más adecuada

1 Soltero/a
2 Casado/a
3 Separado/a
4 Divorciado/a
5 Viudo/a
6 Viviendo en pareja |__| 00601

7. Identificación del hábitat de residencia (municipio, provincia, tamaño municipal, condiciones vivienda).

Nombre del municipio	______________________________		00701
Código INE (*)	□□□□		00702
Nombre de la provincia	______________________________		00703
Tamaño municipal (nº habitantes) (*)	______________________________		00704
¿Tiene ascensor la vivienda?	Sí 1 No 2	|__|	00705
Para acceder a la vivienda es necesario el uso de: (pueden marcarse más de una)	Ascensor 1<br>Escaleras 2<br>Ascensor y escaleras 3	|__|	00706

____
(*) Anotar en gabinete.

B. MEDIDAS ANTROPOMÉTRICAS

Código en caso de ausencia de respuesta:

Por favor complete cada ítem con el número de opción o respuesta apropiados. En caso de ausencia de respuesta utilice el código 99 seguido de alguna de las especificaciones siguientes.

1. *No puede responder por trastorno cognitivo*
2. *No puede hacerlo por limitaciones físicas*
3. *Se niega*
4. *No es apropiado*
5. *Desconoce la respuesta*
6. *Otro*

9. Peso Kg __________ 00901

Por favor, utilice una la báscula calibrada de este estudio. Solicite al participante que se descalce, así como que se quite prendas de abrigo pesadas.

10. Talla cm __________ 01001

Use el tallímetro. La medida debe tomarse estando el participante descalzo.

Calcule el Índice de Masa Corporal (IMC= peso/talla ^{2} en kg/m^2) _______ / _______ ^{2} = _______

11. Tensión arterial 1 mmHg _____ 01101 / _____ 01102

Sólo si no ha sido registrada previamente durante la administración del Cuadernillo A. Por favor asegúrese de que los pacientes no hayan fumado, tomado cafeína o comido en los 30 minutos anteriores a la medida y hayan permanecido en reposo los 5 minutos anteriores a la medida. Los pacientes deberían estar relajados y no hablar. El paciente debe estar cómodamente sentado en una silla con el brazo en el que se vaya a medir la TA apoyado y a la altura del corazón y con los pies apoyados en el suelo. Se recomienda el uso de un tensiómetro de mercurio. Medir la tensión en ambos brazos y elegir el que tenga la presión más alta.

12. Tensión arterial 2 mmHg _____ 01201 / _____ 01202

Tome la segunda lectura transcurridos 2 minutos. Si las lecturas varían 5 mmHg o más, realice medidas adicionales.

13. Otras medidas:

13.a Circunferencia braquial cm ____________ 01301

Pida al participante que sitúe el codo de su brazo no dominante en ángulo recto manteniendo hacia arriba la palma de la mano. Mida la distancia desde la escápula al codo en la parte posterior del brazo; marque el punto medio con un bolígrafo o similar. Pida al paciente que deje el brazo colgar libremente y mida el perímetro del brazo en ese punto. Aproximar al milímetro mas cercano

13.b Circunferencia de la pantorrilla cm _____ _______01302

El participante debe estar sentado con las piernas relajadas y sueltas o de pie con el peso distribuido homogéneamente entre ambas piernas. Pida al participante que descubra la zona de la pantorrilla. Tome la primera medida en la zona mas ancha de la pantorrilla. Tome otras medidas en zonas inmediatamente adyacentes para comprobar que la primera lectura corresponde al punto de mayor perímetro. La medida se considerará correcta solo si la cinta esta perpendicular al eje de la pantorrilla. Aproximar al milímetro más cercano

13.c Altura talón-rodilla cm _____ 01303

El participante debe estar sentado con las piernas relajadas y la rodilla flexionada en ángulo recto. Mida desde la parte exterior de la rodilla hasta el punto de contacto del talón con el suelo. Aproximar al milímetro más cercano

Por favor, anote el tiempo transcurrido de la entrevista hasta este momento |_____| 01401

C. DISCAPACIDAD

Código en caso de ausencia de respuesta:

Por favor complete cada ítem con el número de opción o respuesta apropiados. En caso de ausencia de respuesta utilice el código 99 seguido de alguna de las especificaciones siguientes.

1. *No puede responder por trastorno cognitivo*
2. *No puede hacerlo por limitaciones físicas*
3. *Se niega*
4. *No es apropiado*
5. *Desconoce la respuesta*
6. *Otro*

I. Cuestionario para la Discapacidad WHO-DAS II (12 ítems)

Por favor, al leer enfatice el texto subrayado, utilice los ejemplos y explicaciones que aparecen entre paréntesis con objeto de aclarar al entrevistado el significado de las preguntas. La información en cursiva entre paréntesis es para Vd. y no debe leerse al entrevistado. Si el participante adelanta información repita la pregunta en el momento adecuado aunque de forma confirmatoria (p.ej., entonces decía Vd. que ha tenido dificultades para realizar sus quehaceres, etc.). Durante al entrevista mantenga un clima adecuado mediante frases neutras. Si el paciente divaga sobre aspectos irrelevantes a la pregunta sugiera tratar tales aspectos al término de la entrevista. Si es necesario clarifique y vuelva a repetir la pregunta.

Muestre las tarjetas 1 y 2			Ninguna	Leve	Moderada	Grave	Extrema/ No puedo hacerlo
**15.** ¿Cuánta dificultad ha tenido para estar de pie durante largos periodos de tiempo, como por ejemplo 30 minutos?	|__|	01501	1	2	3	4	5
**16.** ¿Cuánta dificultad ha tenido para cumplir con sus quehaceres de la casa?	|__|	01601	1	2	3	4	5
**17.** ¿Cuánta dificultad ha tenido para aprender una nueva tarea, por ejemplo, llegar a un lugar en donde nunca ha estado?	|__|	01701	1	2	3	4	5
**18.** ¿Cuánta dificultad ha tenido para participar, al mismo nivel que el resto de las personas, en actividades de la comunidad (por ejemplo fiestas, actividades religiosas u otras actividades?	|__|	01801	1	2	3	4	5
**19.** ¿Cuánto le ha afectado emocionalmente su estado de salud?	|__|	01901	1	2	3	4	5
**20.** ¿Cuánta dificultad ha tenido para concentrarse en hacer algo durante diez minutos?	|__|	02001	1	2	3	4	5
**21.** ¿Cuánta dificultad ha tenido para caminar largas distancias, como un kilómetro (o algo equivalente)?	|__|	02101	1	2	3	4	5
**22.** ¿Cuánta dificultad ha tenido para bañarse/lavarse todo el cuerpo?	|__|	02201	1	2	3	4	5
**23.** ¿Cuánta dificultad ha tenido para vestirse?	|__|	02301	1	2	3	4	5
**24.** ¿Cuánta dificultad ha tenido para relacionarse con personas que no conoce?	|__|	02401	1	2	3	4	5
**25.** ¿Cuánta dificultad ha tenido para mantener una amistad?	|__|	02501	1	2	3	4	5
**26.** ¿Cuánta dificultad ha tenido para llevar a cabo su trabajo diario?	|__|	02601	1	2	3	4	5

Si el participante no contesta ninguna pregunta con "1" (ninguna dificultad) pase al punto III, si el participante contesta alguna pregunta con "1" (ninguna dificultad) pase el WHO-DAS de 36 ítems

II. Cuestionario para la Discapacidad WHO-DAS II (36 ítems)

A completar por el entrevistador antes de iniciar la entrevista, marque solo una respuesta

27. ¿Cómo vive el participante en el momento de la entrevista?

1 Vive de forma independiente en la comunidad
2 Vive recibiendo apoyo
3 Hospitalizado |__| 02701

A completar por el entrevistador antes de iniciar la entrevista, marque solo una respuesta

28. Muestra

1 Población general
2 Problemas relacionados con las drogas
3 Problemas de salud mental
4 Problemas físicos
5 Otros Especificar: ANCIANOS |_5_| 02801

II-1. Datos demográficos y de índole general

En caso de población clínica leer lo siguiente:

Esta entrevista ha sido desarrollada por la Organización Mundial de la Salud con el fin de llegar a entender mejor las dificultades que puedan tener las personas debido a su estado de salud. La información que nos proporcione durante esta entrevista será confidencial y utilizada exclusivamente con fines de investigación.

En caso de población general leer lo siguiente:

Aunque usted esté sano/a y no tenga dificultades, me gustaría hacerle todas las preguntas incluidas en esta entrevista, para así obtener una información completa.

II-2. Preámbulo

Diga al entrevistado:

El resto de esta entrevista trata sobre las dificultades que las personas tienen debido a su estado de salud.

Entregue la tarjeta 1 al entrevistado

Por estado de salud me refiero a una enfermedad u otros problemas de salud de corta o larga duración, lesiones, problemas mentales o emocionales (o de lo nervios) y problemas relacionados con el uso de alcohol o drogas. Le recuerdo que, cuando conteste a las preguntas, piense en todos sus problemas de salud. Cuando le pida que me hable acerca de las dificultades que tiene a la hora de realizar una actividad, por favor, tome en consideración los siguientes aspectos.

Señale la tarjeta 1

- Aumento del esfuerzo
- Malestar o dolor
- Lentitud
- Cambios en el modo en que realiza la actividad

Señale la tarjeta 1

A lo largo de toda esta entrevista, cuando esté respondiendo a cada pregunta me gustaría que pensara en los últimos 30 días. Me gustaría que respondiera estas preguntas pensando en cuanta dificultad tiene usted cuando realiza esta actividad según acostumbra a hacerlo.

Entregue la tarjeta 2 al entrevistado
Cuando responda a las preguntas, utilice esta escala (*Lea la escala en voz alta*): Ninguno/a, leve, moderado/a, grave, extremo/ o no puede hacerlo.

Las tarjetas 1 y 2 han de permanecer a la vista del entrevistado durante toda la entrevista. La tarjeta 3 se utiliza en aquellos ítems en los que se le pide que diga el número de días en los que tuvo la dificultad

II-3. Comprensión y comunicación

Ahora le voy a leer algunas preguntas sobre comprensión y comunicación

Muestre las tarjetas 1 y 2.
Si alguno de los ítems se puntúa con "1" (ninguna dificultad pregunte:* ¿Cuántos días ha estado presente esta dificultad? *y anote el número de días (0-30)

En los últimos 30 días, ¿cuánta dificultad ha tenido para:		Ninguno/a	Leve	Moderado/a	Grave	Extremo/a No puedo hacerlo	
**29.** Concentrarse en hacer algo durante diez minutos?	|__| 02901	1	2	3	4	5	_____ 02902
**30.** Recordar las cosas importantes que tiene que hacer? (*no importa que para ello requiera ayudas externas*)	|__| 03001	1	2	3	4	5	_____ 03002
**31.** Analizar y encontrar soluciones a los problemas de la vida diaria?	|__| 03101	1	2	3	4	5	_____ 03102
**32.** Aprender una nueva tarea, como por ejemplo llegar a un lugar en donde nunca ha estado?	|__| 03201	1	2	3	4	5	_____ 03202
**33.** Entender en general lo que dice la gente?	|__| 03301	1	2	3	4	5	_____ 03302
**34.** Iniciar o mantener una conversación? (*si ambos aspectos son diversos realice un promedio*)	|__| 03401	1	2	3	4	5	_____ 03402

Si alguno de los ítems comprendidos entre el 29 y el 34 se puntúa por encima de ninguna (1), pregunte:

**35.** ¿Cuánto han interferido estas dificultades con su vida?	|__| 03501	1	2	3	4	5

II-4. Capacidad para moverse en su alrededor/entorno

Ahora voy a preguntarle acerca de dificultades a la hora de moverse en su alrededor/entorno

Muestre las tarjetas 1 y 2
Si alguno de los ítems se puntúa con "1" (ninguna dificultad, pregunte:* ¿Cuántos días ha estado presente esta dificultad? *y anote el número de días (0-30)

En los últimos 30 días, ¿cuánta dificultad ha tenido para:		Ninguno/a	Leve	Moderado/a	Grave	Extremo/a No puedo hacerlo	
**36.** Estar de pie durante largos periodos de tiempo, como por ejemplo 30 minutos?	|__| 03601	1	2	3	4	5	_____ 03602
**37.** Ponerse de pie cuando estaba sentado/a?	|__| 03701	1	2	3	4	5	_____ 03702
**38.** Moverse dentro de su casa?	|__| 03801	1	2	3	4	5	_____ 03802
**39.** Salir de su casa? (*aunque haya sido a causa de síntomas de ansiedad o depresión*)	|__| 03901	1	2	3	4	5	_____ 03902
**40.** Caminar largas distancias, como un kilómetro (o equivalente)?	|__| 04001	1	2	3	4	5	_____ 04002

Si alguno de los ítems comprendidos entre el 36 y el 40 se puntúa por encima de ninguna (1), pregunte:

**41.** ¿Cuánto han interferido estas dificultades con su vida?	|__| 04101	1	2	3	4	5

II-5. Cuidado personal

Ahora voy a preguntarle acerca de dificultades en su cuidado personal

Muestre las tarjetas 1 y 2
Si alguno de los ítems se puntúa con "1" (ninguna dificultad, pregunte:* ¿Cuántos días ha estado presente esta dificultad? *y anote el número de días (0-30)

En los últimos 30 días, ¿cuánta dificultad ha tenido para:

			Ninguno/a	Leve	Moderado/a	Grave	Extremo/a No puedo hacerlo		
42.	Bañarse (lavarse todo el cuerpo)?	|__| 04201	1	2	3	4	5	_____	04202
43.	Vestirse?	|__| 04301	1	2	3	4	5	_____	04302
44.	Comer?	|__| 04401	1	2	3	4	5	_____	04402
45.	Estar solo/a durante unos días?	|__| 04501	1	2	3	4	5	_____	04502

Si alguno de los ítems comprendidos entre el 42 y el 45 se puntúa por encima de ninguna (1), pregunte:

46.	¿Cuánto han interferido estas dificultades con su vida?	|__| 04601	1	2	3	4	5

II-6. Relacionarse con otras personas

Ahora voy a preguntarle acerca de dificultades para relacionarse con otras personas. Por favor, recuerde que solo le estoy preguntando acerca de dificultades debidas a problemas de salud, tales como enfermedades o lesiones, problemas mentales o emocionales, o problemas relacionados con el uso de alcohol o drogas.

Muestre las tarjetas 1 y 2
Si alguno de los ítems se puntúa "1" (ninguna dificultad, pregunte:* ¿Cuántos días ha estado presente esta dificultad? *y anote el número de días (0-30)

En los últimos 30 días, ¿cuánta dificultad ha tenido para:

			Ninguno/a	Leve	Moderado/a	Grave	Extremo/a No puedo hacerlo		
47.	Relacionarse con personas que no conoce?	|__| 04701	1	2	3	4	5	_____	04702
48.	Mantener una amistad?	|__| 04801	1	2	3	4	5	_____	04802
49.	Llevarse bien con personas cercanas a usted?	|__| 04901	1	2	3	4	5	_____	04902
50.	Hacer nuevos amigos?	|__| 05001	1	2	3	4	5	_____	05002
51.	Tener relaciones sexuales? (*Se incluyen expresiones de afectividad como caricias, besos, etc.*)	|__| 05101	1	2	3	4	5	_____	05102

Si alguno de los ítems comprendidos entre el 47 y el 51 se puntúa con "1" (ninguna dificultad, pregunte:

52.	¿Cuánto han interferido estas dificultades con su vida?	|__| 05201	1	2	3	4	5

II-7. Actividades de la vida diaria

- Quehaceres de casa

Las siguientes preguntas se refieren a actividades que implican el realizar los quehaceres de la casa y cuidar de las personas que conviven con Vd. o que le son cercanas. Estas actividades incluyen cocinar, limpiar, hacer las compras, llevar las cuentas de la casa, realizar pequeñas reparaciones, pagar las cuentas, sacar la basura, así como el cuidado de otras personas y de sus propias pertenencias.

53. Generalmente, ¿cuántas horas a la semana dedica a estas actividades?
Núm. de horas___ / ____ 05101

Muestre las tarjetas 1 y 2
Si alguno de los ítems se puntúa con "1" (ninguna dificultad, pregunte: **¿Cuántos días ha estado presente esta dificultad?** ***y anote el número de días (0-30)***

Debido a su estado de salud, en los últimos 30 días, ¿cuánta dificultad ha tenido para:		Ninguno/a	Leve	Moderado/a	Grave	Extremo/a No puedo hacerlo	
**54.** Cumplir con sus quehaceres de la casa?	|__| 05401	1	2	3	4	5	_____ 05402
**55.** Realizar bien sus quehaceres de la casa más importantes?	|__| 05501	1	2	3	4	5	_____ 05502
**56.** Acabar todo el trabajo de la casa que tenía que hacer?	|__| 05601	1	2	3	4	5	_____ 05602
**57.** Acabar sus quehaceres de la casa tan rápido como era necesario?	|__| 05701	1	2	3	4	5	_____ 05702

Si alguno de los ítems comprendidos entre el 54 y el 57 se puntúa con "1" (ninguna dificultad, pregunte:

58. ¿Cuánto han interferido estas dificultades con su vida?
|__| 05801 1 2 3 4 5

59. En los últimos 30 días, ¿durante cuántos días disminuyó o dejó de hacer sus quehaceres de la casa debido a su "condición de salud"? Núm. días ___ / ____ 05901

Si el entrevistado realiza algún tipo de actividad de tipo laboral o que requiera una regularidad o responsabilidad significativas, ya sea remunerada o no (p.ej., voluntariado, ayudar en un negocio familiar, cargo activo en alguna asociación, etc.), realice las preguntas siguientes. En caso contrario vaya a la sección de "Participación en la Sociedad".

II-8. Actividad laboral

Ahora voy a hacerle algunas preguntas relacionadas con su trabajo.

60. Generalmente, ¿cuántas horas a la semana trabaja?
Núm. de horas___ / ____ 06001

Muestre las tarjetas 1 y 2
Si alguno de los ítems se puntúa con "1" (ninguna dificultad: muestre la tarjeta 3, pregunte:* ¿Cuántos días ha estado presente esta dificultad? *y anote el número de días (0-30)

Debido a su estado de salud, en los últimos 30 días, ¿cuánta dificultad ha tenido para:

			Ninguno/a	Leve	Moderado/a	Grave	Extremo/a No puedo hacerlo		
**61.** Llevar a cabo su trabajo diario?	|__|	06101	1	2	3	4	5	_____	06102
**62.** Realizar bien las tareas mas importantes de su trabajo?	|__|	06201	1	2	3	4	5	_____	06202
**63.** Acabar todo el trabajo que necesitaba hacer?	|__|	06301	1	2	3	4	5	_____	06302
**64.** Acabar su trabajo tan rápido como era necesario?	|__|	06401	1	2	3	4	5	_____	06402

65. ¿Ha tenido que reducir su nivel de trabajo debido a su estado de salud? 1 Sí 2 No |__| 06501

66. ¿Ha ganado menos dinero debido a su estado de salud? 1 Sí 2 No |__| 06502

Si alguno de los ítems comprendidos entre el 61 y el 64 se puntúa con "1" (ninguna dificultad, pregunte:

67. ¿Cuánto han interferido estas dificultades con su vida? |__| 06701 1 2 3 4 5

68. En los últimos 30 días, ¿ha perdido medio día o más de trabajo debido a su estado de salud? Núm. días ___ / ___ 06801

II-9. Participación en sociedad

Ahora voy a hacerle algunas preguntas relacionadas con su participación en la sociedad, y con el impacto que su problema de salud ha tenido sobre usted y su familia. Es posible que algunas de estas preguntas se refieran a problemas que comenzaron hace más de un mes. Sin embargo, es importante que a la hora de contestarlas se refiera exclusivamente a lo ocurrido durante los últimos 30 días. De nuevo le recuerdo que a l contestar a cada una de las siguientes preguntas piense en los problemas de salud mencionados con anterioridad: problemas físicos, problemas mentales o emocionales (o de los nervios), o problemas relacionados con el uso de alcohol o drogas.

Por favor, consigne solo las limitaciones sufridas por el participante que sean debidas a problemas de salud

Muestre las tarjetas 1 y 2
Tenga en cuenta que en las preguntas en esta área no se pide que se especifique el número de días

En los últimos 30 días:

			Ninguno/a	Leve Poco	Moderado/a	Grave	Extremo/a No puedo hacerlo
**69.** ¿Cuánta dificultad ha tenido para participar, al mismo nivel que el resto de las personas, en actividades de la comunidad (por ejemplo, fiestas, actividades religiosas u otras actividades)?	|__|	06901	1	2	3	4	5
**70.** ¿Cuánta dificultad ha tenido debido a barreras u obstáculos existentes en su alrededor/entorno?	|__|	07001	1	2	3	4	5
**71.** ¿Cuánta dificultad ha tenido para vivir con dignidad (o respeto) debido a las actitudes y acciones de otras personas?	|__|	07101	1	2	3	4	5
**72.** ¿Cuánto le ha afectado emocionalmente su estado de salud?	|__|	07201	1	2	3	4	5
**73.** ¿Qué impacto económico ha tenido para usted o para su familia su estado de salud?	|__|	07301	1	2	3	4	5

74. ¿Cuánta dificultad ha tenido su familia debido a su estado de salud? |__| 07401 1 2 3 4 5

75. ¿Cuánta dificultad ha tenido para realizar por si mismo/a cosas que le ayuden a relajarse o disfrutar? |__| 07501 1 2 3 4 5

76. ¿Cuánto tiempo ha dedicado a su estado de salud o a las consecuencias del mismo? |__| 07601 1 2 3 4 5

Si alguno de los ítems comprendidos entre el 69 el 76 se puntúa con "1" (ninguna dificultad, pregunte:

77. ¿En qué medida han interferido estas dificultades con su vida? |__| 07701 1 2 3 4 5

78. En los últimos 30 días, ¿durante cuantos días ha tenido esas dificultades? Núm. días ___ / ___ 07801

79. En total en los últimos 30 días, ¿durante cuantos días experimentó alguna de esas dificultades que ha comentado a lo largo de esta entrevista? Núm. días ___ / ___ 07901

Muchas gracias. Ahora me gustaría hacerle algunas preguntas para ver en que se ha basado para contestar de la manera en que lo ha hecho.

80. Cuando contestó a las preguntas relacionadas con las dificultades que ha tenido, ¿se comparó con alguien? |__| 08001 1 2 3 4 5

Si la respuesta es afirmativa, pregunte el ítem siguiente.
Si es negativa vaya al ítem 83 (tiempo de admón.)

81. ¿Con quién se comparó?

1 Consigo mismo(a) cuando era más joven
2 Consigo mismo(a) antes de la "condición de salud"
3 Con sus amigos(as) 3
4 Con otros(as) de mi misma edad y sexo
5 Otros (especifique) ___________________________ 08101 |__| 08102

82. En total en los últimos 30 días, ¿durante cuantos días experimentó alguna de esas dificultades que ha comentado a lo largo de esta entrevista? Núm. días ___ / ___ 08201

Por favor recapitule en voz alta con el entrevistado las respuestas dadas al WHO-DAS II, compruebe que todas las respuestas han sido cumplimentadas correctamente

III. Índice de Katz

Esta prueba no es de carácter auto-referido, por favor realice cuantas averiguaciones considere oportunas para contestarla fiablemente.

Código en caso de ausencia de respuesta:

Por favor complete cada ítem con el número de opción o respuesta apropiados. En caso de ausencia de respuesta utilice el código 99 seguido de alguna de las especificaciones siguientes.

1. *No puede responder por trastorno cognitivo*
2. *No puede hacerlo por limitaciones físicas*
3. *Se niega*
4. *No es apropiado*
5. *Desconoce la respuesta*
6. *Otro*

La "independencia" conlleva la ausencia de supervisión, dirección o ayuda activa de otra persona. La independencia se basa en el estado actual y no en la habilidad potencial. Una persona que se niega a realizar una función, se considera que no la hace aunque sea capaz de hacerla.

84. Baño (ducha o bañera) |__| 08401

1 Independiente: No precisa asistencia (sale y entra por sí mismo del baño o ducha)
2 Dependiente parcial: Necesita ayuda sólo para lavarse una parte del cuerpo (espalda o una extremidad)
3 Dependiente: Necesita ayuda para lavarse más de una parte del cuerpo, para entrar y salir de la bañera o no se lava sólo

85. Vestido |__| 08501

1 Independiente: Coge la ropa de los cajones y armarios se la pone y puede abrocharse
2 Dependiente parcial: Igual al anterior, pero necesita ayuda para atarse los zapatos
3 Dependiente: No se viste por sí mismo o permanece parcialmente desvestido

86. Uso del retrete |__| 08601

1 Independiente: No precisa ningún tipo de ayuda para entrar o salir del cuarto de baño. Usa el retrete, se limpia y se viste adecuadamente. Puede usar un orinal por la noche
2 Dependiente parcial: Necesita ayuda para alguno de los aspectos anteriores
3 Dependiente: Usa orinal o cuña, o precisa ayuda para acceder y utilizar el retrete

87. Movilización (cama-sillón) |__| 08701

1 Independiente: No requiere ayuda para sentarse o levantarse de una silla ni para entrar o salir de la cama (puede utilizar ayudas mecánicas como un bastón)
2 Dependiente parcial: Precisa ayuda para alguno de los aspectos anteriores
3 Dependiente: En cama

88. Continencia (micción, defecación) |__| 08801

1 Independiente: Control completo de la micción y de la defecación
2 Dependiente parcial: Tiene accidentes ocasionalmente
3 Dependiente: Incontinencia total o parcial de la micción y/o defecación. Incluye el control total de dichas funciones mediante enemas, sonda o el empleo reglado de orinal y/o cuña

89. Alimentación |__| 08901

1 Independiente: Lleva la comida del plato a la boca sin ayuda
2 Dependiente parcial: No precisa ayuda para lo anterior pero sí para cortar la carne
3 Dependiente: Necesita ayuda para comer; no come en absoluto o requiere nutrición enteral-parenteral

90. Hacer la compra (ir a la tienda, subir escaleras, superar barreras físicas, seleccionar, pagar y trasportar los artículos al domicilio) |__| 09001

1 Independiente: Realiza la actividad al menos semanalmente
2 Dependiente parcial: La realiza acompañado por otra persona
3 Dependiente: No la realiza o necesita asistencia para alguno de los aspectos

91. Limpiar la casa (quitar el polvo, aspirar o fregar suelos, sacudir las alfombras/felpudo) |__| 09101

1 Independiente: Realiza la actividad al menos quincenalmente
2 Dependiente parcial: Necesita ayuda para limpiar alfombras/felpudo
3 Dependiente: No realiza la actividad o necesita alguna ayuda

92. Transporte (utiliza transportes públicos; autobús, metro o tren) |__| 09201

1 Independiente: Utiliza el transporte público
2 Dependiente parcial: Necesita compañía de otra persona
3 Dependiente: No utiliza el trasporte público

93. Lavar la ropa (lleva la ropa a la lavadora, añade detergente, la saca, la tiende) |__| 09301

1 Independiente: Realiza la actividad al menos una vez al mes
2 Dependiente parcial: Necesita ayuda para tender
3 Dependiente: No la realiza o necesita alguna ayuda, excepto para tender

94. Cocinar (va a la cocina, prepara la cena, maneja los fuegos |__| 09401

1 Independiente: Realiza la actividad al menos tres veces por semana
2 Dependiente parcial: Necesita ayuda en algún aspecto de la actividad
3 Dependiente: No realiza la actividad

D. CALIDAD DE VIDA (EuroQoL)

Código en caso de ausencia de respuesta:

Por favor complete cada ítem con el número de opción o respuesta apropiados. En caso de ausencia de respuesta utilice el código 99 seguido de alguna de las especificaciones siguientes.

1. *No puede responder por trastorno cognitivo*
2. *No puede hacerlo por limitaciones físicas*
3. *Se niega*
4. *No es apropiado*
5. *Desconoce la respuesta*
6. *Otro*

Ahora en lugar de preguntarle por aspectos de su salud relacionados con los últimos 30 días voy a preguntarle sobre su estado de salud en el día de HOY. Indique la respuesta de cada pregunta que mejor describa su estado de salud, sólo es válida una respuesta en cada pregunta.

Lea el enunciado de cada pregunta seguido de las opciones. Repita cuantas veces considere oportunas hasta que el entrevistado seleccione alguna de las respuestas posibles. No reformule verbalmente las respuestas

96. Movilidad |__| 09601

1 No tengo problemas para caminar
2 Tengo algunos problemas para caminar
3 Tengo que estar en la cama

97. Cuidado personal |__| 09701

1 No tengo problemas con el cuidado personal
2 Tengo algunos problemas para lavarme o vestirme
3 Soy incapaz de lavarme o vestirme

98. Actividades cotidianas (p.ej., trabajar, estudiar, hacer las tareas domésticas, actividades familiares o actividades durante el tiempo libre) |__| 09801

1 No tengo problemas para realizar mis actividades cotidianas
2 Tengo algunos problemas para realizar mis actividades cotidianas
3 Soy incapaz de realizar mis actividades cotidianas

99. Dolor/malestar |__| 09901

1 No tengo dolor ni malestar
2 Tengo dolor moderado o malestar
3 Tengo mucho dolor o malestar

100. Ansiedad/depresión |__| 10001

1 No estoy ansioso ni deprimido
2 Estoy moderadamente ansioso o deprimido
3 Estoy muy ansioso o deprimido

101. Comparando con mi estado general de salud durante los últimos 12 meses, mi estado de salud hoy es: |__| 10101

1 Mejor
2 Igual
3 Peor

Para ayudar a la gente a describir mejor lo bueno o malo que es su estado de salud hemos dibujado una escala parecida a un termómetro en la cual se marca con un 100 el mejor estado de salud que pueda imaginarse y con un 0 el peor estado de salud que pueda imaginarse.

Mostrar al participante la tarjeta 4

102. Nos gustaría que nos indicara en esta escala, en su opinión lo bueno o malo que es su estado de salud, en el día de HOY. Por favor, dibuje una línea desde el casillero donde dice "Su estado de salud hoy" (*) hasta el punto del termómetro que en su opinión indique lo bueno o malo que es su estado de salud en el día de HOY. |__| 10201

Mostrar al entrevistado ilustración adjunta
(*) A veces se tiende a valorar el estado de salud actual haciendo una cruz o una marca en la EVA

E. EVALUACIÓN DEL ESTADO NUTRICIONAL (MNA)

Código en caso de ausencia de respuesta:

Por favor complete cada ítem con el número de opción o respuesta apropiados. En caso de ausencia de respuesta utilice el código 99 seguido de alguna de las especificaciones siguientes.

1. *No puede responder por trastorno cognitivo*
2. *No puede hacerlo por limitaciones físicas*
3. *Se niega*
4. *No es apropiado*
5. *Desconoce la respuesta*
6. *Otro*

Ahora voy a hacerle algunas preguntas sobre los alimentos que toma y sobre otros aspectos relacionados con la salud y la alimentación.

Responda a la primera parte del cuestionario indicando la puntuación adecuada para cada pregunta. Sume los puntos correspondientes al cribaje y si la suma es igual o inferior a 11, complete el cuestionario para obtener una apreciación precisa del estado nutricional. Las preguntas que deben ser contestadas por el participante están indicadas por el encabezado "Preguntar al participante". En cada pregunta se especifica la fuente de información que debe consultarse para su contestación (participante, historial clínico, cuidador, juicio profesional).

I. Cribaje del estado nutricional

104.¿Ha perdido el apetito? ¿Ha comido menos por falta de apetito, problemas digestivos, dificultades al masticar, o al tragar en los últimos tres meses? |__| 10401

Pregunte al participante

¿Ha comido menos de lo normal durante los últimos tres meses?
En caso afirmativo: ¿Ha comido menos por falta de apetito o por problemas al masticar o tragar?
En caso afirmativo: ¿Esta comiendo Vd. mucho menos de lo que comía o solo un poco menos?

1 Pérdida de apetito grave (0)
2 Pérdida de apetito moderada (1)
3 No hay pérdida de apetito (2)

105.Pérdida de peso (<3 meses) |__| 10501

Pregunte al participante/historial clínico si es un paciente crónico o vive en una residencia

¿Ha perdido Vd. peso en los últimos tres meses?
¿Ha notado que la ropa le queda mas suelta en la cintura?
¿Cuánto peso cree Vd. que ha perdido?
Facilitar la respuesta diciendo: ¿Más o menos de 3 kilos?

1 Pérdida de peso>3kg (0)
2 No lo sabe (1)
3 Pérdida de peso entre 1 y 3 Kg. (2)
4 No ha habido pérdida de peso (3)

\+

106. Movilidad |__| 10601

Historial clínico/informe del cuidador/informe del participante si es necesario

¿Es Vd. capaz de levantarse del sillón/cama?
¿Es Vd. capaz de salir de casa?

1 De la cama al sillón (0)
2 Autonomía interior (1)
3 Sale del domicilio (2)

\+

107.¿Ha tenido alguna enfermedad aguda o situación de estrés psicológico en los últimos tres meses? |__| 10701

Historial clínico/juicio profesional/pregunte al participante

¿Ha fallecido algún ser querido hace poco?
¿Se ha mudado hace poco?
¿Se ha encontrado Vd. mal últimamente?

Si el historial clínico del paciente especifica alguna enfermedad aguda puntúe "0"

1 Sí (0)
2 No (1)

\+

108. Problemas neuropsicológicos |__| 10801

Historial clínico/juicio profesional/Informe del cuidador

Si el participante se encuentra presenta demencia o confusión mental importante, la precisión de las preguntas 74, 75, 76, 77, 80, 83, 84, 85, 86, 88 y 89 debe ser contrastada con el cuidador o con el personal sanitario según proceda

1 Demencia o depresión grave (0)
2 Demencia o depresión moderada (1)
3 Sin problemas psicológicos (2)

+ ☐

109. Índice de Masa Corporal (IMC.= peso/ talla^2 en Kg. /m^2) |__| 10901

Tomar de los ítems 9 y 10

1 IMC < 19 (0)
2 19 ≤ IMC < 21 (1)
3 21 ≤ IMC < 23 (2)
4 IMC ≥ 23 (3)

+ ☐

= ☐

Si el participante obtiene 11 puntos o menos continuar la evaluación

II. Evaluación del estado nutricional

110. Vida independiente (no vive en un hospital o residencia) |__| 11001

Preguntar al participante

¿Vive Vd. en su casa o en una residencia?

1 No (0)
2 Sí (1)

Esta pregunta hace referencia a las condiciones de vida habituales del individuo (p.ej., no considerar como el hecho de que el paciente esté hospitalizado a consecuencia de un accidente o enfermedad aguda)

111. ¿Toma más de 3 medicamentos al día? |__| 11101

Historial clínico

1 No (0)
2 Sí (1)

Compruébelas respuestas obtenidos en el cuestionario médico, las recetas del paciente, pregunte al personal de enfermería o al médico de referencia cuando proceda para comprobar cuantos fármacos toma el participante al día en la actualidad

112. Úlceras o lesiones cutáneas |__| 11201

Pregunte al participante/Historial clínico

¿Tiene heridas en la piel por estar mucho tiempo en la cama sin moverse?

1 No (0)
2 Sí (1)

Compruebe el historial clínico del paciente si está disponible, o pregunte al personal sanitario si procede para certificar la presencia de ulceras u otras lesiones cutáneas.

113.¿Cuántas comidas completas toma el paciente al día (equivalentes a dos platos completos y postre)? |__| 11301

Pregunte al participante

¿Toma Vd. normalmente desayuno, comida y cena?

1 comida (0)
2 comidas (1)
3 comidas (2)

Se considera que una comida completa requiere sentarse y comer dos o más platos o raciones de comida

114. Consumo de proteínas |__| 11401

Pregunte al participante/pregunte al personal sanitario

¿Consume leche, queso o yogur todos los días?
¿Come huevos y legumbres (lentejas, garbanzos, alubias, habichuelas, etc.)? ¿con qué frecuencia?
¿Come carne, pescado o pollo cada día?

114.a productos lácteos al menos una vez al día? |__| 11402

1 Sí
2 No

114.b huevos o legumbres una o dos veces a la semana? |__| 11403

1 Sí
2 No

114.c carne, pescado o aves diariamente? |__| 11801

1 Sí
2 No

(0,0 = 0 a 1 síes; 0,5 = 2 síes; 1,0 = 3 síes)

115.¿Consume frutas o verduras al menos dos veces al día? |__| 11501

1 No (0)
2 Sí (1)

Pregunte al participante

¿Come Vd. frutas y verduras?
¿Cuántas raciones de frutas y verduras toma Vd. cada día?

Una ración debe clasificarse como: una fruta (manzana, plátano, naranja, etc.), un vaso de zumo de fruta, un plato de verduras (no se incluyen patatas)

116.¿Cuántos vasos de agua u otros líquidos toma al día? (agua, zumo, café, té, leche, cerveza. vino, etc.) |__| 11601

1 Menos de 3 vasos (0,0)
2 De 3 a 5 vasos (0,5)
3 Más de 5 vasos (1,0)

Pregunte al participante

¿Cuántas tazas de café o té bebe Vd. normalmente al día?
¿Bebe Vd. agua, leche o zumo?
¿Bebe Vd. normalmente en un vaso o en una jarra?

Consideramos que el volumen de un vaso de 190 ml y el de una jarra de 260 ml

117. Forma de alimentarse |__| 11701

1 Necesita ayuda (0)
2 Se alimenta solo con dificultad (1)
3 Se alimenta solo sin dificultad (2)

Pregunte al participante/historial clínico/pregunte al cuidador

¿Es Vd. capaz de comer solo?/¿Puede el paciente comer solo?
¿Necesita ayuda para alimentarse?/¿Ayuda Vd. al paciente a alimentarse?

118. Estado nutricional auto-referido |__| 11801

1 Malnutrición grave (0)
2 No lo sabe o malnutrición moderada (1)
3 Sin problemas de nutrición (2)

Pregunte al participante

¿Cree Vd. que está bien alimentado? (*si no lo comprende:* ¿come Vd. bien? ¿toma comidas variadas?)
Facilite la respuesta: ¿esta mal alimentado?/¿come mal?
¿no está seguro?
¿no tiene problemas con la comida?

La respuesta a esta pregunta depende de la opinión del paciente. Si considera que el paciente no es capaz de contestar, recurra a la opinión del cuidador o del personal sanitario

119. En comparación con las personas de su edad, ¿cómo encuentra el paciente su estado de salud? |__| 11901

1 Peor (0,0)
2 No lo sabe (0,5)
3 Igual (1,0)
4 Mejor (2,0)

Pregunte al participante

¿Se encuentra Vd. bien?
Facilite la respuesta: ¿no se encuentra tan bien como otras personas de su edad?
¿no está seguro?
¿se encuentra tan bien como otras personas de su edad?
¿se encuentra mejor que otras personas de su edad?

Nuevamente la respuesta depende de la opinión del participante

120. Circunferencia braquial |__| 12001

Tomar del ítem 13.a

1 CB < 21 (0,0)
2 21 ≤ CB < 22 (0,5)
3 CB ≥ 22 (1,0)

121. Circunferencia de la pantorrilla (CP en cm) |__| 12101

Tomar del ítem 13.b

1 CP < 31
2 CP ≥ 31

Por favor, anote el tiempo transcurrido de la entrevista hasta este momento |_____| 12201

F. FACTORES SOCIALES

Código en caso de ausencia de respuesta:

Por favor complete cada ítem con el número de opción o respuesta apropiados. En caso de ausencia de respuesta utilice el código 99 seguido de alguna de las especificaciones siguientes.

1. *No puede responder por trastorno cognitivo*
2. *No puede hacerlo por limitaciones físicas*
3. *Se niega*
4. *No es apropiado*
5. *Desconoce la respuesta*
6. *Otro*

I. Hogar y relación de parentesco

123. Dígame cuál es el número de personas que viven en su hogar (incluyéndose Vd.):

1 Vive solo/a
2 Vive en compañía |__| 12301

Nº de miembros incluyendo el entrevistado: |__| 12302

124. Ahora vamos a hablar de cada uno de los miembros de su hogar, sin incluirle a Vd. Dígame la edad, el sexo y la relación de convivencia que tienen con Vd. cada uno de los miembros que viven en este hogar, incluido el servicio doméstico si pernocta en el mismo domicilio que Vd.

Leer las relaciones de convivencia y parentesco.
Preguntar: " el siguiente"..." el siguiente", etc., hasta completar.

Miembro nº 1	Edad	|__|	12401	Sexo*	|__|	12402	Relación con el participante**	|__|	12403
Miembro nº 2	Edad	|__|	12404	Sexo	|__|	12404	Relación con el participante	|__|	12406
Miembro nº 3	Edad	|__|	12407	Sexo	|__|	12406	Relación con el participante	|__|	12409
Miembro nº 4	Edad	|__|	12410	Sexo	|__|	12408	Relación con el participante	|__|	12412
Miembro nº 5	Edad	|__|	12413	Sexo	|__|	12410	Relación con el participante	|__|	12415
Miembro nº 6	Edad	|__|	12416	Sexo	|__|	12412	Relación con el participante	|__|	12418
Miembro nº 7	Edad	|__|	12419	Sexo	|__|	12414	Relación con el participante	|__|	12421
Miembro nº 8	Edad	|__|	12422	Sexo	|__|	12416	Relación con el participante	|__|	12424
Miembro nº 9	Edad	|__|	12425	Sexo	|__|	12418	Relación con el participante	|__|	12427
Miembro nº 10	Edad	|__|	12428	Sexo	|__|	12420	Relación con el participante	|__|	12430

(*) 1. Hombre; 2. Mujer
(**) 1. Esposo/a o Pareja; 2. Padre/Madre; 3. Suegro/Suegra; 4. Hijo/Hija; 5. Nuera/Yerno; 6. Nieto/a; 7. Hermano/a; 8. Cuñado/a; 9. Otro Familiar; 10. Empleado/a de Hogar o persona de servicio; 11. Otras Personas

II. Entorno físico: Vivienda

125. ¿Cómo vive el participante en el momento de la entrevista?

A completar por el entrevistador antes de iniciar la entrevista , marque solo una

1 Vive de forma independiente en la comunidad
2 Vive recibiendo apoyo
3 Hospitalizado |__| 12501

126. A continuación voy a citarle una serie de dotaciones que puede tener o no su vivienda y el edificio o finca donde se ubica.

	Sí	No	NC	
Agua corriente	1	2	3	|__| 12601
Agua caliente	1	2	3	|__| 12602
Portería, conserjería	1	2	3	|__| 12603
WC o retrete	1	2	3	|__| 12604
Ducha o bañera	1	2	3	|__| 12605
Instalación de calefacción: central, individual por gas (natural, ciudad), individual eléctrica	1	2	3	|__| 12606
Otros sistemas para elevar la temperatura: estufa butano, estufa eléctrica, brasero, radiadores eléctricos, chimenea, otros	1	2	3	|__| 12607
Lavadora	1	2	3	|__| 12608
Frigorífico	1	2	3	|__| 12609
TV	1	2	3	|__| 12610
Teléfono fijo o móvil	1	2	3	|__| 12611
Automóvil	1	2	3	|__| 12612

127. De los siguientes equipamientos que le voy a leer, ¿con qué facilidad puede acceder a ellos? muy fácil, fácil, ni fácil ni difícil, difícil o muy difícil

Leer y rellenar todos los lugares

**Lugar**	Muy fácil	Fácil	Ni fácil ni difícil	Difícil	Muy difícil	NC	19606
Centro de salud, médico de cabecera	1	2	3	4	5	99	|__| 12701
Transporte público	1	2	3	4	5	99	|__| 12702
Jardines, parques, zonas verdes	1	2	3	4	5	99	|__| 12703
Tiendas (panadería, ultramarinos, ...)	1	2	3	4	5	99	|__| 12704
Centros y clubes sociales, culturales, ...	1	2	3	4	5	99	|__| 12705
Parroquia u otros servicios religiosos	1	2	3	4	5	99	|__| 12706

III. Entorno social: Red, apoyo y participación

III-1. Red familiar y social

128. A lo largo de la semana, ¿con qué frecuencia ve a algún familiar?*:* diariamente, semanalmente, cada quince días, cada mes o menos.

128.a ¿Y con otros familiares?
128.b ¿Y a algún amigo/amiga?

	**128** Hijos	**128.a** Otros Familiares	**128.b** Amigos/as
Nunca	1	1	1
Todos los días	2	2	2
Cada semana	3	3	3
Cada dos semanas	4	4	4
Cada mes	5	5	5
(*No leer*) No tiene amigos ni familiares	6	6	6
	|__| 12801	|__| 12802	|__| 12803

129. A lo largo de la semana, ¿con qué frecuencia habla por teléfono con sus hijos?: todos los días, todas las semanas, cada quince días, cada mes o menos.

129.a ¿Y con otros familiares?
129.b ¿Y con algún amigo/amiga?

	**129.** Hijos	**129.a** Otros Familiares	**129.b** Amigos/as
Nunca	1	1	1
Todos los días	2	2	2
Cada semana	3	3	3
Cada dos semanas	4	4	4
Cada mes	5	5	5
(*No leer*) No tiene amigos ni familiares	6	6	6
	|__| 12901	|__| 12902	|__| 12903

III-2. Apoyo Social

130. Durante los últimos tres meses, ¿con qué frecuencia ha recibido Vd. ayuda de alguna persona ajena a su hogar sin pagar (amigos, vecinos, hijos/hijas u otros familiares que no viven con Vd.) para labores de la casa (*p.ej., en actividades como hacer la compra, preparar comidas, hacer limpieza, planchar, confeccionar o repasar prendas, reformar vivienda, ir al banco, hacer gestiones, acompañar al médico, otros cuidados personales*)?

1 Sí, todos o casi todos los días
2 Sí, algunas veces a la semana
3 Sí, ocasionalmente
4 No ha recibido ayuda aunque la ha necesitado
5 No ha recibido ayuda pero no la ha necesitado (No leer) |__| 13001

131. ¿Y quién ha sido la persona que en mayor medida le ha ayudado?

1 Esposo/a, pareja
2 Hija, Hijo
3 Nuera, Yerno
4 Nieto, Nieta
5 Otros familiares
6 Vecinos/as, portero/a
7 Amigos/as
8 Empleado/a de hogar
9 Empresa de Servicios Sociales Privada
10 Servicios sociales (trabajador/auxiliar)
11 Voluntario/a (Parroquia, Cruz Roja, otros)
12 Otra persona |__| 13101

132. ¿Tiene usted alguna persona especial con quien pueda compartir confidencias y sentimientos, alguien en que usted sienta que puede confiar?

1 Sí
2 No |__| 13201

III-3. Participación Social

133. Ahora voy a leerle una serie de actividades. Me gustaría que me dijese si las realiza y con que frecuencia. ¿Acude usted a centros culturales/sociales de la comunidad o del ayuntamiento?

1 Sí
2 No ***En caso negativo pasar a 102.b*** |__| 13301

133.a ¿Con qué frecuencia?

1 Nunca
2 Todos los días
3 Todas las semanas
4 Cada dos semanas
5 Cada mes |__| 13302

133.b ¿Acude usted a algún tipo de servicio religioso?

1 Sí
2 No ***En caso negativo pasar a 133.d*** |__| 13304

133.c ¿Con qué frecuencia?

1 Nunca
2 Todos los días
3 Todas las semanas
4 Cada dos semanas
5 Cada mes |__| 13305

133.d ¿Participa activamente en alguna asociación o club, del tipo de voluntariado, grupo de apoyo social?

1 Sí
2 No ***En caso negativo pasar a 134*** |__| 13306

133. e ¿Con qué frecuencia?

1 Nunca
2 Todos los días
3 Todas las semanas
4 Cada dos semanas
5 Cada mes |__| 13307

134. ¿Con qué frecuencia sale usted de casa?

1 Nunca
2 Todos los días
3 Todas las semanas
4 Cada dos semanas
5 Cada mes |__| 13401

IV. Estudios, relación con la actividad y ocupación

135. ¿Cuántos años en total estudió en la escuela, instituto, universidad, etc.? años________ 13501

136. ¿Podría decirme cuál es el nivel de estudios más alto terminado por Vd. mismo/a?
¿Y el de la persona con mayor aportación económica a la familia?

**135.** Entrevistado/a	**136** PMAE*	**Nivel de estudios**
1	1	**No sabe leer ni escribir**: Analfabeto (*No leer*)
2	2	**Sin estudios o inferiores a primarios**:<br>Personas que saben leer y escribir pero fueron menos de 5 años a la escuela
3	3	**Educación Primaria Completa** (hasta los 11 ó 12 años):<br>Personas que han completado 5 cursos escolares del Plan Antiguo (incluido ingreso), o 1ª etapa de EGB (1º a 5º)
4	4	**Educación Secundaria de Primer Grado (de 12 a 16 años de edad)**:<br>Bachillerato Elemental, Graduado Escolar, Certificado de Escolaridad; 2ª etapa de EGB (6º a 8º); 1º y 2º de BUP; ESO; FPI
5	5	**Educación Secundaria de Segundo Grado (de 16 a 18 años de edad)**:<br>Bachillerato Superior, 3º de BUP, COU
6	6	**Formación Profesional (a partir de los 16 años):**<br>Oficialías, Maestrías, FPII, FPIII
7	7	**Enseñanza Universitaria: Primer Ciclo:**<br>Profesorado Mercantil, Escuelas Técnicas de Grado Medio, asistentes sociales, ATS, Magisterio, Escuelas Universitarias;<br>Título de Escuelas Técnicas Superiores, Título de Diplomatura, Título de Ingeniero Técnico
8	8	**Enseñanza Universitaria: Segundo y Tercer Ciclo:**<br>Título de Licenciatura, Ingeniería, Doctorado

|__| 13601 |__| 13601

____

(*) Persona de mayor aportación económica a la familia del participante, si esta expresión resulta difícil de comprender al entrevistado utilizar: "persona que pone más dinero en la casa".

137. ¿En cuál de las siguientes situaciones que le voy a mencionar se encuentra ahora Vd.?

137.a ¿Y la persona con mayor aportación económica a la familia?

**137** Entrevistado/a	**137.a** PMAE	**Relación con la actividad**
1	1	Está trabajando
2	2	Está parado habiendo trabajado antes
3	3	Esta jubilado
4	4	Es pensionista
5	5	Es ama de casa / labores de hogar
6	6	Otra situación (especificar: _______________ 13703_______________ 13704)

|__| 13701 |__| 13702

Si el participante ha trabajado antes preguntar:

137.b ¿Cuál es la ocupación o profesión que desempeña en la actualidad o desempeñaba (en el caso de que hayan trabajado antes)? y ¿la de la persona con mayor aportación económica a la familia?

Actividad del entrevistado:

____ 13704

Actividad del PMAE:

____ 13705

138. De entre las siguientes situaciones que voy a mencionar, ¿podría decirme cuál es la suya?

138.a ¿Y la de la persona con mayor aportación económica en la familia?

En caso de estar jubilado/a o parado/a habiendo trabajado antes, refiérase a su última situación, salvo quien NO HA TRABAJADO NUNCA: pensionista, o parado, o estudiante, o ama de casa que no ha trabajado nunca o que lleva 20 años sin trabajar).

**Condición Socioeconómica**	**138.** Entrevistado/a	**138.a** PMAE
**Trabajadores por cuenta propia**		
Agricultor (pequeña explotación)	1	1
Agricultor (gran explotación)	2	2
Autónomo o empresario con 5 empleados o menos	3	3
Autónomo o empresario con 6 a 9 empleados	4	4
Autónomo o empresario con 10 o más empleados	5	5
Autónomo o empresario sin asalariados	6	6
**Trabajadores por cuenta ajena**		
Dirección / Gerencia con 5 subordinados o menos	7	7
Dirección / Gerencia con 6 a 9 subordinados	8	8
Dirección / Gerencia con 10 o más subordinados	9	9
Capataz, supervisor, encargado	10	10
Mandos intermedios	11	11
Otros empleados con trabajo fuera de oficina y obrero cualificado	12	12
Otros empleados con trabajo de oficina	13	13
Trabajador manual no cualificado	14	14
**Actividad laboral no reglada**		
Voluntariado o participación en asociaciones o parroquias	15	15
Ayuda en negocio familiar	16	16
Otra	17	17
	|__| 13801	|__| 13802

V. Situación económica

139. ¿Qué persona pone más dinero en la casa?

1 Vd. mismo
2 Esposo/a, pareja
3 Hijo/a
4 Nuera, Yerno
5 Nieto/a
6 Hermano/a
7 Cuñado/a
8 Otro Familiar
9 Otra Persona

|__| 13901

140. ¿Podría decirnos cuanto dinero entra en la casa aproximadamente dentro de las siguientes cantidades?

Leer todas las respuestas

140. a ¿Y los de la persona con mayor aportación económica en la familia?

**140.** Entrevistado/a	**140.a** PMAE	**Nivel de ingresos**
1	1	Menos de 300€ (Menos de 50.000 Pts)
2	2	De 301 a 600€ (De 50.001 a 100.000 Pts)
3	3	De 601 a 900€ (de 100.001 a 150.000 Pts)
4	4	De 901 a 1200€ (de 150.001 a 200.000 Pts)
5	5	Más de 1.200 € (Más de 200.000 Pts)
99	99	(*No leer*) No tiene ingresos

|__| 14001 |__| 14002

141. Teniendo en cuenta todos los bienes del hogar (incluyendo vivienda, ahorros, coche, segunda residencia, etc.), ¿dónde situaría económicamente su hogar, no a Vd. personalmente, sino su hogar en una escala de 1 a 10, en donde el 1 sería un hogar muy pobre y el 10 sería un hogar muy rico?

1 Baja
2 Baja
3 Media Baja
4 Media Baja
5 Media
6 Media
7 Media Alta
8 Media Alta
9 Alta
10 Alta |__| 14101

Por favor, anote el tiempo transcurrido de la entrevista hasta este momento |_____| 14201

G. AFRONTAMIENTO (SOC)

Código en caso de ausencia de respuesta:

Por favor complete cada ítem con el número de opción o respuesta apropiados. En caso de ausencia de respuesta utilice el código 99 seguido de alguna de las especificaciones siguientes.

1. *No puede responder por trastorno cognitivo*
2. *No puede hacerlo por limitaciones físicas*
3. *Se niega*
4. *No es apropiado*
5. *Desconoce la respuesta*
6. *Otro*

A continuación hay una serie de preguntas sobre varios aspectos de la vida. Cada pregunta tiene siete respuestas posibles. Por favor, indique el número que mejor exprese su respuesta. Los números uno y siete indican las respuestas extremas. Si las palabras que aparecen debajo del uno son ciertas en su caso, haga un círculo alrededor del uno. Si las palabras que aparecen debajo del siete son ciertas en su caso, haga un círculo alrededor del siete. Si su parecer es diferente, indique el número que mejor encaje con su idea de la pregunta. Por favor, de sólo una respuesta por cada pregunta.

Por favor considere el estado cognitivo del participante durante la administración de esta prueba, así como el posible efecto emocional de algunas de las preguntas. Repita después de cada pregunta la parte subrayada del encabezado relativa a los niveles de respuesta y muestre al participante los distintos niveles en el cuadernillo señalándolos con un bolígrafo. Si lo considera necesario, repita la pregunta, pero no la reformule con intención aclaratoria. Si el participante no da muestras de comprender los tres primeros ítems pase a la sección siguiente. La administración de esta sección es opcional para los grupos de investigación

143. ¿Tiene la impresión de que a Vd. no le importan de verdad las cosas que pasan a su alrededor?

|__| 14301 1 rara vez o nunca — 2 — 3 — 4 — 5 — 6 — 7 muy a menudo

144. ¿Le ha sorprendido alguna vez lo que han hecho personas que Vd. creía que conocía bien?

|__| 14401 1 nunca me ha pasado — 2 — 3 — 4 — 5 — 6 — 7 siempre me pasa

145. ¿Le ha pasado alguna vez que personas con las que contaba le hayan defraudado?

|__| 14501 1 nunca me ha pasado — 2 — 3 — 4 — 5 — 6 — 7 siempre me pasa

146. Hasta ahora su vida:

|__| 14601 1 no ha tenido en absoluto objetivos o metas claras — 2 — 3 — 4 — 5 — 6 — 7 ha tenido objetivos y metas muy claras

147. ¿Tiene Vd. la impresión de que le están tratando de forma injusta?

|__| 14701 1 muy a menudo — 2 — 3 — 4 — 5 — 6 — 7 rara vez o nunca

148. ¿Tiene Vd. la impresión de estar en una situación a la que no está acostumbrado/a y no sabe qué hacer?

|__| 14801 1 muy a menudo — 2 — 3 — 4 — 5 — 6 — 7 rara vez o nunca

149. Hacer las cosas de todos los días:

|__| 14901 1 me produce una gran alegría y satisfacción — 2 — 3 — 4 — 5 — 6 — 7 me produce dolor y aburrimiento

150. ¿Tiene Vd. sentimientos o ideas muy confusas?

|__| 15001 1 muy a menudo — 2 — 3 — 4 — 5 — 6 — 7 rara vez o nunca

151. ¿Le ocurre que tiene sentimientos que preferiría no tener?

|__| 15101 1 muy a menudo — 2 — 3 — 4 — 5 — 6 — 7 rara vez o nunca

152. Muchas personas, hasta los que tienen un carácter fuerte, se sienten a veces desgraciados. ¿Cuántas veces se ha sentido Vd. así en su vida?

	1	2	3	4	5	6	7
|__| 15201	1<br>nunca	2	3	4	5	6	7<br>muy a menudo

153. Cuando algo le ha pasado, al final ha visto Vd. que:

	1	2	3	4	5	6	7
|__| 15301	1<br>le dio más importancia o menos de la que en verdad tenía	2	3	4	5	6	7<br>le dio a las cosas la importancia justa

154. ¿Cuántas veces tiene la impresión de que las cosas que hace todos los días significan muy poco o tienen poca importancia?

	1	2	3	4	5	6	7
|__| 15401	1<br>muy a menudo	2	3	4	5	6	7<br>rara vez o nunca

155. ¿Cuántas veces tiene la impresión de no estar seguro/a de poder controlarse?

	1	2	3	4	5	6	6
|__| 15501	1<br>muy a menudo	2	3	4	5	6	6<br>rara vez o nunca

156. Ahora voy a leerle unas frases sobre distintas formas de ser. Por favor indíqueme si cada una de estas frases le describe a Vd. eligiendo para cada una alguna de las opciones siguientes:

1 En total desacuerdo
2 En desacuerdo
3 Neutral
4 De acuerdo
5 Totalmente de acuerdo

**156.a** No soy una persona que se preocupe mucho	|__|	15601
**156.b** Rara vez me excedo en algo	|__|	15603
**156.c** Soy una persona apacible	|__|	15603
**156.d** Me cuesta resistirme a mis deseos	|__|	15604

Por favor, anote el tiempo empleado en esta sección |_____| 15605

H. CARGA DEL CUIDADOR (Zarit)

Código en caso de ausencia de respuesta:

Por favor complete cada ítem con el número de opción o respuesta apropiados. En caso de ausencia de respuesta utilice el código 99 seguido de alguna de las especificaciones siguientes.

1. *No puede responder por trastorno cognitivo*
2. *No puede hacerlo por limitaciones físicas*
3. *Se niega*
4. *No es apropiado*
5. *Desconoce la respuesta*
6. *Otro*

A continuación se presenta una lista de preguntas que refleja como la gente se siente a veces cuando cuida a otra persona. Después de cada pregunta indique con qué frecuencia se siente de esa forma: nunca, casi nunca, a veces, bastantes veces o casi siempre. No hay preguntas correctas o incorrectas.

1 Nunca
2 Casi nunca
3 A veces
4 Bastantes veces
5 Casi siempre

Por favor considere el posible efecto emocional de algunas de las preguntas de esta prueba. Si fuera necesario procure que el participante no se encuentre presente durante la administración de esta sección o sugiera al cuidador la contestación de esta prueba de forma auto-administrada a objeto de preservar la fiabilidad de la escala

157.¿Siente que su familiar solicita más ayuda de la que realmente necesita? |__| 15701

158.¿Siente que debido al tiempo que dedica a su familiar ya no dispone de tiempo suficiente para usted? |__| 15801

159.¿Se siente tenso cuando tiene que cuidar a su familiar y atender además otras responsabilidades? |__| 15901

160.¿Se siente avergonzado por la conducta de su familiar? |__| 16001

161.¿Cree que la situación actual afecta de manera negativa a su relación con amigos y otros miembros de su familia? |__| 16101

162.¿Siente temor por el futuro que le espera a su familia? |__| 16201

163.¿Siente que su familiar depende de usted? |__| 16301

164.¿Se siente agotado cuando tiene que estar junto a su familiar? |__| 16401

165.¿Siente que su salud se ha resentido por cuidar a su familiar? |__| 16501

166.¿Siente que no tiene la vida privada que desearía debido a su familiar? |__| 16601

167.¿Cree que sus relaciones sociales se han visto afectadas por tener que cuidar de su familiar? |__| 16701

168.¿Se siente incómodo al invitar amigos a casa, por su familiar? |__| 16801

169.¿Cree que su familiar espera que usted le cuide, como si fuera la única persona con la que puede contar? |__| 16901

170.¿Cree que no dispone de dinero suficiente para cuidar a su familiar además de sus otros gastos? |__| 17001

171.¿Siente que será incapaz de cuidar a su familiar por mucho más tiempo? |__| 17101

172.¿Siente que ha perdido el control sobre su vida desde que la enfermedad de su familiar se manifestó? |__| 17201

173.¿Desearía poder encargar el cuidado de su familiar a otras personas? |__| 17301

174.¿Se siente inseguro acerca de lo que debe hacer con su familiar? |__| 17401

175.¿Siente que debería hacer más de lo que hace por su familiar? |__| 17501

176.¿Cree que podría cuidar de su familiar mejor de lo que lo hace? |__| 17601

177. En general: ¿Se siente muy sobrecargado por tener que cuidar de su familiar? |__| 17701

Por favor, anote el tiempo empleado en esta sección |_____| 17801

I. AFRONTAMIENTO DEL CUIDADOR (ISPE)

Código en caso de ausencia de respuesta:

Por favor complete cada ítem con el número de opción o respuesta apropiados. En caso de ausencia de respuesta utilice el código 99 seguido de alguna de las especificaciones siguientes.

1. *No puede responder por trastorno cognitivo*
2. *No puede hacerlo por limitaciones físicas*
3. *Se niega*
4. *No es apropiado*
5. *Desconoce la respuesta*
6. *Otro*

Las preguntas que figuran a continuación se refieren a una serie de situaciones a las que frecuentemente se enfrentan los cuidadores de personas mayores. Puede ser que usted se encuentre en alguna de estas situaciones y en otras no. A medida que le vaya planteando cada una de ellas quiero que me diga si en la actualidad la situación planteada está presente y si le produce algún malestar. De ser así le pediré que me indique en qué grado:

0 No presente/Nada de Malestar
1 Algo de malestar
2 Bastante malestar
3 Mucho malestar

Por favor considere el posible efecto emocional de algunas de las preguntas de esta prueba. Si fuera necesario procure que el participante no se encuentre presente durante la administración de esta sección o sugiera al cuidador la contestación de esta prueba de forma auto-administrada a objeto de preservar la fiabilidad de la escala. La administración de esta sección es opcional para los grupos de investigación

179.¿El enfermo no le reconoce o no reconoce a otros seres queridos? |__| 17901

180.¿Le repite continuamente las mismas cosas?, ¿Le hace la misma pregunta repetidamente? |__| 18001

181.¿Se desorienta el enfermo en lugares conocidos o poco conocidos, y no sabe el mes, día u hora en que estamos? |__| 18101

182.¿Siente que no puede comunicarse con el enfermo como antes?

183.¿El enfermo reconoce que tiene algún tipo de problema, por ejemplo, fallos de memoria, o por el contrario dice que no le ocurre nada, que son los demás los responsables de todo lo malo que le pasa? |__| 18201

184.¿Hay momentos en que el enfermo se muestra alterado, inquieto o irritable? |__| 18301

185.¿Hay momentos en que el enfermo se muestra apático, aislado, sin ganas de hacer nada? |__| 18401

186.¿El enfermo ha dejado de controlar sus esfínteres? |__| 18501

187. Le plantea el enfermo problemas a la hora de comer (p.ej., tirar la comida, no querer comer) o a la hora de asearse (no dejar que el cuidador lo asee, tener que estar pendiente de él para que lo haga, ...)? |__| 18601

188.¿Tiene la sensación de que a veces el enfermo hace las cosas por fastidiar, deliberadamente, como siendo en parte consciente, sobre todo en cosas que realmente le molestan (p.ej., cada vez que le ve que va a salir el enfermo dice que le duele algo), interpretándolo Vd. como que lo hace para que no salga a la calle y incomodarle? |__| 18701

189.¿Tiene la sensación de no tener a nadie a quién le pueda contar como se siente, con quién pueda descargarse emocionalmente? |__| 18801

Por favor, anote el tiempo empleado en esta sección |_____| 18901

190. Este cuaderno B ha sido contestada por:

1 Por el participante
2 Por un familiar o amigo, especificar ____________________________________ 19001
1: Esposo/a, Pareja; 2: Padre/Madre, Suegro/Suegra; 3: Hijo/a; 4: Yerno, Nuera; 5: Nieto/a; 6: Hermano/a, Cuñado/a; 7: Otro Familiar; 8: Empleado/a de hogar, persona de servicio;9: Otras Personas; 10: el entrevistador

3 Por el participante con la ayuda de un familiar o amigo, especificar _________________________ 19002
(En caso de que el participante haya requerido ayuda en al menos una sección)

|__| 19003

En caso de que la entrevista haya sido contestada con la ayuda de un familiar o amigo, especificar (marque un uno en la casilla correspondiente):

190.a Secciones contestadas fundamentalmente por el entrevistado (*1. Si; 2. No*)

1	Identificación del entrevistado	|__|	19004
1	Medidas antropométricas	|__|	19005
1	Discapacidad (WHODAS II)	|__|	19006
1	Calidad de vida (EuroQol)	|__|	19007
1	Estado nutricional (MNA)	|__|	19008
1	Factores sociales	|__|	19009
1	Afrontamiento (SOC)	|__|	19010
1	Carga del cuidador (ZARIT)	|__|	19011
1	Afrontamiento del cuidador (ISPE)	|__|	19012

190.b Secciones contestadas fundamentalmente por el familiar o amigo (*1. Si; 2. No*)

1	Identificación del entrevistado	|__|	19013
1	Medidas antropométricas	|__|	19014
1	Discapacidad (WHODAS II)	|__|	19015
1	Calidad de vida (EuroQol)	|__|	19016
1	Estado nutricional (MNA)	|__|	19017
1	Factores sociales	|__|	19018
1	Afrontamiento (SOC)	|__|	19019
1	Carga del cuidador (ZARIT)	|__|	19020
1	Afrontamiento del cuidador (ISPE)	|__|	19021

Por favor si no obtuvo el consentimiento informado por escrito al comienzo de la entrevista obténgalo en este momento.

Por favor revise rápidamente el cuadernillo y cerciórese de que todas las secciones han sido cumplimentadas correctamente y todos los ítems han sido administrados o, en caso de ausencia de respuesta, han sido codificados adecuadamente.

J. MATERIALES ADICIONALES

Utilice las siguientes tarjetas para la administración del WHO-DAS II

Tarjeta 1:

"Estados de Salud":

- Enfermedades u otros problemas de salud
- Lesiones
- Problemas mentales o emocionales (o de los nervios)
- Problemas relacionados con el alcohol
- Problemas relacionados con las drogas

Tener dificultades en una actividad quiere decir:

- Aumento de esfuerzo
- Malestar o dolor
- Lentitud
- Cambios en el modo en que realiza la actividad
- Tenga en cuenta sólo los últimos 30 días

Tarjeta 2:

1	2	3	4	5
**Ninguno**	**Ligero/a**	**Moderado/a**	**Grave**	**Completo/a**
**Nada**	**Un poco**	**Regular**	**Mucho/a**	**Muchísimo/a**

Tarjeta 3:

**A**	**Un día**		
**B**	**Hasta una semana**	**=**	**De 2 a 7 días**
**C**	**Hasta dos semanas**	**=**	**De 8 a 14 días**
**D**	**Más de dos semanas**	**=**	**De 15 a 29 días**
**E**	**Todos los días**	**=**	**30 días**

Utilice la siguiente tarjeta para la administración del EuroQoL

Tarjeta 4:

El mejor estado de salud imaginable

Para ayudar a las personas a describir lo bueno o malo que es su estado de salud hemos dibujado una escala parecida a un termómetro en la cual se marca con un 100 el mejor estado de salud que pueda imaginarse y con un 0 el peor estado de salud que pueda imaginarse.

Nos gustaría que nos indicara en esta escala, en su opinión, lo bueno o malo que es su estado de salud en el día de HOY. Por favor, dibuje una línea desde el casillero donde dice "Su estado de salud hoy" (*) hasta el punto del termómetro que en su opinión indique lo bueno o malo que es su estado de salud en el día de HOY.

SU ESTADO DE SALUD HOY

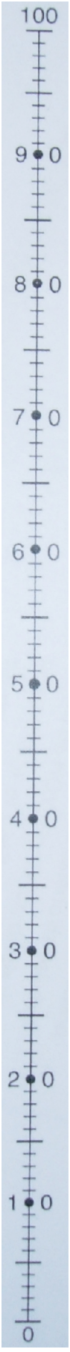

(*) A veces se tiende a valorar el estado de salud actual haciendo una cruz o una marca en el EVA

El peor estado de salud imaginable
